# Pyrrolo[2,3-*e*]indazole as a novel chemotype for both influenza A virus and pneumococcal neuraminidase inhibitors†

**DOI:** 10.1039/d3ra02895j

**Published:** 2023-06-21

**Authors:** Anna Egorova, Martina Richter, Maria Khrenova, Elisabeth Dietrich, Andrey Tsedilin, Elena Kazakova, Alexander Lepioshkin, Birgit Jahn, Vladimir Chernyshev, Michaela Schmidtke, Vadim Makarov

**Affiliations:** a Federal Research Centre “Fundamentals of Biotechnology” of the Russian Academy of Sciences (Research Centre of Biotechnology RAS) 33-2 Leninsky Prospect 119071 Moscow Russia makarov@inbi.ras.ru; b Department of Medical Microbiology, Section of Experimental Virology, Jena University Hospital Hans-Knöll-Straße 2 07745 Jena Germany michaela.schmidtke@med.uni-jena.de; c Chemistry Department, Lomonosov Moscow State University 1-3 Leninskie Gory 119991 Moscow Russia; d Frumkin Institute of Physical Chemistry and Electrochemistry of the Russian Academy of Sciences 31-4 Leninsky Prospect 119071 Moscow Russia

## Abstract

Influenza infections are often exacerbated by secondary bacterial infections, primarily caused by *Streptococcus pneumoniae*. Both respiratory pathogens have neuraminidases that support infection. Therefore, we hypothesized that dual inhibitors of viral and bacterial neuraminidases might be an advantageous strategy for treating seasonal and pandemic influenza pneumonia complicated by bacterial infections. By screening our in-house chemical library, we discovered a new chemotype that may be of interest for a further campaign to find small molecules against influenza. Our exploration of the pyrrolo[2,3-*e*]indazole space led to the identification of two hit compounds, 6h and 12. These molecules were well-tolerated by MDCK cells and inhibited the replication of H3N2 and H1N1 influenza A virus strains. Moreover, both compounds suppress viral and pneumococcal neuraminidases indicating their dual activity. Given its antiviral activity, pyrrolo[2,3-*e*]indazole has been identified as a promising scaffold for the development of novel neuraminidase inhibitors that are active against influenza A virus and *S. pneumoniae*.

## Abstract 2

Pyrrolo[2,3-*e*]indazole is a promising scaffold for the development of novel neuraminidase inhibitors that are active against influenza A virus and *S. pneumoniae*.

## Graphical abstract

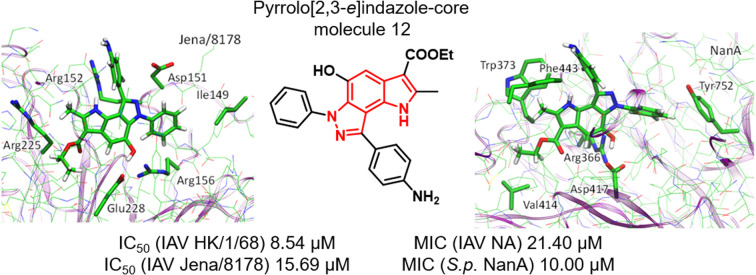

Influenza A and B viruses cause seasonal epidemics of acute respiratory diseases with an annual death mortality of 290 000–650 000 people.^1^ Moreover, there have been at least five influenza A virus pandemics in the past 150 years, such as the Spanish flu (1918–1920), Asian flu (1957–1958), Hong Kong flu (1968–1969), Russian flu (1977–1979) and swine flu (2009–2010), claiming millions of lives.^2–4^ These viral infections are often exacerbated by co/secondary bacterial infections, most commonly caused by *Streptococcus pneumoniae*, *Staphylococcus aureus*, *Streptococcus pyogenes*, or *Haemophilus influenzae*.^5,6^ Indeed, such a secondary infection may cause high mortality from severe pneumonia. Historical records and several systematic reviews indicate that a high number of deaths during the influenza pandemics were the result of secondary bacterial pneumonia rather than primary influenza infection.^7–16^ Co/secondary bacterial infection was identified in 25–95% of cases during the 1918, 1968, and 2009 pandemics, with *S. pneumoniae* being the most common pathogen.^14–16^ Murine studies have shown that influenza A virus promotes *S. pneumoniae* transmission and infection,^17^ demonstrating a lethal synergism between the virus and the bacterium.^18,19^ Hence, therapeutic approaches targeting both pathogens would enable the improvement of global healthcare systems against future respiratory epidemics and pandemics.

Neuraminidase (NA) is an important target in modern anti-influenza drug discovery campaigns.^20–22^ Neuraminidase is a key enzyme in an influenza virus lifecycle: it facilitates viral release and spread. Its function is to catalyze the hydrolysis of sialic acid residues from host-cell glycoprotein receptors.^23,24^ Influenza virus neuraminidase supports *S. pneumoniae* infection, released sialic acids can be catabolized by these bacteria.^25,26^ Moreover, the resulting de-sialylated glycoproteins can be used by *S. pneumoniae* to bind to lung epithelial cells.^5,27–29^*S. pneumoniae* also express neuraminidases – NanA, NanB (both found in most strains), and NanC (rarely identified).^30^ NanA and NanB play important roles in host colonization.^31,32^ The active sites of influenza virus and pneumococcal neuraminidases show structural similarity, suggesting that *S. pneumoniae* could be targeted by neuraminidase inhibitors.^33,34^ Indeed, oseltamivir is active against both viral and bacterial neuraminidases *in vitro* and improves survival in a mouse model of influenza A virus–*S. pneumoniae* synergism.^35,36^ Another NA inhibitor, peramivir, also showed efficacy in influenza virus-infected mice co-infected with *S. pneumoniae*, demonstrating prolonged survival rate and reduced bacterial burden and virus titre.^37^ However, no studies aimed to determine the effect of these drugs on the treatment or prevention of secondary bacterial complications following influenza infection in humans. Although resistance to these drugs occurs infrequently, resistant influenza viruses can emerge and become a major problem as seen in case of oseltamivir in the 2008 influenza season.^38–40^ Therefore, the search for novel compounds with activity against both influenza virus and pneumococcal neuraminidases may be an attractive way for the development of dual-acting anti-infective agents.

In the ongoing anti-influenza virus screening for novel dual-active neuraminidase inhibitors, we found that compounds based on a previously unexplored chemotype, pyrrolo[2,3-*e*]indazole, prevent virus replication by inhibiting the activity of viral neuraminidase. We then obtained a series of pyrrolo[2,3-*e*]indazole-core compounds with various substituents at positions R_1_–R_4_ to study structure–activity relationship. Pyrrolo[2,3-*e*]indazole is a heterocyclic system rarely mentioned in the scientific literature. We synthesized these compounds using the aza-Nenitzescu reaction as previously described by Lyubchanskaya and colleagues.^41,42^ According to Scheme 1, commercially available 1,4-benzoquinone 1 is treated with the corresponding benzaldehyde phenylhydrazones 2a–f to afford hydroquinone adducts 3a–f. Subsequent oxidation of 3a–f with an aqueous solution of potassium ferrocyanide and potassium carbonate leads to indazolequinones 4a–f.^42^ These intermediates are then reacted with the corresponding commercially available aminocrotonic esters 5a–d in the presence of acetic acid and acetic anhydride to cyclize into the final pyrrolo[2,3-*e*]indazoles 6a–k.^41^

**Scheme 1:**
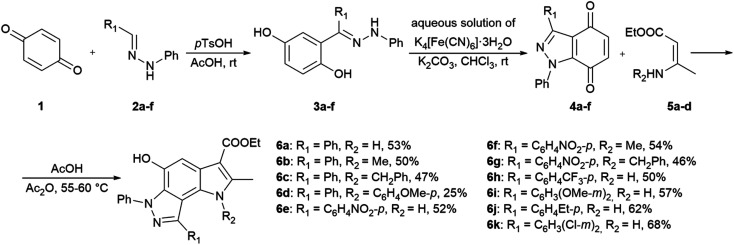
Synthesis of unsubstituted and substituted derivatives of 4-phenyl-pyrrolo[2,3-*e*]indazole 6a–k.

2-Aminoalkyl-pyrrolo[2,3-*e*]indazoles 9a–c and 10a,b were synthesized according to Scheme 2 from pyrroloindazoles 6b,c. The protection of the 5-hydroxy groups of 6b,c with an acetyl group followed by bromination with bromosuccinimide (NBS) in the presence of benzoyl peroxide provides the corresponding 2-bromomethyl derivatives 8a,b. Further nucleophilic substitution of the bromine atom with piperidine, diethylamine, or morpholine results in the 2-substituted derivatives 9a–c. Deprotection of the acetyl group in 9a,c under basic condition gives 2-hydroxypyrrolo[2,3-*e*]indazoles 10a,b.

**Scheme 2:**
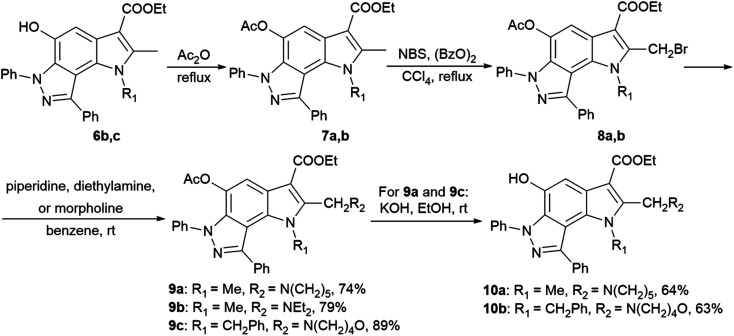
Synthesis of 2-aminoalkyl-pyrrolo[2,3-*e*]indazoles 9a–c and 10a,b.

Using 4-nitrophenyl-pyrroloindazole 6e as a starting point, we synthesized a number of derivatives according to Scheme 3. *O*-Acylation with acetic anhydride of compound 6e yields 5-acetyloxy-4-nitrophenyl-pyrroloindazole 11e. Mono-(5-C) and di-substituted (1,5-C) derivatives 11a–d were synthesized *via* alkylation of 6e with the corresponding alkyl iodides in the presence of potassium carbonate in NMP medium. Further, we reduced the nitro group of compound 6e by catalytic hydrogenation with Pd/C to obtain 4-aminophenyl-pyrroloindazole 13. As for 6e, we performed an *O*-acylation with acetic anhydride for compound 12 to form 5-acetyloxy-4-aminophenyl-pyrroloindazole 13. The pyrrole derivative 14 was prepared by condensation between primary amine of 12 and 2,5-dimethoxytetrahydrofuran in the presence of acetic acid as a catalyst.

**Scheme 3:**
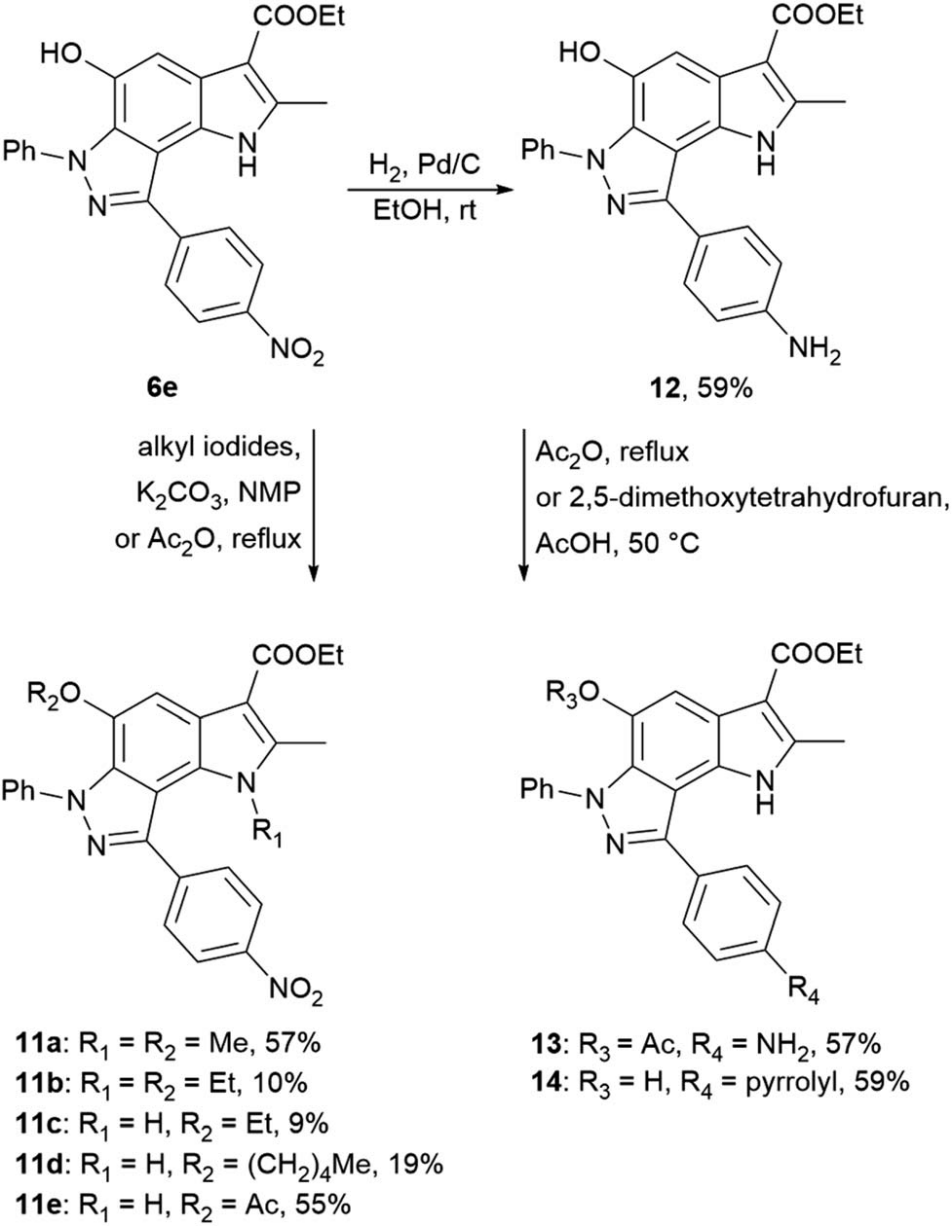
Synthesis of 4-nitrophenyl-, 4-aminophenyl-pyrrolo[2,3-*e*]indazole 6e, 12 and their derivatives 11a–e and 13, 14.

To get a detailed insight into the structural information, X-ray diffraction analysis for compounds 6a and 12 was performed. The crystallographic data were deposited with the Cambridge Crystallographic Data Centre (CCDC ID 2246679 for compound 6a and 2249815 for compound 12).† X-ray crystal structure of 6a established its identity as a 6,8-diphenyl-pyrrolo[2,3-*e*]indazole (Fig. 1A and Table S1†). All bond lengths and angles in compound 12 correspond well to those observed in compound 6a and related compounds from the Cambridge Structural Database (CSD) (Fig. 1B and Table S1†). The triclinic syngony and proximity of unit cell dimensions in both compounds may indicate their crystal packing similarity. Surprisingly, both compounds form similar centrosymmetric hydrogen-bonded dimers despite the different numbers of active sites in their structures. Indeed, the hydroxy group in compound 12 participates in the formation of these dimers similar to the same group in 6a with close geometries (Fig. 1C and D). Specifically, the O1⋯O3(1 − *x*, 2 − *y*, 1 − *z*) distance in 6a is 2.705(4) Å, while in 12 the O1⋯O3(1 − *x*, −*y*, 1 − *z*) distance is 2.711(9) Å. Interestingly, the amino group of compound 12 does not involve in any hydrogen bonding formation. Instead, it participates in weak intermolecular N⋯H⋯π interactions that bind centrosymmetric dimers into ribbon-like stretches (Fig. S2†).

**Fig. 1:**
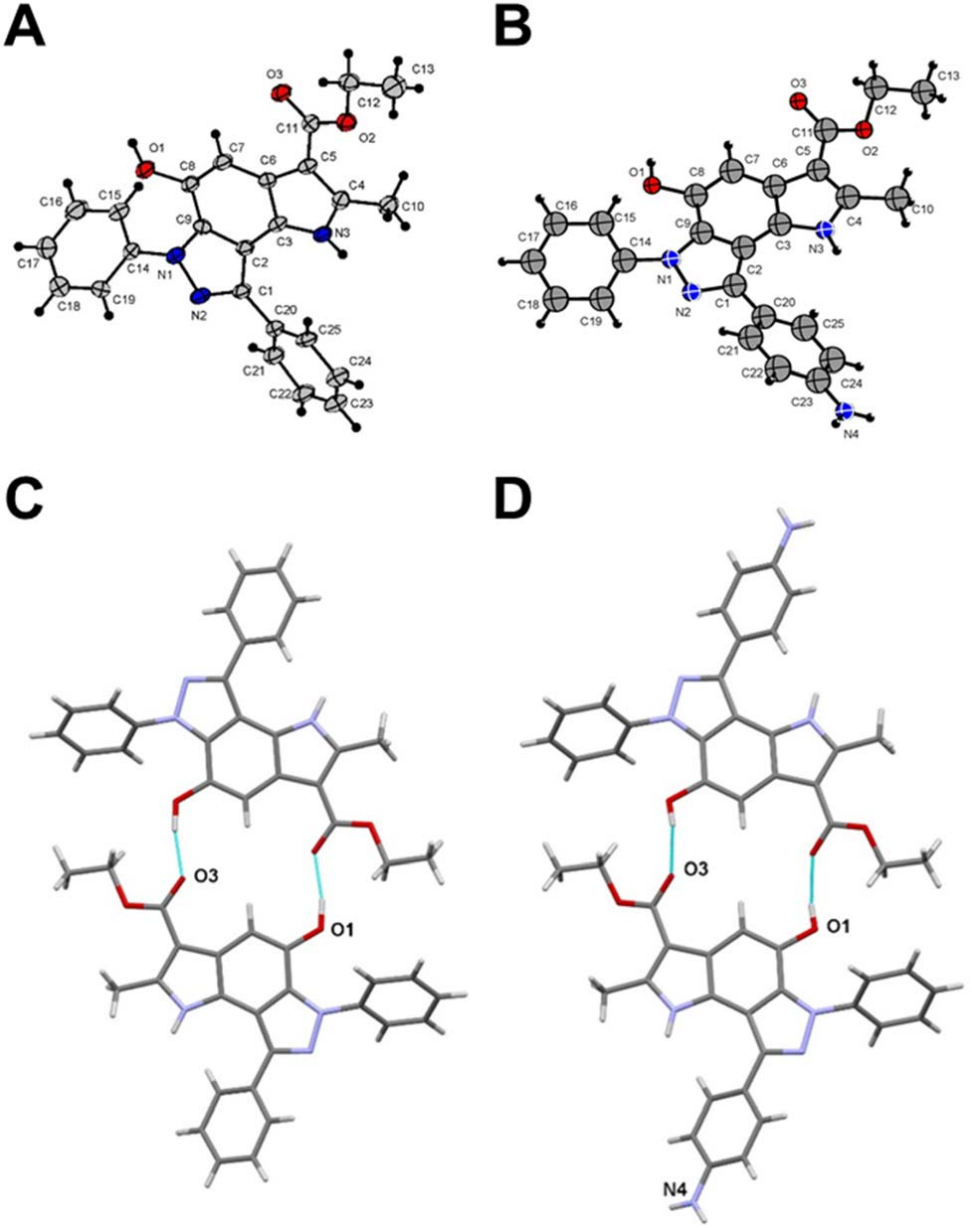
Crystal structure of compound 6a (A) and compound 12 (B). Centrosymmetric hydrogen-bonded dimers in compound 6a (C) and compound 12 (D). Thin cyan lines indicate intermolecular O⋯H⋯O hydrogen bonds.

As a part of an anti-influenza screening, we evaluated synthesized compounds against influenza A virus A/HK/1/68 (subtype H3N2) and influenza A virus A/Jena/8178/09 (subtype A(H1N1)pdm09). Cytotoxicity and antiviral activity of the target compounds on MDCK cells were shown in Table 1. All pyrrolo[2,3-*e*]indazole-core compounds, regardless of structural fragments, showed no cytotoxicity at the maximum tested concentration, 100 μM, for MDCK cells supporting their further study for antiviral activity. The results of the structure–activity relationship study indicate a viral subtype-specific activity of the scaffold. For example, a number of compounds containing different substituents at positions R_2_–R_4_ exclusively inhibited the cytopathic effect induced by influenza virus A/Jena/8178/09 of subtype A(H1N1)pdm09 (compounds 6b–d, 7a,b, 9c, 10a,b, 11a–e, 14). In contrast, pyrroloindazoles with a small group at the R_2_ position (compound 6f) or without substituents at the R_2_–R_4_ positions (compounds 6j,k) exhibited activity only against influenza A virus A/HK/1/68 of subtype H3N2. At the same time, inhibition of both influenza A virus subtypes was shown for compounds without any groups at positions R_2_–R_4_ (exception for compound 13, R_4_ = Ac) and with a R_1_-phenyl ring containing *para*-nitro, amino or trifluoro groups (compounds 6e, 6h, 12). The control compounds oseltamivir and zanamivir acted as expected.

**Table 1:** *In vitro* cytotoxicity and anti-influenza A virus (IAV) activity of pyrrolo[2,3-*e*]indazole-core compounds

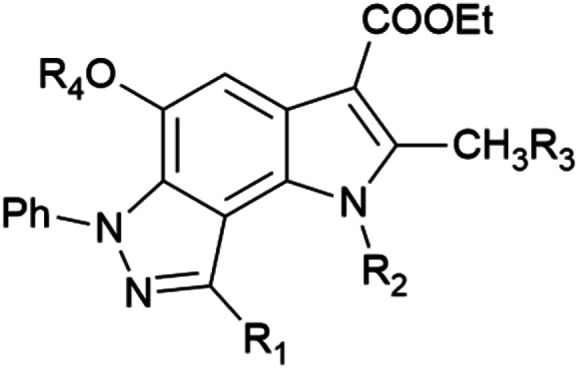							
Cmpd	R_1_	R_2_	R_3_	R_4_	CC_50_ (MDCK), μM	IC_50_ (IAV), μM	
						HK/1/68	Jena/8178
6a	Ph	H	H	H	>100	n.a.^a^	n.a.
6b	Ph	Me	H	H	>100	n.a.	31.60
6c	Ph	CH_2_Ph	H	H	>100	n.a.	71.09
6d	Ph	C_6_H_4_OMe-*p*	H	H	>100	n.a.	38.59
6e	**C**_**6**_ **H**_**4**_ **NO**_**2**_ **-*p***	**H**	**H**	**H**	**>100**	**16.37**	**19.54**
6f	C_6_H_4_NO_2_-*p*	Me	H	H	>100	42.53	n.a.
6g	C_6_H_4_NO_2_-*p*	CH_2_Ph	H	H	>100	n.a.	n.t.^b^
6h	**C**_**6**_ **H**_**4**_ **CF**_**3**_ **-*p***	**H**	**H**	**H**	**>100**	**30.48**	**24.83**
6i	C_6_H_3_(OMe-*m*)_2_	H	H	H	n.t.	n.t.	n.t.
6j	C_6_H_4_Et-*p*	H	H	H	>100	52.23	n.t.
6k	C_6_H_3_(Cl-*m*)_2_	H	H	H	>100	30.18	n.t.
7a	Ph	Me	H	Ac	>100	n.a.	83.73
7b	Ph	CH_2_Ph	H	Ac	>100	n.a.	38.66
9a	Ph	Me	N(CH_2_)_5_	Ac	>100	n.a.	n.a.
9b	Ph	Me	NEt_2_	Ac	>100	n.a.	53.19
9c	Ph	CH_2_Ph	N(CH_2_)_4_O	Ac	n.t.	n.t.	n.t.
10a	Ph	Me	N(CH_2_)_5_	H	>100	n.a.	86.70
10b	Ph	CH_2_Ph	N(CH_2_)_4_O	H	>100	n.a.	33.16
11a	C_6_H_4_NO_2_-*p*	Me	H	Me	>100	n.a.	21.52
11b	C_6_H_4_NO_2_-*p*	Et	H	Et	>100	n.a.	25.48
11c	C_6_H_4_NO_2_-*p*	H	H	Et	>100	n.a.	30.26
11d	C_6_H_4_NO_2_-*p*	H	H	Pentyl	>100	n.a.	26.49
11e	C_6_H_4_NO_2_-*p*	H	H	Ac	>100	n.a.	16.67
12	**C**_**6**_ **H**_**4**_ **NH**_**2**_ **-*p***	**H**	**H**	**H**	**>100**	**8.54**	**15.69**
13	**C**_**6**_ **H**_**4**_ **NH**_**2**_ **-*p***	**H**	**H**	**Ac**	**>100**	**9.39**	**14.08**
14	C_6_H_4_-pyrrolyl	H	H	H	>100	n.a.	11.37
Oseltamivir					n.t.	0.004	0.14
Zanamivir					n.t.	n.t.	0.11

a n.a. – not active.

b n.t. – not tested.

To investigate whether viral hemagglutinin or neuraminidase represent a target for pyrrolo[2,3-*e*]indazole-core compounds, we performed a human erythrocyte-based assay with influenza A virus A/Jena/8178/09. Viral hemagglutinin causes hemagglutination of these erythrocytes at 4 °C, and viral neuraminidase abrogates hemagglutination of human erythrocytes after incubation of the assays at 37 °C. Inhibitors targeting the viral hemagglutinin or neuraminidase might block hemagglutination and/or its abrogation, respectively. With the exception of compound 13 (hemagglutination at 31.6 μM), our compounds inhibited hemagglutination weakly or not at all (results not shown). At the same time, all small molecules were found to inhibit the viral neuraminidase in the assay (Table 2). Among them, compounds 6h, 11a, 11e, 12, and 14 were the most active in the series with MICs in the range of 14.20–24.40 μM. The exception is compound 9a, which does not inhibit either viral neuraminidase activity (Table 2) or viral replication in the CPE reduction assay (Table 1).

**Table 2:** Activity of pyrrolo[2,3-*e*]indazole-core compounds against neuraminidases of influenza A virus (IAV NA) and *Streptococcus pneumoniae* (*S.p.* NanA)

Cmpd	MIC, μM			Cmpd	MIC, μM	
	IAV NA		*S.p.* NanA		IAV NA	*S.p.* NanA
6a	70.00	31.60		9b	77.20	24.40
6b	31.60	31.60		10a	54.40	31.60
6c	77.20	24.40		10b	54.40	10.00
6d	54.40	17.20		11a	24.40	31.60
6e	54.40	14.32		11b	77.20	54.40
6f	77.20	31.60		11c	47.20	54.40
6h	**24.40**	**10.00**		11e	17.20	10.00
7a	100.00	31.60		12	**21.40**	**10.00**
7b	100.00	31.60		13	14.20	n.a.
9a	n.a.	54.40		14	24.40	10.00
Oseltamivir	1.97	2.08^a^		Zanamivir	3.16	n.a.^a^

a Published.^35^

Structural similarities between influenza and pneumococcal neuraminidases in their active sites assume that both neuraminidases can be targeted simultaneously by one neuraminidase small-molecule inhibitor.^33,34^ For example, oseltamivir, but not zanamivir, exerts such dual activity (Table 2).^35,43^ Inspired by the idea of developing small-molecule dual-acting neuraminidase inhibitors, we evaluated whether our pyrroloindazoles would also act on pneumococcal neuraminidase. To do this, we performed a hemagglutination-based NA inhibition assays with the neuraminidase NanA of *S. pneumoniae* strain DSM20566. With the exception of compound 13 all of the compounds tested were found to inhibit the enzyme at moderate concentrations (Table 2). Among them, pyrroloindazoles 6h, 10b, 11e, 12 and 14 have the highest NanA-inhibitory activity with a MIC of 10.0 μM. Taking into account the above results, we represent compounds 6h and 12 as interesting molecules with dual neuraminidase inhibitory activity. Preliminary results of the structure–activity relationship for pyrrolo[2,3-*e*]indazole-core compounds are shown in Fig. 2A.

**Fig. 2:**
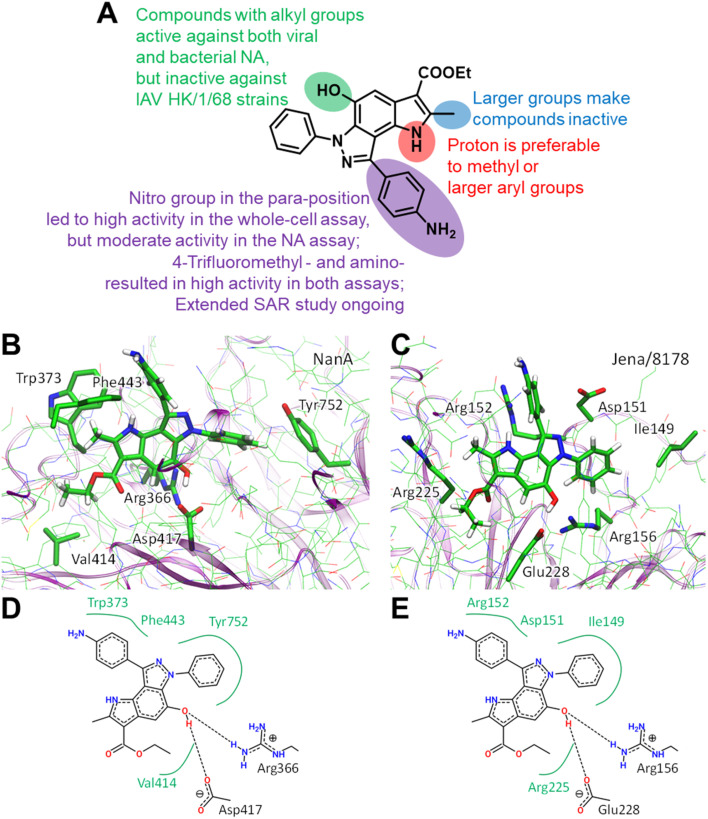
Preliminary SAR results (A). Predicted binding modes ((B and C) 3D representation; (D and E) 2D representation) for compound 12 bound to *Streptococcus pneumoniae* NanA (B and D) and H1N1 (C and E) neuraminidases. In 2D models hydrogen bonds are shown as dashed lines and hydrophobic interactions as green curves. Color code: carbon – green, nitrogen – blue, oxygen – red, hydrogen – white. Hydrogen atoms are shown only for compound 12.

Classic neuraminidase inhibitors, zanamivir and oseltamivir, share the same structural feature, that is the carboxylate group trapping three guanidinium groups of the active site arginine residues.^44–46^ This structural pattern is similar to the transition state, which is formed in the NA active site during the cleavage of sialic acid from glycoconjugates.^47^ In contrast, our compounds do not have such carboxylate substituent in their structures. Moreover, other anionic groups are not presented either. Therefore, the search for a binding site seems challenging, especially as these compounds are active to both viral and bacterial NAs and, therefore, should form stable complexes with two proteins.

We initially performed molecular docking of pyrrolo[2,3-*e*]indazole 12 to the wide area around the active sites of both viral and bacterial neuraminidases. The set of complexes obtained was then studied using classical MD simulations. Most of the complexes dissociated after a short simulation time (less than 10 ns). However, we obtained another binding site that was stable for both NAs for longer than 100 ns (Fig. S1†). Despite the low amino acid sequence similarity between these proteins, we found that compound 12 binds similarly to both of them (Fig. 2). Compound 12 is anchored by two hydrogen bonds with a negatively charged carboxylate, Asp417 in NanA and Glu228 in viral NA, and a positively charged side chain of an arginine residue, Arg366 in NanA and Arg156 in viral NA. Importantly, this arginine residue is not among those that are responsible for substrate carboxylate binding. Compound 12 and its analogues have hydrophobic fragments complementary to the hydrophobic fragments of NA binding sites (Fig. 2).

Another important bacterial NA is NanB, therefore we checked whether this binding site is conserved for NanB. We reconstructed the binding mode similar to that in NanA and viral NA, and performed MD simulation. We found that the complex remains stable, and the structural patterns are the same as for NAs discussed above (Fig. S1†). Thus, we suppose that compound 12 may inhibit NanB as well. However, additional *in vitro* enzyme studies are needed to prove this.

Some studies reported that pneumococcal neuraminidase NanA may impact biofilm formation.^48,49^ So, biofilm formation of *S. pneumoniae* probably can be affected by neuraminidase inhibitors. We have recently observed that some neuraminidase inhibitors suppress bacterial planktonic growth and biofilm formation.^50–52^ In contrast, the influenza drugs oseltamivir and zanamivir do not inhibit either planktonic growth or biofilm formation (Table 3).^50,51^ Similarly, we observed no inhibition of planktonic growth and/or biofilm production of *S. pneumoniae* by most of the pyrrolo[2,3-*e*]indazoles studied (Table 3). Compounds 6c, 6h, and 10a active against bacterial NanA (Table 2) moderately affected *S. pneumoniae* planktonic growth as well as biofilm formation (Table 3). However, there is not enough evidence to conclude whether the inhibition of pneumococcal growth and biofilm formation by these molecules were the result of NanA inhibition.

**Table 3:** Inhibition of planktonic growth and biofilm formation of *Streptococcus pneumoniae* by selected pyrrolo[2,3-*e*]indazole-core compounds

Cmpd	IC_50_, μM	
	Planktonic growth	Biofilm formation
6c	8.90	0.93
6h	50.00	40.13
10a	6.88	3.31
10b	7.57	n.a.
14	n.a.	28.04
Oseltamivir	n.a.	n.a.
Zanamivir	n.a.	n.a.

As a result of our antiviral studies, we have discovered pyrrolo[2,3-*e*]indazoles as a novel class of small molecule inhibitors targeting influenza A virus neuraminidase. According to the results of structure–activity studies, the R_1_-phenyl ring containing *para*-nitro, amino or trifluoro groups of pyrrolo[2,3-*e*]indazoles is important for the inhibition of both circulating influenza A virus subtypes H3N2 and H1N1. Therapeutic advantage of pyrrolo[2,3-*e*]indazoles might represent their dual activity against the viral neuraminidase as well as the structurally related neuraminidase NanA of *S. pneumoniae*. Molecular dynamic simulations demonstrate that the ethyl ester moiety, hydroxy groups, and the pyrrolo[2,3-*e*]indazole core system are responsible for the formation of stable complexes with active sites of both viral and bacterial neuraminidases. Some pyrrolo[2,3-*e*]indazoles also inhibited bacterial growth. Taken together these results led us to conclude that pyrrolo[2,3-*e*]indazole represents novel hit compounds warranting further development.

## Author contributions

AE: investigation, data curation, visualization, writing – original draft, writing – review & editing; MR: investigation; MK: investigation, data curation, visualization, writing – review & editing; ED: investigation; AT: investigation, data curation, formal analysis; EK: investigation; AL: investigation; BJ: investigation; VC: investigation, visualization, writing – review & editing; MS: conceptualization, methodology, resources, data curation, supervision, funding acquisition, writing – original draft, writing – review & editing; VM: conceptualization, methodology, resources, supervision, writing – original draft, writing – review & editing.

All authors discussed the manuscript and approved the submitted version of the paper.

## Conflicts of interest

The authors declare that they have no conflict of interest.

## Supplementary Material

RA-013-D3RA02895J-s001

RA-013-D3RA02895J-s002
