# Supplementary material for: Pyrrolo[2,3-e]indazole as a novel chemotype for both influenza A virus and pneumococcal neuraminidase inhibitors
Source: RSC Adv. 2023 Jun 21;13(27):18253–61. doi: 10.1039/d3ra02895j (PMC10282731; doi:10.1039/d3ra02895j)
Supplement: RA-013-D3RA02895J-s001 [file RA-013-D3RA02895J-s001.pdf]

Electronic Supplementary Material (ESI) for RSC Advances.
This journal is © The Royal Society of Chemistry 2023

SUPPORTING INFORMATION

Pyrrolo[2,3-*e*]indazole as a novel chemotype for both influenza A virus and pneumococcal neuraminidase inhibitors

Anna Egorova[1#], Martina Richter[2#], Maria Khrenova[1,3], Elisabeth Dietrich[2], Andrey Tsedilin[1], Elena Kazakova[1], Alexander Lepioshkin[1], Birgit Jahn[2], Vladimir Chernyshev[3,4], Michaela Schmidtke[2]*, Vadim Makarov[1]*

[1]Federal Research Centre “Fundamentals of Biotechnology” of the Russian Academy of Sciences (Research Centre of Biotechnology RAS), 33-2 Leninsky Prospect, 119071 Moscow, Russia

[2]Department of Medical Microbiology, Section of Experimental Virology, Jena University Hospital, Hans-Knöll-Straße 2, 07745 Jena, Germany

[3]Chemistry Department, Lomonosov Moscow State University, 1-3 Leninskie Gory, 119991 Moscow, Russia

[4]Frumkin Institute of Physical Chemistry and Electrochemistry of the Russian Academy of Sciences, 31-4 Leninsky Prospect, 119071 Moscow, Russia

[#]These authors contributed equally to this paper

*Corresponding authors: makarov@inbi.ras.ru (VM), michaela.schmidtke@med.uni-jena.de (MS)

Table of contents:

Materials and Methods....2
Chemistry General Methods....2
Synthetic Procedures....2
X-Ray Diffraction Analysis....10
Biological Assays....11
Molecular Dynamics Simulations....13
Predicted Binding Mode of Compound 12 to *Streptococcus pneumoniae* NanB....17
^{1}H and ^{13}C Spectra of Final Pyrrolo[2,3-*e*]indazoles....18
Representative HPLC Traces....70
Representative IR Spectra....72
References....74

Materials and Methods

Chemistry General Methods

All reagents and solvents were purchased from commercial suppliers (Sigma-Aldrich, AlfaAesar, Acros, Chimmed) and used without further purification. The ^{1}H and ^{13}C spectra were recorded on a Bruker AC-300 (200 MHz, ^{1}H) or a Bruker AC-200 (50 MHz, ^{13}C) NMR spectrometers. Chemical shifts were measured in DMSO-d_6, using tetramethylsilane as an internal standard, and reported as ppm values. The following abbreviations are used to indicate the multiplicity: s, singlet; d, doublet; t, triplet; m, multiplet; q, quartet; brs, broad singlet; brm, broad multiplet. Mass spectra were recorded on a Finnigan MAT INCOS 50 quadrupole mass spectrometer (EI, 70 eV) with direct injection (Thermo Finnigan, San Jose, CA USA). Purity of the final compounds were analyzed by analytical high-performance liquid chromatography (HPLC) on an Elute HPLC system (Bruker Daltonik, Bremen, Germany) equipped with an Azura UVD 2.1S UV detector (Knauer, Berlin, Germany) with a wavelength at 254 nm and acquisition rate at 1 Hz. Chromatographic separation was carried out on an Acquity HSS T3 column (2.1 × 100 mm, 1.3 µm, 100 Å; Waters, Milford, MA USA) at 30 °C, sample injection volume – 2.0 µL. A mobile phase consisting 0.1 % formic acid in water (A), and 0.1 % formic acid in acetonitrile (B) was programmed with gradient elution of 30-95% over 10 min at a flow rate of 250 µL/min. Data were processed using Compass DataAnalysis 5.1 software (Bruker Daltonik). All final compounds were > 95 % pure. Elemental analysis (% C, H, N) was performed on an EURO EA elemental analyzer (HEKAtech, Wegberg, Germany). IR spectra were recorded on a Bruker ALPHA FT-IR spectrometer (Bruker, Germany) in KBr pellets in the range 4000-400 cm^{-1}. The spectra were processed using the OPUS software. Melting points were determined on an Electrothermal 9001 melting point apparatus (Electrothermal, UK) (10 ºC per min) and were uncorrected. Merck KGaA silica gel 60 F254 plates were used for analytical thin-layer chromatography. Spots were detected by a UV lamp. Column chromatography was performed using silica gel Merck 60 (70-230 mesh). Yields refer to purified products and were not optimized.

Synthetic Procedures

General procedure for the synthesis of mono- and disubstituted benzaldehyde phenylhydrazones **2a-f**. Compounds were synthesized according to the procedure described by Safaei-Ghomi and Masoomi in a slightly modified manner.[1] A solution of the corresponding aldehyde (1 eqv.) and phenylhydrazine (1 eqv.) in EtOH was stirred at rt for 2 h. The precipitated phenylhydrazone was collected and recrystallized from ethanol.

General procedure for the synthesis of hydroquinone-adducts **3a-f**. To a suspension of benzaldehyde phenylhydrazone **2a-f** (1 eqv.) in AcOH, p-toluenesulfonic acid (0.1 eqv.) and 1,4-benzoquinone **1** (1 eqv.) were added, and the resulting mixture was stirred at rt for 24 h. The precipitated hydroquinone-adduct was collected, washed with AcOH and water, and recrystallized from benzene.

General procedure for the synthesis of 3-substituted 1-phenyl-1H-indazole-4,7-diones **4a-f**. To a stirred suspension of **3a-f** (1 eqv.) in chloroform, an oxidizing mixture prepared from potassium

ferrocyanide (1.8 eqv.) and K_2CO_3 (1.7 eqv.) in water was added. The reaction mixture was stirred at rt for 5 h. The organic phase was separated, washed with water and concentrated in vacuo. The residue was purified by column chromatography using benzene as eluent to afford the product **4a-f** as a white solid.

Synthesis of 5-hydroxy-3-ethoxycarbonyl-2-methyl-6,8-diphenyl-pyrrolo[2,3-e]indazole **6a**. Compounds **6a-d** were synthesized as described previously.[2] To a suspension of indazolequinone **4a** (3.00 g, 1.0 eqv.) in AcOH (100 mL) and Ac_2O (1 mL), 3-amino-but-2-enoic acid ethyl ester 5a (1.94 g, 1.5 eqv.) was added at rt, and then the reaction mixture was stirred at 55-60 °C for 24 h. The precipitate was collected, washed with AcOH and water, and dried. Recrystallization from dichloroethane gave the product **6a** as a light yellow solid. Yield 2.17 g (53 %). Mp. 268-271 °C. MS (EI): m/z 411.5. ^{1}H NMR (200 MHz; DMSO-d_6; δ, ppm; *J*, Hz): 1.38 (t, 3H, $CH_2C\underline{H}_3$, *J* = 7.1), 2.68 (s, 3H, 2-CH_3), 4.30 (q, 2H, $C\underline{H}_2CH_3$, *J* = 7.0), 7.26-7.74 (m, 9H, 6-Ph, 8-Ph), 7.86 (m, 2H, H_{ar}, 4-H), 9.70 (brs, 1H, 5-OH), 10.85 (brs, 1H, NH). ^{13}C NMR (50 MHz; DMSO-d_6; δ, ppm): 13.81, 14.50, 58.71, 103.85, 104.00, 110.55, 118.92, 122.27, 126.11, 126.85, 127.80, 128.09, 129.17, 129.92, 133.37, 138.96, 140.94, 141.48, 143.69, 165.18. Anal. calcd for $C_{25}H_{21}N_3O_3$: C, 72.98; H, 5.14; N, 10.21. Found: C, 72.95; H, 5.18; N, 10.15.

5-Hydroxy-3-ethoxycarbonyl-1,2-dimethyl-6,8-diphenyl-pyrrolo[2,3-e]indazole **6b**. Light beige solid. Yield 50 %. Mp. 270-272 °C (dichloroethane). MS (EI): m/z 425.5. ^{1}H NMR (200 MHz; DMSO-d_6; δ, ppm; *J*, Hz): 1.37 (t, 3H, $CH_2C\underline{H}_3$, *J* = 7.1), 2.57 (s, 3H, 2-CH_3), 3.13 (s, 3H, 1-CH_3), 4.30 (q, 2H, $C\underline{H}_2CH_3$, *J* = 7.0), 7.35-7.75 (m, 11H, 6-Ph, 8-Ph, 4-H), 9.76 (brs, 1H, 5-OH). ^{13}C NMR (50 MHz; DMSO-d_6; δ, ppm): 11.85, 14.45, 34.83, 58.81, 103.93, 111.88, 121.48, 122.07, 126.16, 126.89, 127.76, 128.23, 128.55, 130.02, 135.89, 138.93, 140.86, 142.00, 143.63, 165.04. Anal. calcd for $C_{26}H_{23}N_3O_3$: C, 73.39; H, 5.45; N, 9.88. Found: C, 73.42; H, 5.41; N, 9.89.

1-Benzyl-5-hydroxy-3-ethoxycarbonyl-2-methyl-6,8-diphenyl-pyrrolo[2,3-e]indazole **6c**. Light beige solid. Yield 47 %. Mp. 256-258 °C (dichloroethane). MS (EI): m/z 501.6. ^{1}H NMR (200 MHz; DMSO-d_6; δ, ppm; *J*, Hz): 1.38 (t, 3H, $CH_2C\underline{H}_3$, *J* = 7.1), 2.44 (s, 3H, 2-CH_3), 4.31 (q, 2H, $C\underline{H}_2CH_3$, *J* = 7.1), 5.02 (brs, 2H, 1-CH_2Ph), 6.30 (2H, d, $CH_2\underline{Ph}$, *J* = 5.5), 7.00-7.21 (3H, m, $CH_2\underline{Ph}$), 7.21-7.66 (10H, m, 6-Ph, 8-Ph), 7.74 (s, 1H, 4-H), 9.84 (brs, 1H, 5-OH). ^{13}C NMR (50 MHz; DMSO-d_6; δ, ppm): 11.82, 14.40, 48.87, 59.01, 103.91, 105.11, 111.76, 121.75, 122.06, 125.06, 126.23, 126.86, 126.95, 127.71, 128.07, 128.33, 128.51, 129.65, 130.31, 135.62, 137.19, 139.32, 140.80, 141.90, 143.33, 165.00. Anal. calcd for $C_{32}H_{27}N_3O_3$: C, 76.63; H, 5.43; N, 8.38. Found: C, 76.67; H, 5.45; N, 8.35.

5-Hydroxy-3-ethoxycarbonyl-1-(4-methoxyphenyl)-2-methyl-6,8-diphenyl-pyrrolo[2,3-e]indazole **6d**. Light beige solid. Yield 25 %. Mp. 277-279 °C (dichloroethane). MS (EI): m/z 517.6 ^{1}H NMR (200 MHz; DMSO-d_6; δ, ppm; *J*, Hz): 1.38 (t, 3H, $CH_2C\underline{H}_3$, *J* = 7.1), 2.34 (s, 3H, 2-CH_3), 3.67 (s, 3H, 1-CH_3), 4.34 (q, 2H, $C\underline{H}_2CH_3$, *J* = 7.1), 6.47(d, 2H, HC(34), HC(36), *J* = 8.8,), 6.82-7.26 and 7.28-7.68 (m and m, 7H and 5H, 6-Ph, 8-Ph, HC(33), HC(37)), 7.75 (s, 1H, 4-H), 9.85 (s, 1H, 5-OH). ^{13}C NMR (50 MHz; DMSO-d_6; δ, ppm): 13.12, 14.46, 54.84, 59.06, 103.65, 105.09, 114.25, 121.88, 122.43,

126.19, 126.91, 127.27, 127.71, 128.91, 130.52, 135.13, 139.47, 140.87, 142.52, 144.23, 158.45, 165.09. Anal. calcd for $C_{32}H_{27}N_3O_4$: C, 74.26; H, 5.26; N, 8.12. Found: C, 74.34; H, 5.216; N, 8.05.

5-Hydroxy-3-ethoxycarbonyl-2-methyl-8-(4-nitrophenyl)-6-phenyl-pyrrolo[2,3-e]indazole **6e**. The compound was prepared in a similar manner to that described for compound **6a** from indazolequinone **4b** and 3-amino-but-2-enoic acid ethyl ester **5a**. Orange red solid. Yield 52 %. Mp. 312-315 °C (benzene). MS (EI): m/z 456.5. ^{1}H NMR (200 MHz; DMSO-d$_6$; δ, ppm; *J*, Hz): 1.37 (t, 3H, $CH_2C\underline{H_3}$, *J* = 7.1), 2.67 (s, 3H, 2-CH_3), 4.29 (q, 2H, $OC\underline{H_2}CH_3$), 7.28-7.80 (m, 6H, 4-H, 6-Ph), 8.10 and 8.43 (в, 2H and 2H, 8-$C_6H_4NO_2$-*p*, *J* = 8.7), 9.81 (s, 1H, 5-OH), 11.00 (brs, 1H, NH). ^{13}C NMR (50 MHz; DMSO-d$_6$; δ, ppm): 13.89, 14.54, 58.85, 104.08, 104.40, 110.70, 118.60, 122.88, 124.52, 126.30, 127.38, 127.98, 129.02, 130.22, 139.10, 140.02, 140.80, 141.71, 141.83, 147.02, 165.18. Anal. calcd for $C_{25}H_{20}N_4O_5$: C, 65.78; H, 4.42; N, 12.27. Found: C, 65.80; H, 4.39; N, 12.24.

5-Hydroxy-3-ethoxycarbonyl-1,2-dimethyl-8-(4-nitrophenyl)-6-phenyl-pyrrolo[2,3-e]indazole **6f**. The compound was prepared in a similar manner to that described for compound **6a** from indazolequinone **4b** and 3-(methylamino)crotonic acid ethyl ester **5b**. Light beige solid. Yield 54 %. Mp. 258-261 °C (dichloroethane). MS (EI): m/z 470.5. ^{1}H NMR (200 MHz; DMSO-d$_6$; δ, ppm; *J*, Hz): 1.37 (t, 3H, $CH_2C\underline{H_3}$, *J* = 7.0), 2.61 (s, 3H, 2-CH_3), 3.21 (s, 3H, 1-CH_3), 4.30 (q, 2H, $C\underline{H_2}CH_3$), 7.28-7.80 (m, 6H, 6-Ph, 4-H), 7.96 and 8.37 (d, 2H and 2H, 8-$C_6H_4NO_2$, *J* = 8.5), 9.84 (s, 1H, 5-OH). ^{13}C NMR (50 MHz; DMSO-d$_6$; δ, ppm): 11.90, 14.43, 35.74, 58.88, 104.08, 104.23, 111.24, 121.86, 123.47, 126.26, 127.29, 127.85, 130.56, 131.07, 138.98, 140.68, 141.63, 142.32, 142.45, 147.30, 165.00. Anal. calcd for $C_{26}H_{22}N_4O_5$: C, 66.38; H, 4.71; N, 11.91. Found: C, 66.45; H, 4.65; N, 11.87.

1-Benzyl-5-hydroxy-3-ethoxycarbonyl-2-methyl-8-(4-nitrophenyl)-6-phenyl-pyrrolo[2,3-e]indazole **6g**. The compound was prepared in a similar manner to that described for compound **6a** from indazolequinone **4b** and 3-(benzylamino)isocrotonic acid ethyl ester **5c**. Light beige solid. Yield 46 %. Mp. 226-228 °C (benzene). MS (EI): m/z 546.6. ^{1}H NMR (200 MHz; DMSO-d$_6$; δ, ppm; *J*, Hz): 1.38 (t, 3H, $CH_2C\underline{H_3}$, *J* = 7.1), 2.45 (s, 3H, 2-CH_3), 4.31 (q, 2H, $C\underline{H_2}CH_3$), 5.05 (brs, 2H, $\underline{CH_2}Ph$), 6.36, 7.14 and 7.28-7.71 (m, q and m, 2H, 3H and 7H, $CH_2\underline{Ph}$, 6-Ph, 8-$C_6H_4NO_2$), 7.76 (s, 1H, 4-H), 8.01 (d, 2H, 8-$C_6H_4NO_2$, *J* = 8.6), 9.90 (s, 1H, 5-OH). ^{13}C NMR (50 MHz; DMSO-d$_6$; δ, ppm): 11.88, 14.40, 49.24, 59.06, 104.18, 111.27, 121.86, 122.07, 122.86, 124.89, 126.32, 127.05, 127.29, 127.79, 128.26, 128.40, 130.59, 130.86, 136.63, 139.33, 140.63, 141.33, 142.03, 142.12, 147.21, 164.92. Anal. calcd for $C_{32}H_{26}N_4O_5$: C, 70.32; H, 4.79; N, 10.25. Found: C, 70.25; H, 4.84; N, 10.19.

5-Hydroxy-3-ethoxycarbonyl-2-methyl-6-phenyl-8-[4-(trifluoromethyl)phenyl]-pyrrolo[2,3-e]indazole **6h**. The compound was prepared in a similar manner to that described for compound **6a** from indazolequinone **4c** and 3-amino-but-2-enoic acid ethyl ester **5a**. Light beige solid. Yield 50 %. Mp. 228-232 °C (AcOH). MS (EI): m/z 479.5. ^{1}H NMR (500 MHz; DMSO-d$_6$; δ, ppm; *J*, Hz): 1.42 (t, 3H, $CH_2C\underline{H}_3$, *J* = 7.2), 2.70 (s, 3H, 2-CH_3), 4.33 (q, 2H, $C\underline{H_2}CH_3$), 7.44-7.69 (m, 6H, 4-H, 6-Ph), 7.82 (d, 2H, 8-$C_6H_4CF_3$-*p*, *J* = 8.0), 8.07 (d, 2H, 8-$C_6H_4CF_3$-*p*, *J* = 8.0); 9.67 (brs, 1H, 5-OH), 10.95 (s, 1H, NH). ^{13}C NMR (126 MHz; DMSO-d$_6$; δ, ppm): 13.79, 14.46, 58.72, 103.90, 104.13, 110.46, 118.58, 122.54, 126.06, 126.16, 126.39, 127.12, 127.85, 128.47, 130.05, 137.31, 138.95, 140.76, 141.62,

142.18, 165.10. Anal. calcd for $C_{26}H_{20}F_3N_3O_3$: C, 65.13; H, 4.20; N, 8.76. Found: C, 65.16; H, 4.23; N, 8.74. IR (KBr, ν/cm^{-1}): 3440 (OH), 3247 (NH), 1653 and 1639 (C=O), 1326, 1177, 1113, 856.

8-(3,5-Dimethoxyphenyl)-5-hydroxy-3-ethoxycarbonyl-2-methyl-6-phenyl-pyrrolo[2,3-e]indazole **6i**. The compound was prepared in a similar manner to that described for compound **6a** from indazolequinone **4d** and 3-amino-but-2-enoic acid ethyl ester **5a**. Light beige solid. Yield 57 %. Mp. 264-267 °C (AcOH). MS (EI): m/z 471.5. ^{1}H NMR (500 MHz; DMSO-d$_6$; δ, ppm; *J*, Hz): 1.34 (t, 3H, $CH_2C\underline{H_3}$, *J* = 7.1), 2.66 (s, 3H, 2-CH_3), 3.81 (brs, 6H, 2OCH_3), 4.29 (q, 2H, $OC\underline{H_2}CH_3$), 6.61 (s, 1H, 8-$C_6\underline{H_3}$(OMe-*m*)$_2$), 6.97 (s, 2H, 8-$C_6\underline{H_3}$(OMe-*m*)$_2$), 7.35-7.70 (m, 6H, 4-H, 6-Ph), 9.70 (s, 1H, 5-OH), 11.03 (brs, 1H, NH). ^{13}C NMR (126 MHz; DMSO-d$_6$; δ, ppm): 13.57, 14.55, 30.78, 35.79, 55.24, 58.75, 101.23, 103.87, 104.03, 105.43, 110.63, 118.92, 122.31, 126.22, 126.97, 127.89, 129.88, 135.13, 138.99, 140.94, 141.55, 143.68, 160.91, 162.33, 165.23. Anal. calcd for $C_{27}H_{25}N_3O_5$: C, 68.78; H, 5.34; N, 8.91. Found: C, 68.75; H, 5.37; N, 8.93.

8-(4-Ethylphenyl)-5-hydroxy-3-ethoxycarbonyl-2-methyl-6-phenyl-pyrrolo[2,3-e]indazole **6j.** The compound was prepared in a similar manner to that described for compound **6a** from indazolequinone **4e** and 3-amino-but-2-enoic acid ethyl ester **5a**. Light beige solid. Yield 62 %. Mp. 292-295 °C (EtOH/acetone). MS (EI): m/z 439.5. ^{1}H NMR (500 MHz; DMSO-d$_6$; δ, ppm; *J*, Hz): 1.33 (m, 6H, 2CH_3), 2.73 (m, 5H, 2-CH_3, $C\underline{H_2}CH_3$), 4.31 (p, 2H, $C\underline{H_2}CH_3$, *J* = 7.3), 7.25-7.68 (m, 8H, 6-Ph, 8-$C_6\underline{H_4}$Et-*p*), 7.76 (d, 2H, 8-$C_6\underline{H_4}$Et-*p*, *J* = 8.0), 9.66 (s, 1H, 5-OH), 10.83 (s, 1H, NH). ^{13}C NMR (300 MHz; DMSO-d$_6$; δ, ppm): 13.72, 14.42, 15.21, 27.97, 58.72, 102.39, 103.36, 103.49, 110.09, 118.57, 121.77, 125.67, 125.92, 126.38, 127.11, 127.33, 127.39, 127.52, 127.60, 128.16, 129.51, 130.42, 138.57, 140.61, 141.08, 143.20, 143.36, 160.29, 164.89. Anal. calcd for $C_{27}H_{25}N_3O_3$: C, 73.79; H, 5.73; N, 9.56. Found: C, 73.81; H, 5.75; N, 9.53.

8-(3,5-Dichlorophenyl)-5-hydroxy-3-ethoxycarbonyl-2-methyl-6-phenyl-pyrrolo[2,3-e]indazole **6k**. The compound was prepared in a similar manner to that described for compound **6a** from indazolequinone **4f** and 3-amino-but-2-enoic acid ethyl ester **5a**. Light beige solid. Yield 68 %. Mp. 297-300 °C (acetone/EtOH). MS (EI): m/z 480.3. ^{1}H NMR (200 MHz; DMSO-d$_6$; δ, ppm; *J*, Hz): 1.38 (t, 3H, $CH_2C\underline{H_3}$, *J* = 7.0), 2.65 (s, 3H, 2-CH_3), 4.29 (q, 2H, $C\underline{H_2}CH_3$), 7.32-7.88 (m, 9H, 4-H, 6-Ph, 8-C_6H_3(Cl-*m*)$_2$), 9.75 (brs, 1H, 5-OH), 11.17 (s, 1H, NH). ^{13}C NMR (300 MHz; DMSO-d$_6$; δ, ppm): 13.82, 14.52, 58.79, 103.89, 104.16, 110.38, 118.43, 122.52, 125.47, 126.07, 126.52, 127.08, 127.59, 127.79, 127.93, 128.74, 129.84, 134.71, 136.58, 138.89, 140.61, 140.99, 141.60, 165.04. Anal. calcd for $C_{25}H_{19}Cl_2N_3O_3$: C, 62.51; H, 3.99; N, 8.75. Found: C, 62.54; H, 3.97; N, 8.73.

Synthesis of 5-(acetyloxy)-3-ethoxycarbonyl-1,2-dimethyl-6,8-diphenyl-pyrrolo[2,3-e]indazole **7a**. A mixture of pyrroloindazole **6a** (2.18 g, 1 eqv.) and Ac_2O (52 mL) was refluxed for 4 h. After completion of the reaction, the mixture was poured into cold water (100 mL), the precipitate was collected, washed with water and dried. Recrystallization from EtOH gave the product **7a** as a beige solid. Yield 2.29 g (96 %). Mp. 214-216 °C. MS (EI): m/z 467.5 ^{1}H NMR (200 MHz; DMSO-d$_6$; δ, ppm; *J*, Hz): 1.35 (t, 3H, $CH_2C\underline{H_3}$, *J* = 7.1), 1.59 (s, 3H, 5-$OCOCH_3$), 2.64 (s, 3H, 2-CH_3), 3.22 (s, 3H, 1-CH_3), 4.32 (q, 2H, $C\underline{H_2}CH_3$), 7.37-7.83 (m, 10H, 6-Ph, 8-Ph), 8.00 (s, 1H, 4-H). ^{13}C NMR (50 MHz; DMSO-

d_6; δ, ppm): 11.86, 14.38, 19.48, 35.06, 59.16, 104.64, 111.58, 114.19, 120.22, 126.16, 126.78, 128.30, 128.75, 130.11, 130.34, 131.57, 135.29, 139.52, 143.52, 143.79, 164.68, 168.90. Anal. calcd for $C_{28}H_{25}N_3O_4$: C, 71.93; H, 5.39; N, 8.99. Found: C, 71.96; H, 5.41; N, 8.95.

5-(Acetyloxy)-3-ethoxycarbonyl-1-benzyl-2-methyl-6,8-diphenyl-pyrrolo[2,3-e]indazole **7b**. The compound was prepared in a similar manner to that described for compound **7a** from pyrroloindazole **6b**. Beige solid. Yield 97 %. Mp. 165-167 °C (*i*-PrOH). MS (EI): m/z 543.6. ^{1}H NMR (200 MHz; DMSO-d_6; δ, ppm; *J*, Hz): 1.35 (t, 3H, $CH_2C\underline{H}_3$, *J* = 7.1), 1.59 (s, 3H, 5-$OCOCH_3$), 2.62 (s, 3H, 2-CH_3), 4.33 (q, 2H, $C\underline{H}_2CH_3$, *J* = 7.1), 5.13 (brs, 2H, 1-$C\underline{H}_2$Ph), 6.35 and 7.00-7.73 (brs and m, 2H and 13H, 6-Ph, 8-Ph, $CH_2\underline{Ph}$), 8.00 (s, 1H, 4-H). ^{13}C NMR (50 MHz; DMSO-d_6; δ, ppm): 11.82, 14.31, 19.47, 49.08, 59.37, 105.84, 111.45, 114.29, 120.39, 125.01, 126.13, 126.84, 127.03, 128.09, 128.34, 128.45, 128.71, 129.74, 130.71, 131.71, 134.95, 136.71, 139.42, 143.26, 143.41, 164.60, 168.94. Anal. calcd for $C_{34}H_{29}N_3O_4$: C, 75.12; H, 5.38; N, 7.73. Found: C, 75.15; H, 5.35; N, 7.78.

Synthesis of 5-(acetyloxy)-3-ethoxycarbonyl-2-(bromomethyl)-1-methyl-6,8-diphenyl-pyrrolo[2,3-e]indazole **8a**. A mixture of pyrroloindazole **7a** (3.09 g, 1 eqv.), *N*-bromosuccinimide (1.32 g, 1.05 eqv.) and benzoyl peroxide (1 mg) in CCl_4 (59 mL) was refluxed for 5 h. The hot reaction mixture was filtered and the filtrate was concentrated in vacuo. The residue was treated with hexane, the precipitate was collected, washed with hexane and dried. Recrystallization from hexane/benzene mixture gave the product **8a** a white solid. Yield 3.73 g (94 %). Mp. 177-180 °C. MS (EI): m/z 546.4. ^{1}H NMR (200 MHz; DMSO-d_6; δ, ppm; *J*, Hz): 1.37 (t, 3H, $CH_2C\underline{H}_3$, *J* = 7.2), 1.55 (s, 3H, 5-$OCOCH_3$), 3.31 (s, 3H, 1-CH_3), 4.37 (q, 2H, $C\underline{H}_2CH_3$), 4.79 and 4.94 (s, 1H and 1H, CH_2Br), 7.46-7.73 (m, 10H, 6-Ph, 8-Ph), 7.91 (s, 1H, 4-H). Anal. calcd. for $C_{28}H_{24}BrN_3O_4$: C, 61.55; H, 4.43; N, 7.69. Found: C, 60.58; H, 4.49, N, 7.65.

5-(Acetyloxy)-3-ethoxycarbonyl-1-benzyl-2-(bromomethyl)-6,8-diphenyl-pyrrolo[2,3-e]indazole **8b**. The compound was prepared in a similar manner to that described for compound **8a** from pyrroloindazole **7b**. White solid. Yield 89 %. Mp. 148-150 °C (hexane/benzene). MS (EI): m/z 622.5. ^{1}H NMR (200 MHz; DMSO-d_6; δ, ppm; *J*, Hz): 1.35 (m, 3H, $CH_2C\underline{H}_3$), 1.59 (s, 3H, 5-$OCOCH_3$), 4.36 (q, 2H, $C\underline{H}_2CH_3$), 4.73 and 4.97 (s, 1H and 1H, CH_2Br), 5.23 (brs, 2H, $C\underline{H}_2$Ph), 6.16-6.35 and 7.03-7.72 (m, 2H and 13H, $CH_2\underline{Ph}$, 6-Ph, 8-Ph), 8.00 (s, 1H, 4-H). Anal. calcd. for $C_{34}H_{28}BrN_3O_4$: C, 65.60; H, 4.53; N, 6.75. Found: C, 63.68; H, 4.51; N, 6.66.

Synthesis of 5-(acetyloxy)-3-ethoxycarbonyl-1-methyl-6,8-diphenyl-2-(piperidin-1-ylmethyl)-pyrrolo[2,3-e]indazole **9a**. To a solution of pyrroloindazole **8a** (1.3 g, 1 eqv.) in benzene (24 mL), piperidine (0.47 mL, 2 eqv.) was added, and then the reaction mixture was stirred at rt for 3 h. The mixture was diluted with water (2×30 mL), the organic phase was separated, dried under anhydrous $MgSO_4$ and concentrated in vacuo. Recrystallization from EtOH gave the product **9a** as a beige solid. Yield 0.98 g (74 %). Mp. 203-205 °C (EtOH). MS (EI): m/z 550.6^{1}H NMR (200 MHz; DMSO-d_6; δ, ppm; *J*, Hz): 1.25-1.50 (m, 9H, $CH_2C\underline{H}_3$, 3'-CH_2, 4'-CH_2, 5'-CH_2), 1.56 (s, 3H, 5-$OCOCH_3$), 2.32 (brs, 4H, 2'-CH_2, 6'-CH_2), 3.34 (s, 3H, 1-CH_3), 3.95 (s, 2H, 2-CH_2), 4.31 (q, 2H, $C\underline{H}_2CH_3$), 7.44-7.76 (m, 10H, 6-Ph, 8-Ph), 7.92 (s, 1H, 4-H). ^{13}C NMR (50 MHz; DMSO-d_6; δ, ppm): 14.45, 19.52, 23.95,

25.70, 35.43, 51.40, 53.60, 59.43, 107.08, 111.77, 114.48, 120.06, 126.86, 128.28, 128.43, 128.84, 128.95, 130.34, 130.61, 131.92, 135.47, 139.58, 141.84, 144.06, 164.52, 168.97. Anal. calcd. for $C_{33}H_{34}N_4O_4$: C, 71.98; H, 6.22; N, 10.17. Found: C, 72.06; H, 6.27; N, 10.13.

5-Acetoxy-3-ethoxycarbonyl-2-((diethylamino)methyl)-1-methyl-6,8-diphenyl-pyrrolo[2,3-e]indazole **9b.** The compound was prepared in a similar manner to that described for compound **9a** from pyrroloindazole **8a** and diethylamine. Light beige solid. Yield 79 %. Mp.185-187 (*i*-PrOH). MS (EI): m/z 538.4. ^{1}H NMR (200 MHz; DMSO-d_6; δ, ppm; *J*, Hz): 0.91 (s, 4H, 2C$\underline{H_2}$CH$_3$), 1.35 (t, 3H, OCH$_2$C$\underline{H_3}$, *J* = 7.0), 1.57 (s, 3H, 5-OCOCH$_3$), 4.09 (brs, 2H, OC$\underline{H_2}$CH$_3$, *J* = 7.0), 4.32 (q, 2H, OC$\underline{H_2}$NEt$_2$), 7.38-7.83 (m, 10H, 6-Ph, 8-Ph), 7.91 (s, 1H, 4-H). ^{13}C NMR (50 MHz; DMSO-d_6; δ, ppm): 11.39, 14.30, 19.43, 35.56, 45.92, 46.87, 59.42, 106.95, 111.60, 114.40, 119.98, 126.80, 128.22, 128.77, 130.16, 130.50, 131.83, 135.34, 139.44, 142.90, 143.95, 164.48, 168.94. Anal. calcd. for $C_{32}H_{34}N_4O_4$: C, 71.35; H, 6.36; N, 10.40. Found: C, 71.41; H, 6.43; N, 10.32.

5-(Acetyloxy)-3-ethoxycarbonyl-1-benzyl-2-(morpholin-4-ylmethyl)-6,8-diphenyl-pyrrolo[2,3-e]indazole **9c.** The compound was prepared in a similar manner to that described for compound **9a** from pyrroloindazole **8b** and morpholine. Light beige solid. Yield 89 %. Mp. 134-136 °C (hexane/benzene). MS (EI): m/z 628.7. ^{1}H NMR (200 MHz; DMSO-d_6; δ, ppm; *J*, Hz): 1.36 (t, 3H, CH$_2$C$\underline{H_3}$, *J* = 7.1), 1.57 (, 3H, 5-OCOCH$_3$), 2.28 (brs, 4H, N(CH$_2$)$_2$O), 2 3.20 (brs, 4H, N(CH$_2$)$_2$O), 3.81 (brs, 2H, 2-CH$_2$), 4.33 (q, 2H, C$\underline{H_2}$CH$_3$, *J* = 7.0), 5.29 (brs, 2H, 1-C$\underline{H_2}$Ph), 6.29 and 6.98-7.74 (d and m, 2H and 13H, 6-Ph, 8-Ph, 1-CH$_2$$\underline{Ph}$, *J* = 3.6), 8.02 (s, 1H, 4-H). ^{13}C NMR (50 MHz; DMSO-d_6; δ, ppm): 14.30, 19.46, 49.57, 50.94, 52.75, 59.66, 65.89, 108.45, 111.45, 114.51, 120.06, 124.79, 126.67, 126.86, 127.04, 127.85, 128.22, 128.40, 128.73, 129.79, 130.94, 132.04, 134.78, 137.61, 139.37, 140.50, 143.68, 164.33, 168.92. Anal. calcd. for $C_{38}H_{36}N_4O_5$: C, 72.59; H, 5.77; N, 8.91. Found: C, 72.67; H, 5.79; N, 8.86.

Synthesis of 5-hydroxy-3-ethoxycarbonyl-1-methyl-6,8-diphenyl-2-(piperidin-1-ylmethyl)-pyrrolo[2,3-e]indazole hydrochloride **10a**. A solution of KOH (0.03 g, 1 eqv.) in EtOH (10 mL) was added to pyrroloindazole **9a** (0.3 g, 1 eqv.), and the reaction mixture was stirred at rt for 10-20 min. The mixture was poured into cold water (10 mL) and acidified by the addition of 33% HCl to pH 6.5. The precipitate was collected and dried. Recrystallization from EtOH gave the product **10a** as a white solid. Yield 0.1 g (64 %). Mp. 285 °C (decomp.). MS (EI): m/z 545.0. ^{1}H NMR (200 MHz; DMSO-d_6; δ, ppm; *J*, Hz): 1.43 (t, 3H, CH$_2$C$\underline{H_3}$, *J* = 7.0), 1.79 (brs, 6H, 3'-CH$_2$, 4'-CH$_2$, 5'-CH$_2$), 3.07 (brs, 2H, 2'-CH$_2$), 3.39 (brs, 5H, 1-CH$_3$, 6'-CH$_2$), 4.39 (q, 2H, C$\underline{H_2}$CH$_3$), 4.74 (brs, 2H, CH$_2$), 7.28-7.84 (m, 11H, 6-Ph, 8-Ph, 4-H), 10.03 (brs, 2H, 5-OH, N$^+$-H). ^{13}C NMR (50 MHz; DMSO-d_6; δ, ppm): 14.33, 21.12, 22.07, 36.33, 49.36, 52.48, 59.86, 103.31, 111.87, 121.25, 126.32, 127.20, 127.85, 128.20, 128.62, 130.00, 130.32, 131.29, 131.77, 135.60, 140.26, 140.71, 144.12, 164.70. Anal. calcd. for $C_{31}H_{33}ClN_4O_3$: C, 68.31; H, 6.10; N, 10.28. Found: C, 68.42; H, 6.26; N, 9.73.

1-Benzyl-5-hydroxy-3-ethoxycarbonyl-2-(morpholin-4-ylmethyl)-6,8-diphenyl-pyrrolo[2,3-e]indazole **10b**.The compound was prepared in a similar manner to that described for compound **10a** from **9b**. The crude product was collected and purified by column chromatography using chloroform as eluent

to afford the product **10b** as a white solid. Yield 0.48 g (63 %). Mp. 145-146 °C. MS (EI): m/z 586.3. ^{1}H NMR (200 MHz; DMSO-d$_6$; δ, ppm; *J*, Hz): 1.38 (t, 3H, CH$_2$C$\underline{H_3}$, *J* = 7.0), 2.28 (brs, 4H, N(CH$_2$)$_2$O), 3.26 (brs, 4H, N(CH$_2$)$_2$O), 3.79 (brs, 2H, CH$_2$), 4.31 (q, 2H, C$\underline{H_2}$CH$_3$, *J* = 7.1), 5.18 (brs, 2H, C$\underline{H_2}$Ph), 6.09-6.41 and 6.95-7.68 (m, 2H and 13H, 6-Ph, 8-Ph, 1-CH$_2$$\underline{Ph}$), 7.76 (s, 1H, 4-H), 9.93 (brs, 1H, 5-OH). ^{13}C NMR (50 MHz; DMSO-d$_6$; δ, ppm): 14.38, 49.35, 51.05, 52.76, 59.30, 65.93, 103.84, 107.52, 111.73, 121.45, 123.01, 124.83, 126.27, 126.52, 127.06, 127.74, 127.83, 128.10, 128.45, 129.71, 130.71, 135.44, 138.08, 139.26, 139.53, 140.73, 143.60, 164.76. Anal. calcd. for $C_{36}H_{34}N_4O_4$: C, 73.70; H, 5.84; N, 9.55. Found: C, 73.84; H, 5.92; N, 9.41.

Synthesis of 5-methoxy-3-ethoxycarbonyl-1,2-dimethyl-8-(4-nitrophenyl)-6-phenyl-pyrrolo[2,3-e]indazole **11a**. To a solution of pyrroloindazole **6e** (0.3 g, 1 eqv.) in *N*-methylpyrrolidone (4 mL), K_2CO_3 (0.18 g, 2 eqv.) and methyl iodide (0.08 mL, 2 eqv.) were added, and the resulting mixture was stirred at rt for 20 h. The reaction mixture was poured into cold aqueous solution of sodium chloride, the precipitate was collected, washed with water and dried. The crude product was purified by column chromatography using hexane/EtOAc mixture as eluent to afford the product **12a** as a beige solid. Yield 0.19 g, (57 %). Mp. 292-295 °C MS (EI): m/z 484.5. ^{1}H NMR (200 MHz; DMSO-d$_6$; δ, ppm; *J*, Hz): 1.38 (t, 3H, CH$_2$C$\underline{H_3}$, *J* = 7.1), 2.66 (s, 3H, 2-CH$_3$), 3.22 (s, 3H, 1-CH$_3$), 3.74 (s, 3H, 5-OCH$_3$), 4.31 (q, 2H, C$\underline{H_2}$CH$_3$), 7.35-7.66 (m, 5H, 6-Ph), 7.71 (s, 1H, 4-H), 7.96 and 8.40 (d, 2H and 2H, 8-C$_6$H$_4$NO$_2$-*p*, *J* = 8.6). ^{13}C NMR (50 MHz; DMSO-d$_6$; δ, ppm): 11.97, 13.82, 14.39, 30.81, 35.82, 55.57, 59.13, 101.29, 104.60, 111.21, 121.49, 123.58, 124.53, 126.28, 127.72, 128.05, 129.10, 130.77, 131.22, 140.83, 141.81, 142.12, 142.72, 147.47, 162.35, 165.00. Anal. calcd. for $C_{27}H_{24}N_4O_5$: C, 66.93; H, 4.99; N, 11.56. Found: C, 66.97; H, 4.95; N, 11.53.

5-Ethoxy-3-ethoxycarbonyl-1-ethyl-2-methyl-8-(4-nitrophenyl)-6-phenyl-pyrrolo[2,3-e]indazole **11b**; *5-ethoxy-3-ethoxycarbonyl-2-methyl-8-(4-nitrophenyl)-6-phenyl-pyrrolo[2,3-e]indazole* **11c**. The compounds were prepared in a similar manner to that described for compound **11a** from pyrroloindazole **6e** and ethyl iodide. The mixture was separated by column chromatography using hexane/EtOAc mixture as eluent to afford the product **11b** as a light beige solid. Yield 10 %. Mp. 175-177 °C. MS (EI): m/z 512.6. ^{1}H NMR (200 MHz; DMSO-d$_6$; δ, ppm; *J*, Hz): 0.72 (t, 3H, CH$_2$C$\underline{H_3}$, *J* = 6.9), 1.05 (t, 3H, CH$_2$C$\underline{H_3}$, *J* = 6.9), 1.37 (t, 3H, CH$_2$C$\underline{H_3}$, *J* = 7.1), 2.65 (s, 3H, 2-CH$_3$), 3.76 (q, 2H, C$\underline{H_2}$CH$_3$), 4.01 (q, 2H, C$\underline{H_2}$CH$_3$), 4.30 (q, 2H, C$\underline{H_2}$CH$_3$), 7.43-7.68 (m, 5H, 6-Ph), 7.73 (s, 1H, 4-H), 7.98 (d, 2H, 8-C$_6$H$_4$NO$_2$-*p*, *J* = 8.6), 8.38 (d, 2H, 8-C$_6$H$_4$NO$_2$-*p*, *J* = 8.6). ^{13}C NMR (50 MHz; DMSO-d$_6$; δ, ppm): 11.43, 13.87, 14.18, 14.60, 58.85, 64.00, 102.18, 105.09, 111.03, 121.00, 121.76, 123.13, 126.50, 127.53, 127.65, 131.32, 140.71, 140.89, 141.32, 141.45, 142.50, 147.56, 164.76. Anal. calcd. for $C_{29}H_{28}N_4O_5$: C, 67.96; H, 5.51; N, 10.93. Found: C, 68.08; H, 5.60; N, 10.89. Compound **11c** – 9 %. Mp. 252-256 °C. MS (EI): m/z 484.5. ^{1}H NMR (500 MHz; DMSO-d$_6$; δ, ppm; *J*, Hz): 1.06 (t, 3H, CH$_2$C$\underline{H_3}$, *J* = 6.9), 1.37 (t, 3H, CH$_2$C$\underline{H_3}$, *J* = 7.1), 2.69 (s, 3H, 2-CH$_3$), 4.01 (q, 2H, C$\underline{H_2}$CH$_3$), 4.31 (q, 2H, C$\underline{H_2}$CH$_3$), 7.33-7.77 (m, 6H, 4-H, 6-Ph), 8.11 (d, 2H, 8-C$_6$H$_4$NO$_2$-*p*, *J* = 8.7), 8.43 (d, 2H, 8-C$_6$H$_4$NO$_2$-*p*, *J* = 8.7), 11.05 (brs, 1H, NH). ^{13}C NMR (126 MHz; DMSO-d$_6$; δ, ppm): 13.80, 14.14, 14.40, 30.78, 35.78, 58.95, 64.13, 102.19, 104.47, 110.44, 119.16, 122.39, 124.51,

126.63, 127.78, 127.91, 129.04, 130.67, 139.75, 140.72, 140.99, 141.59, 141.94, 147.01, 162.32, 165.07. Anal. calcd. for $C_{27}H_{24}N_4O_5$: C, 66.93; H, 4.99; N, 11.56. Found: C, 66.99; H, 5.04; N, 11.59.

3-Ethoxycarbonyl-2-methyl-8-(4-nitrophenyl)-5-(pentyloxy)-6-phenyl-pyrrolo[2,3-e]indazole **11d**. The compound was prepared in a similar manner to that described for compound **11a** from pyrroloindazole **6e** and pentyl iodide. Light beige solid. Yield 19 %. Mp. 177-179 °C. MS (EI): m/z 526.6. ^{1}H NMR (200 MHz; DMSO-d_6; δ, ppm; *J*, Hz): 0.78 (t, 3H, $O(CH_2)_4C\underline{H_3}$, *J* = 7.1), 0.80-1.20 (m, 6H, $OCH_2(C\underline{H_2})_3CH_3$), 1.37 (t, 3H, $CH_2C\underline{H_3}$, *J* = 7.0), 2.68 (s, 3H, 2-CH_3), 3.94 (t, 2H, $OC\underline{H_2}(CH_2)_3CH_3$, *J* = 5.9), 4.29 (q, 2H, $C\underline{H_2}CH_3$), 7.28-7.75 (m, 6H, 4-H, 6-Ph), 8.10 and 8.43 (d, 2H and 2H, 8-$C_6H_4NO_2$-*p*, *J* = 8.7), 11.11 (s, 1H, NH). ^{13}C NMR (50 MHz; DMSO-d_6; δ, ppm): 13.77, 14.36, 21.85, 27.52, 28.25, 58.91, 68.16, 69.78, 101.41, 104.45, 110.33, 118.97, 122.37, 124.47, 126.64, 127.91, 128.98, 130.68, 139.74, 140.86, 141.23, 141.50, 141.80, 146.96, 165.03. Anal. calcd. for $C_{30}H_{30}N_4O_5$: C, 68.43; H, 5.74; N, 10.64. Found: C, 68.51; H, 5.83; N, 10.56.

Synthesis of 5-(acetyloxy)-3-ethoxycarbonyl-2-methyl-8-(4-nitrophenyl)-6-phenyl-pyrrolo[2,3-e]indazole **11e**. The compound was prepared in a similar manner to that described for compound **7a** from pyrroloindazole **6e**. Yield 55 %. Mp. 223-225 °C (AcOH). MS (EI): m/z 498.5. ^{1}H NMR (200 MHz; DMSO-d_6; δ, ppm; *J*, Hz): 1.39 (t, 3H, $CH_2C\underline{H_3}$, *J* = 7.2), 1.61 (s, 3H, 5-$OCOCH_3$), 2.72 (s, 3H, 2-CH_3), 4.31 (q, 2H, $C\underline{H_2}CH_3$), 7.53-7.68 (m, 5H, 6-Ph), 7.85 (s, 1H, 4-H), 8.10 (d, 2H, 8-$C_6H_4NO_2$-*p*, *J* = 8.0), 8.44 (d, 2H, 8-$C_6H_4NO_2$-*p*, *J* = 8.0), 11.37 (s, 1H, NH). ^{13}C NMR (50 MHz; DMSO-d_6; δ, ppm): 13.75, 14.45, 19.55, 59.17, 104.81, 110.36, 114.49, 121.55, 122.82, 124.55, 126.73, 128.61, 128.90, 129.25, 130.40, 131.63, 139.30, 139.41, 141.95 143.23, 147.26, 164.76, 169.00. Anal. calcd. for $C_{27}H_{22}N_4O_6$: C, 65.06; H, 4.45; N, 11.24. Found: C, 65.15; H, 4.53; N, 11.17.

Synthesis of 8-(4-aminophenyl)-5-hydroxy-3-ethoxycarbonyl-2-methyl-6-phenyl-pyrrolo[2,3-e]indazole **12**. A mixture of pyrroloindazole **6e** (0.44 g, 1 eqv) and Pd/C (10 wt % on activated carbon, 0.05 g, 10 mol %) in EtOH (100 mL) was stirred under an atmosphere of H_2 (4 bar) at rt for 24 h. The reaction mixture was filtered over silica gel, washed with dichloroethane and concentrated in vacuo. The residue was purified by column chromatography using chloroform/methanol mixture as eluent to afford the product **13** as a white solid. Yield 0.24 g (59 %). Mp. 293-295 °C. MS (EI): m/z 426.5. ^{1}H NMR (200 MHz; DMSO-d_6; δ, ppm; *J*, Hz): 1.37 (t, 3H, $CH_2C\underline{H_3}$, *J* = 7.2), 2.68 (s, 3H, 2-CH_3), 4.30 (q, 2H, $C\underline{H_2}CH_3$), 5.35 (s, 2H, NH_2), 6.74 (d, 2H, 8-$C_6\underline{H_4}NH_2$-p, *J* = 8.0), 7.30-7.66 (m, 8H, 4-H, 6-Ph, 8-$C_6\underline{H_4}NH_2$-p), 9.64 (s, 1H, 5-OH), 10.71 (s, 1H, NH). ^{13}C NMR (50 MHz; DMSO-d_6; δ, ppm): 13.82, 14.55, 58.72, 103.76, 110.61, 114.40, 119.35, 120.67, 121.85, 125.98, 126.51, 127.78, 128.63, 129.73, 138.93, 141.11, 141.29, 144.74, 148.90, 165.28. Anal. calcd. for $C_{25}H_{22}N_4O_3$: C, 70.41; H, 5.20; N, 13.14. Found: C, 70.50; H, 5.26; N, 13.19. IR (KBr, ν/cm^{-1}): 3450 (OH), 3387 and 3363 (NH_2), 3221 (NH), 1657 (C=O), 1497, 1454, 1175, 1130.

Synthesis od 5-hydroxy-3-ethoxycarbonyl-2-methyl-6-phenyl-8-[4-(acetylamino)phenyl]-pyrrolo[2,3-e]indazole **13**. The compound was prepared in a similar manner to that described for compound **7a** from pyrroloindazole **12**. The residue was purified by column chromatography using hexane/EtOAc mixture as eluent to afford the product **14** as a white solid. Yield 0.04 g, 57 %. Mp.

291-294 °C. MS (EI): m/z 468.5. ^{1}H NMR (200 MHz; DMSO-d_6; δ, ppm; *J*, Hz): 1.38 (t, 3H, $CH_2C\underline{H}_3$, *J* = 7.2), 2.14 (s, 3H, 8-C_6H_4(NH-COC$\underline{H}_3$-p)), 2.68 (s, 3H, 2-CH_3), 4.29 (q, 2H, $C\underline{H}_2CH_3$), 7.31-7.88 (m, 10H, 4-H, 6-Ph, 8-$C_6\underline{H}_4$NHAc-p), 9.69 (1H, s, 5-OH), 10.15 (1H, s, -N$\underline{H}$-$COCH_3$), 10.85 (s, 1H, NH). ^{13}C NMR (50 MHz; DMSO-d_6; δ, ppm): 13.83, 14.55, 24.09, 58.73, 103.80, 119.04, 119.45, 122.16, 126.11, 126.83, 127.84, 127.98, 128.28, 129.86, 138.96, 139.41, 140.97, 141.50, 143.65, 165.24, 168.43. Anal. calcd. for $C_{27}H_{24}N_4O_4$: C, 69.22; H, 5.16; N, 11.96. Found: C, 69.31; H, 5.24; N, 11.87.

Synthesis of 5-hydroxy-3-ethoxycarbonyl-2-methyl-6-phenyl-8-[4-(1H-pyrrol-1-yl)phenyl]-pyrrolo[2,3-e]indazole **14**. To a suspension of pyrroloindazole **12** (0.15 g, 1.0 eqv.) in AcOH (4mL), 2,5-dimethoxytetrahydrofuran (0.05 mL, 1.1 eqv.) was added, and then the resulting mixture was stirred at 50 ºC for 45 min. The solvent was concentrated in vacuo, and the residue was purified by column chromatography using hexane/EtOAc mixture as eluent to afford the product **15** as a white solid. Yield 0.10 g (59 %). Mp. 270-272 °C. MS (EI): m/z 476.5. ^{1}H NMR (200 MHz; DMSO-d_6; δ, ppm; *J*, Hz): 1.39 (t, 3H, $CH_2C\underline{H}_3$, *J* = 7.2), 2.59 (s, 3H, 2-CH_3), 4.29 (q, 2H, $C\underline{H}_2CH_3$), 6.32 (d, 2H, Ar, *J* = 8.00), 7.30-7.93 (m, 12H, 4-H, 6-Ph, 8-C_6H_4, Ar), 9.70 (s, 1H, 5-OH), 10.92 (s, 1H, NH). ^{13}C NMR (50 MHz; DMSO-d_6; δ, ppm): 13.76, 14.48, 58.70, 103.87, 104.05, 110.67, 118.83, 118.91, 119.76, 122.26, 126.09, 126.86, 127.81, 129.08, 129.92, 130.16, 138.95, 139.62, 140.90, 141.47, 143.05, 165.15. Anal. calcd. for $C_{29}H_{24}N_4O_3$: C, 73.09; H, 5.08; N, 11.76. Found: C, 73.14; H, 5.19; N, 11.71.

X-Ray Diffraction Analysis

Single-Crystal Diffraction

The molecular structure of compound **6a** was confirmed by X-ray structure determination technique on a STOE STADI P X-ray diffractometer (STOE & Cie, Darmstadt, Germany) equipped with a Pilatus100K detector (DECTRIS, Baden, Switzerland), focusing mirror collimation Cu K_α (1.54086Å) radiation in rotation method mode. Data collection and image processing was performed using STOE X-Area 1.67 software (STOE & Cie). Intensity data were scaled using STOE LANA software (STOE & Cie) to minimize the difference in intensity of symmetry-equivalent reflections (multiscan method). The structure of compound **6a** was solved and refined using the SHELX crystallographic suite.[3] The non-hydrogen atoms were refined using an anisotropic full-matrix least-square procedure. All hydrogen atoms were placed in the calculated positions and allowed to ride on their parent atoms [C-H 0.93-0.98; Uiso 1.2 Ueq(parent atom)]. The position of the hydrogen atom of the hydroxy group was found from the Fourier synthesis and is refined freely. The crystal data are given in Table S1.

Powder Diffraction

The molecular structure of compound **12** was confirmed by X-ray structure determination technique using powder measurements at room temperature on a EMPYREAN laboratory X-ray diffractometer (Malvern Panalytical, Malvern, United Kingdom) (Ni-filtered Cu K_α radiation) equipped

with a X'celerator linear detector (Malvern Panalytical). The powder pattern was indexed in the triclinic unit cell. The crystal structure was solved in the *P-1* space group using the simulated annealing method[4] and refined using MRIA software[5] according to the procedure described earlier.[6,7] In the final bond-restrained Rietveld refinement, the severe texture was accounted for using symmetrized harmonics expansion up to the fourth order.[8,9] The experimental and calculated diffraction patterns after final refinement are shown in Fig. S3. The crystal data are given in Table S1. The figures were prepared with Mercury 4.2.0. tool (Cambridge Crystallographic Data Centre, www.ccdc.cam.ac.uk/solutions/software/free-mercury/).

The crystallographic data for compound **6a** and compound **12** were deposited with the Cambridge Crystallographic Data Centre (CCDC ID 2246679 and 2249815, respectively).

Biological Assays

Cells and viruses

Madin Darby canine Kidney (MDCK; Friedrich Löffler Institute, Greifswald, Germany) were propagated in Eagle´s minimal essential medium with 10% fetal calf serum, 2 mM L-glutamine, and 1 % non-essential amino acids. Antiviral tests with influenza A viruses were performed in MDCK cells with Eagle´s minimal essential medium supplemented with 2.3 % sodium bicarbonate, 2 µg/mL trypsin, 2 mM L-glutamine, and 1% non-essential amino acids (test medium).

H3N2 influenza virus A/Hong Kong/1/68 (Schaper and Brümmer, Salzgitter, Germany) and the A(H1N1)pdm09 influenza virus A/Jena/8178/09 (isolated and kindly provided by Andy Krumbholz[10] were grown and titrated (2.0×10^7 $TCID_{50}$/mL and 6.3×10^7 $TCID_{50}$/mL, respectively) in MDCK cells. Aliquots of the viruses were stored at -80 °C until use.

Human erythrocytes, obtained from the Institute of Transfusion Medicine, University Hospital Jena (Jena, Germany) were stored at 4 °C. Blood cells were washed three times in PBS without Ca^{2+} and without Mg^{2+} before using.

Cytotoxicity measurement

MDCK cells were seeded at 2.3×10^4 cells/well in 100 µL growth medium in 96-well flat-bottomed microtiter plates and grown in a 5% CO_2 atmosphere at 37°C for two days. After removal of the growth medium from the confluent cell monolayers, 100 µL test medium (cell control; n = 6) or 50 µL of test medium and six half-log dilutions of test compounds in test medium (each concentration in duplicates; maximum 100 µM) were added. After 72 h of incubation, the tests were stained, measured, and analyzed as described previously.[11] Cell viability in a well was defined as the percentage of the mean value of optical density resulting from the 6 cell controls, which was set 100 % cell viability. The inhibitor concentration that reduces the mean value of optical density determined for cell controls by half is called 50 % cytotoxic concentration (CC_{50}). A minimum of three independent experiments were performed.

Determination of influenza A virus-induced CPE inhibition

We analyzed the ability of the compounds to protect cells from virus-induced cytopathic effect (CPE) as published previously.[11] The anti-influenza drugs targeting the viral neuraminidase, oseltamivir carboxylate (Roche AG, Basel, Switzerland), zanamivir (GlaxoSmithKline, London, UK), served as reference compounds. Stock solutions (10,000 µM) of oseltamivir or zanamivir and test compounds were prepared in bi-distilled water and DMSO, respectively, and working solutions in the test medium. Seeding and growth of MDCK cells was done similarly to the cytotoxicity assay. CPE inhibitory assays started with a washing step with 100 µL test medium which was afterwards replaced by 50 µL of test medium (mock-treatment of cell and virus controls; each $n = 6$ per plate) or 6 half-log dilutions of oseltamivir, or zanamivir, or test compounds (each concentration in duplicates; maximum 100 µM) in test medium. Then we inoculated 50 µL test medium (mock-infection of cell controls) or a virus suspension consisting a certain multiplicity of infection (MOI) in 50 µL of the test medium to the cell monolayers. The MOI of influenza virus A/Hong Kong/68 and influenza virus A/Jena/8178/09 was 0.008, and 0.005 $TCID_{50}$/cell, respectively. After 48 h of incubation, fixation, staining, and analyses were performed as described previously.[11] The inhibitor concentration reducing the virus-induced cytopathic effect (0% cell viability) by 50% (50% viability of cell control) is called 50 % cytopathic effect inhibitory concentration (IC_{50}). A minimum of three independent experiments were performed.

Inhibition of viral and pneumococcal neuraminidases

Inhibition of viral neuraminidase: We used a published protocol to evaluate the effect of test compounds on neuraminidase activity[12] with influenza virus A/Jena/5258/09 (4 hemagglutination units) and a 1% human erythrocytes solution. The potential of serial half-logarithmic dilutions of oseltamivir or zanamivir (control; maximum test concentration: 1 µM) or test compounds (duplicates; maximum test concentration: 100 µM) to inhibit the viral hemagglutinin-induced agglutination of erythrocytes was evaluated at 4 °C after a two-hour incubation. Thereafter, we incubated the assays at 37 °C for further 24 h allowing viral neuraminidase activation. The activated viral neuraminidase abrogates the virus-mediated hemagglutination in absence of inhibitors (hemagglutination control) or in the presence of ineffective inhibitor concentrations. Neuraminidase inhibitors prevent this abrogation of virus-mediated hemagglutination. We recorded the lowest compound concentration that completely inhibits the abrogation of virus-mediated hemagglutination by the viral neuraminidase activity as the minimum neuraminidase inhibitory concentration and repeated the assay once for result confirmation.

Inhibition of pneumococcal neuraminidases: Ethanol-precipitated proteins of *Streptococcus pneumoniae* strain DSM20566 (German Collection of Microorganisms and Cell Cultures GmbH, Braunschweig, Germany) in phosphate-buffered saline (PBS) were applied to test the inhibitory activity of compounds against bacterial neuraminidase activity with lectin-based hemagglutination HA assay as described previously.[12] A cocktail of erythrocytes, lectin, and compounds without bacterial protein (one well per compound concentration) as well as erythrocytes and compounds alone was included to confirm that hemagglutination was not caused by the interaction between

compounds and erythrocytes. We recorded the lowest compound concentration that completely inhibits the hemagglutination as the minimum neuraminidase inhibitory concentration and repeated the assay once for result confirmation. Each active compound was tested at least three times, inactive compounds two times.

Antibacterial assays

We studied the inhibition of planktonic growth and biofilm formation of *S. pneumoniae DSM20566* (DSM20566; serotype 1; ATCC 33400, Leibniz Institute DSMZ-German Collection of Microorganisms and Cell Cultures, Heidelberg, Germany) by the test compounds as published.[13] Briefly, DSM20566 was grown on Columbia blood agar plates supplemented with 5% sheep blood (Becton Dickinson GmbH, Heidelberg, Germany) in a 5% CO_2 atmosphere at 37 °C overnight. Afterwards, bacteria were grown in brain heart infusion (BHI) broth. For determination of bacterial growth and biofilm inhibition, samples of those precultured pneumococci were diluted in BHI to match the turbidity of 1.5×10^8 colony forming units (CFU) mL^{-1} (equivalent to a McFarland standard of 0.5).

To study the inhibition of planktonic growth in microtiter broth microdilution assay, untreated pneumococci in BHI (untreated control) or pneumococci with serial compound dilutions (dilution factor 2; maximum tested concentration 50 µM) were incubated in 96-well V-shape plates (Greiner bio-one GmbH, Kremsmünster, Austria) in a 5% CO_2 atmosphere at 37°C for 18 h. The planktonic growth of untreated and compound-treated pneumococci was compared by measuring optical density (OD) at 620 nm. The inhibitor concentration reducing the mean OD of six untreated controls (set 100% planktonic growth) by 50% is called inhibitory concentration (IC_{50}).

For the biofilm assay, broth was diluted 50-fold in tryptic soy broth and incubated in 96-well flat-bottom plates in a 5% CO_2 atmosphere at 37 °C for 2 h. Then, the supernatant was replaced by 200 µL of fresh medium without or with compound (maximum 50 µM; each concentration in duplicate). After 24 h incubation, plates were washed with water and biofilms stained with crystal violet overnight. After rinsing the plates with water, the crystal violet was eluted with lysis buffer, and the optical density of the elution was measured at 550 nm. The 50% biofilm inhibitory concentration (BIC_{50}) was defined as the drug concentration reducing the optical density of six untreated controls (set 100% growth of biofilm) by 50%.

Data analysis

The 50% cytotoxic and inhibitory concentrations (CC_{50} and IC_{50} values) were calculated from dose-response curves by using a Four Parameter Logistic (4PL) Curve Calculator (AAT Bioquest Inc, Pleasanton, CA USA). The means were calculated using Microsoft Excel 2010.

Molecular Dynamics Simulations

The source of the coordinates for influenza A H1N1 neuraminidase was the crystal structure PDB: 4B7Q[14]; 3D models for neuraminidases NanA and NanB from the *Streptococcus pneumonia* were prepared from the coordinates of heavy atoms PDB: 2VVZ[15] and 2JKB[16], respectively. Preliminary molecular docking of compound **12** was performed using the Autodock4 tool.[17] The set

of the top 8 complexes was utilized as initial states for subsequent molecular dynamics simulations for each system. Molecular dynamics simulations were performed for 100 ns for each model in 1 fs integration time steps using NAMD 3.0 Alpha MD software.[18] Additional production runs of 150 ns were performed for the stable complexes that differed from the docking predicted ones but formed during the MD simulations. The protein macromolecule was described by CHARMM36 force field parameters,[19,20] compound **12** by CGenFF[21] and solvent water molecules by TIP3P[22] parameters. Two-dimensional maps of the binding sites were partly composed using the PoseView tool.[23,24] Three-dimensional models were analyzed in the VMD software.[25]

X-Ray Diffraction Analysis Results

Table S1. Crystallographic data for compounds **6a** and **12**

Parameters	Compound 6a	Compound 12
**CCDC number**	2246679	2249815
**Empirical formula**	$C_{25}H_{21}N_3O_3$	$C_{25}H_{22}N_4O_3$
**Formula weight**	411.45	426.47
**Temperature,** K	295(2)	295(2)
**Wavelength,** Å	1.54186	1.54180
**Sample**	Single-crystal	Powder
**Crystal system**	Triclinic	Triclinic
**Space group**	P-1	P-1
**Unit cell dimensions**		
**a, Å**	9.4900(10)	9.7488(12)
**b, Å**	10.2300(10)	10.0324(15)
**c, Å**	12.327(2)	12.6387(18)
**α, °**	97.010(10)	96.046(17)
**β, °**	105.660(10)	105.086(19)
**γ, °**	112.190(10)	112.80(2)
**Volume, $Å^3$**	1032.7(2)	1070.3(3)
**Z**	2	2
**Density (calculated), Mg/m^3**	1.323	1.323
**Absorption coefficient μ, mm^{-1}**	0.715	0.722
**F(000)**	433	446.0
**Crystal size, mm^3**	0.17 x 0.011 x 0.04	
**Θ_{min} - Θ_{max}, °**	3.848 - 66.963	
**$2\Theta_{min}$ - $2\Theta_{max}$, $\Delta 2\Theta$, °**		5.000 – 75.006, 0.017
**Index ranges**	-11<=h<=11, -12<=k<=12, -13<=l<=14	
**Reflections collected**	11960	
**Completeness to theta = 66.963°, %**	99.0	
**Refinement method**	Full-matrix least-squares on F^2	Rietveld refinement
**Data / restraints / parameters**	11960 / 0 / 292	4119 / 122/ 179
**Goodness-of-fit**	0.738	2.471
**Final R indices [I>2sigma(I)]**	R_1 = 0.0887, wR_2 = 0.1947	

**R indices (all data)**	R_1 = 0.2301, wR_2 = 0.2523	
**Profile R indices**		R_p = 0.0407, R_{wp} = 0.0577, R_{exp} = 0.0234
**Extinction coefficient**	0.0046(11)	
**Largest diff. peak and hole, eÅ^{-3}**	0.332 and -0.343.	0.499 and -0.527

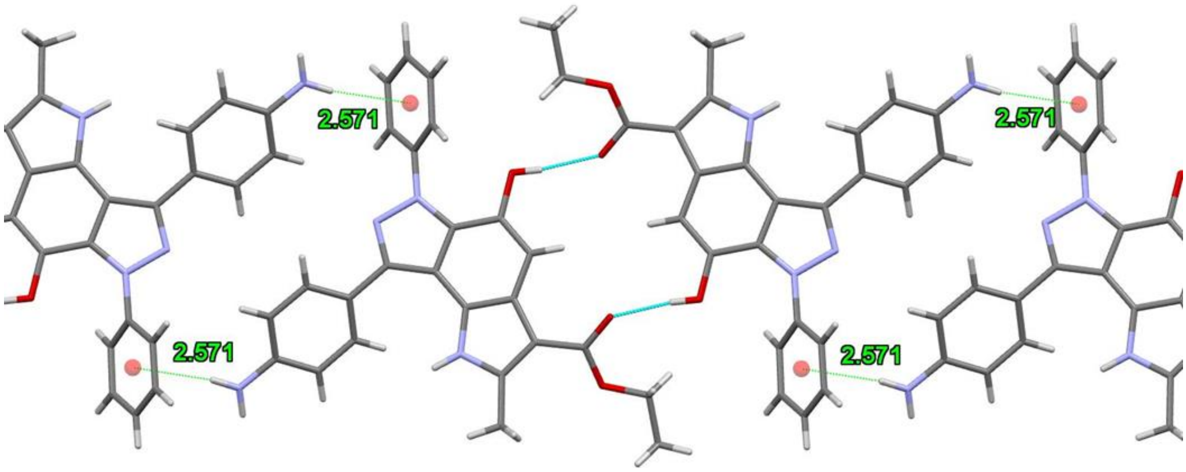

Figure S1. Part of the crystal packing in compound **12**. The red balls denote the centroids (Cg) of the C14-C19 benzene rings, with a N4…Cg distance of 3.425(17) Å. The thin cyan lines indicate hydrogen bonds that bind the molecules into centrosymmetric dimers. The dotted green lines denote the weak N---H…π interactions, with a H4B…Cg distance of 2.57 Å, which bind the dimers into ribbon-like stretches

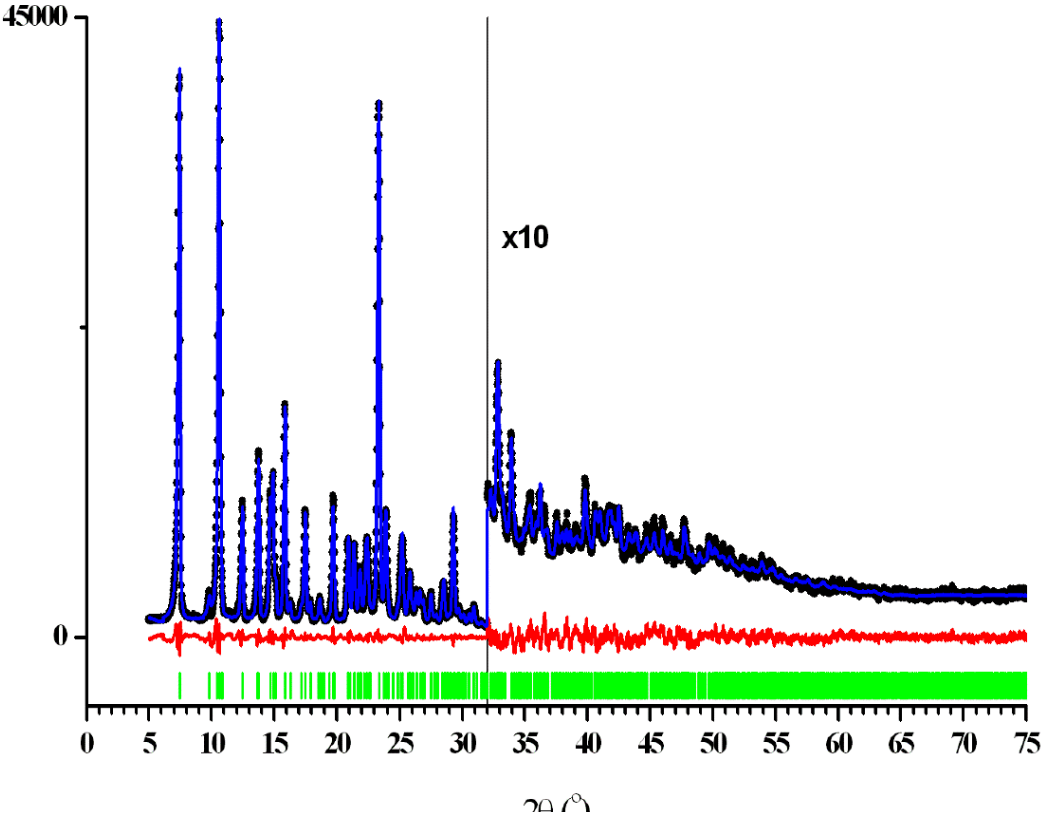

Figure S2. Experimental (black line), calculated (blue line), and difference (red line) powder X-ray diffraction patterns of compound **12**. The green vertical bars indicate the calculated positions of the Bragg peaks

Predicted Binding Mode of Compound 12 to *Streptococcus pneumoniae* NanB

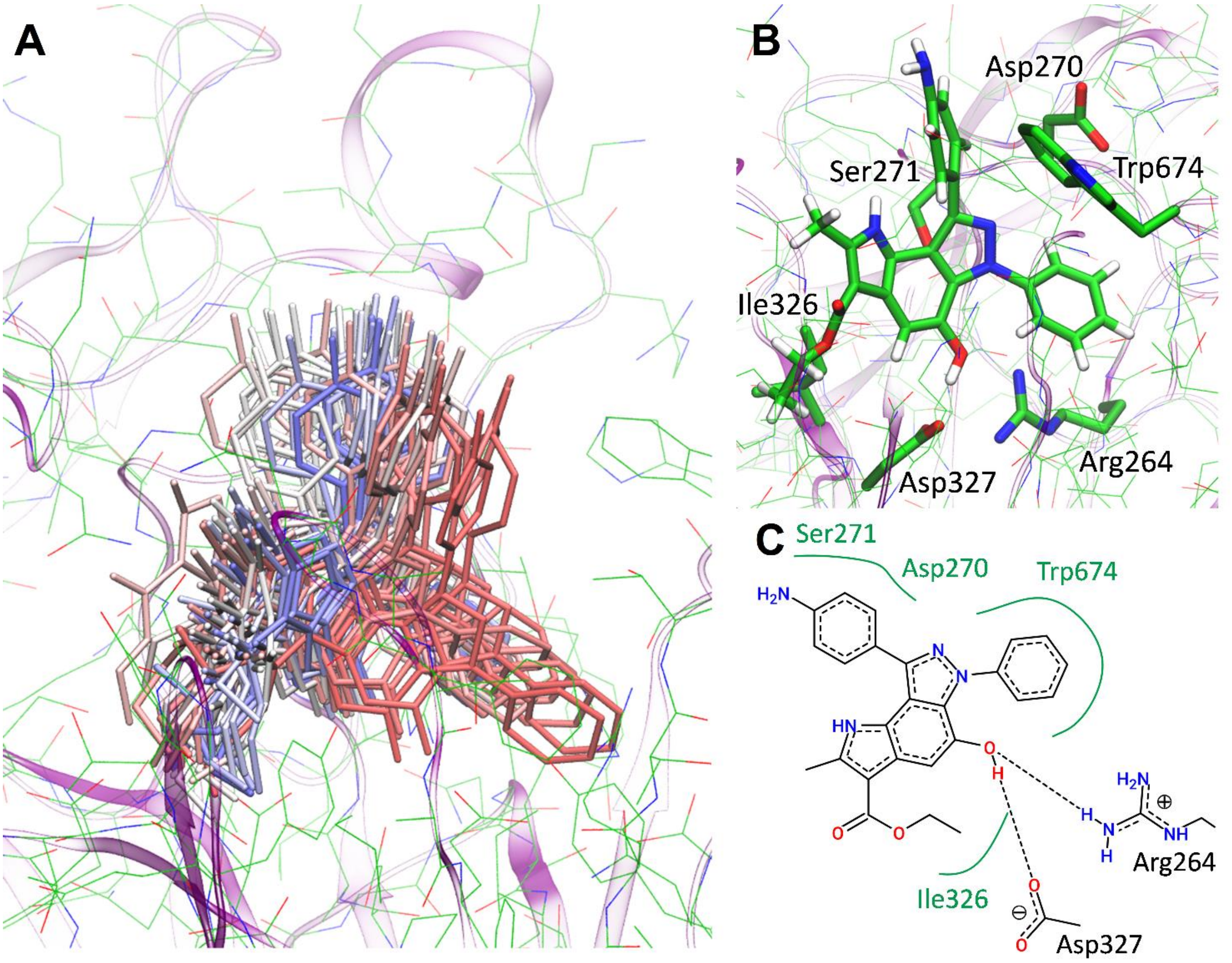

Figure S3. **A**, Complex stability at a 100 ns MD trajectory. MD frames are aligned over the protein backbone, and compound **12** is shown in sticks. **B**, **C**: Predicted binding modes (**B**, 3D representation; **C**, 2D representation) for compound **12** bound to *Streptococcus pneumoniae* NanB. In the 2D model hydrogen bonds are shown as dashed lines and hydrophobic interactions as green curves. Color code: carbon – green, nitrogen – blue, oxygen – red, hydrogen – white. Hydrogen atoms are shown only for compound **12**.

^{1}H and ^{13}C Spectra of Final Pyrrolo[2,3-*e*]indazoles

^{1}H NMR spectrum (200 MHz, DMSO-d_6) of compound **6a**

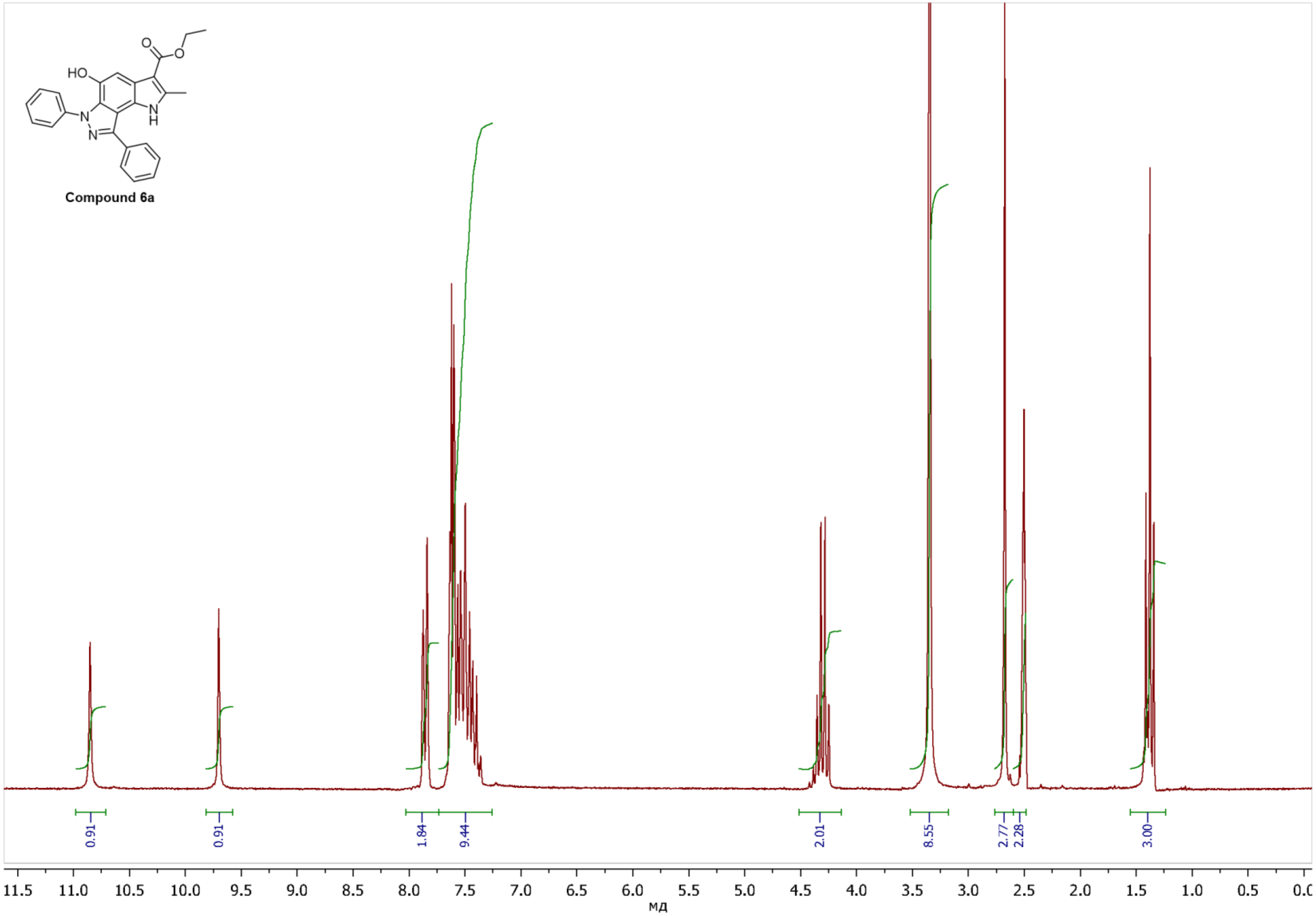

^{13}C NMR spectrum (50 MHz, DMSO-d$_6$) of compound **6a**

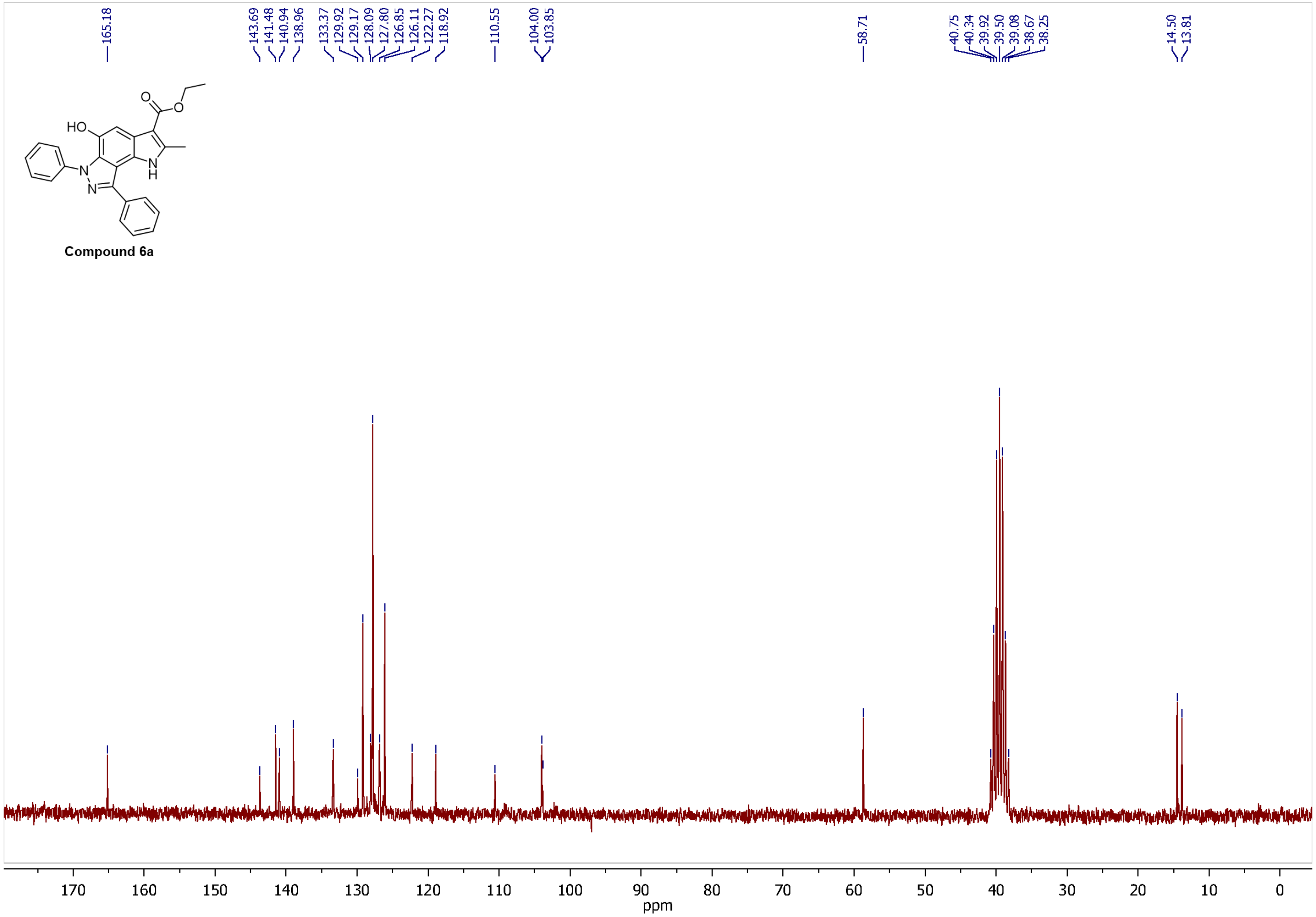

^{1}H NMR spectrum (200 MHz, DMSO-d_6) of compound **6b**

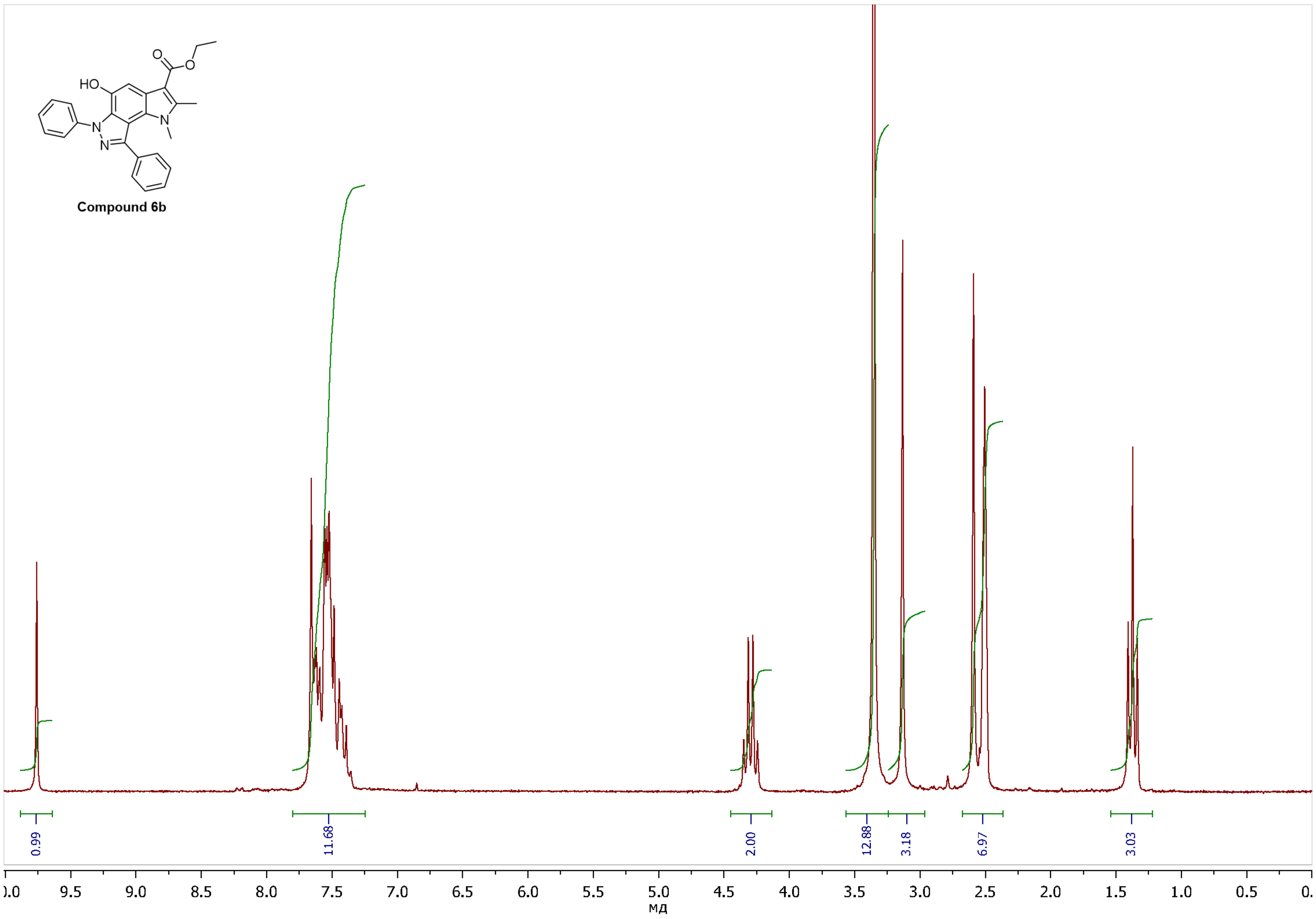

^{13}C NMR spectrum (50 MHz, DMSO-d_6) of compound **6b**

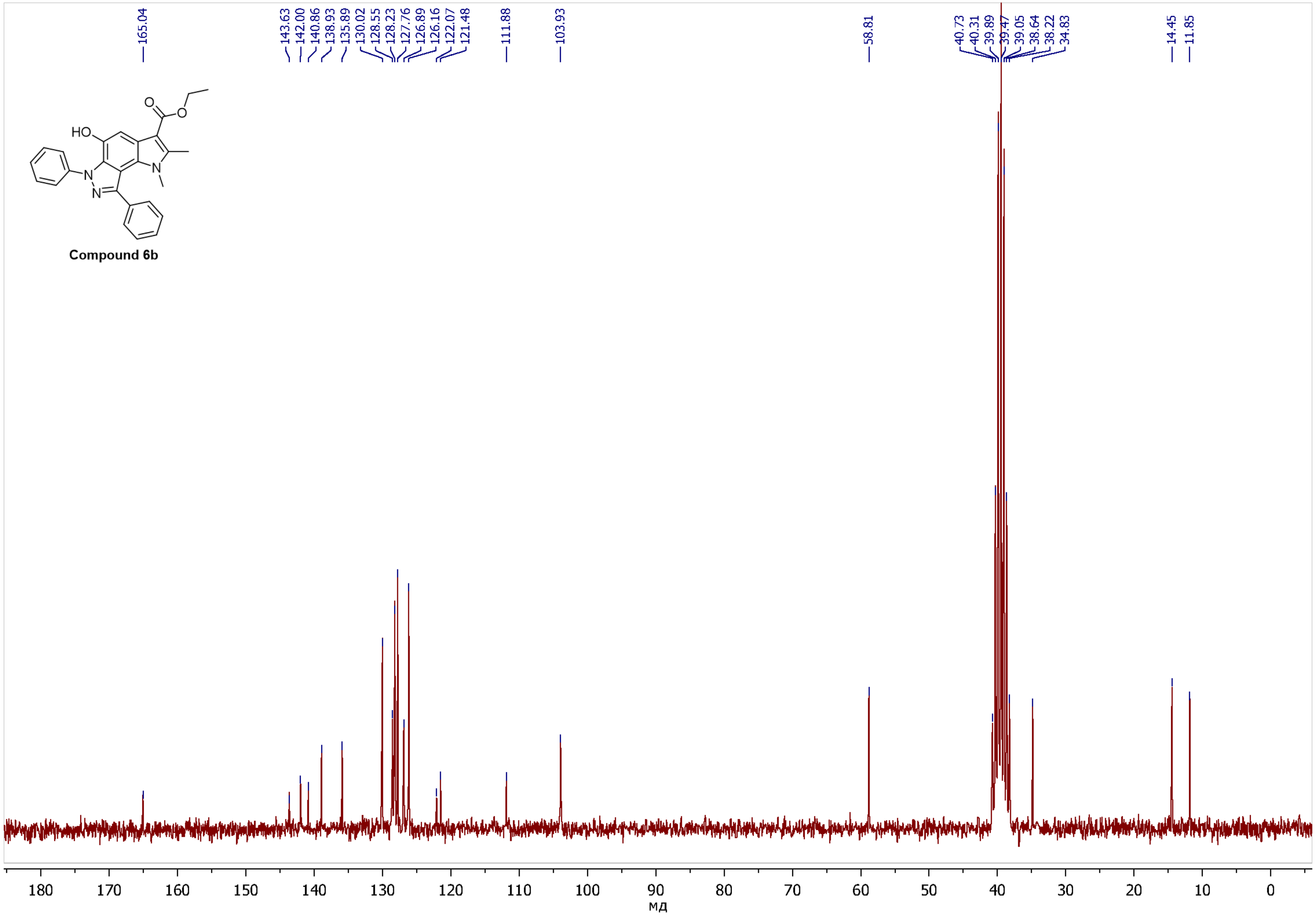

^{1}H NMR spectrum (200 MHz, DMSO-d_6) of compound **6c**

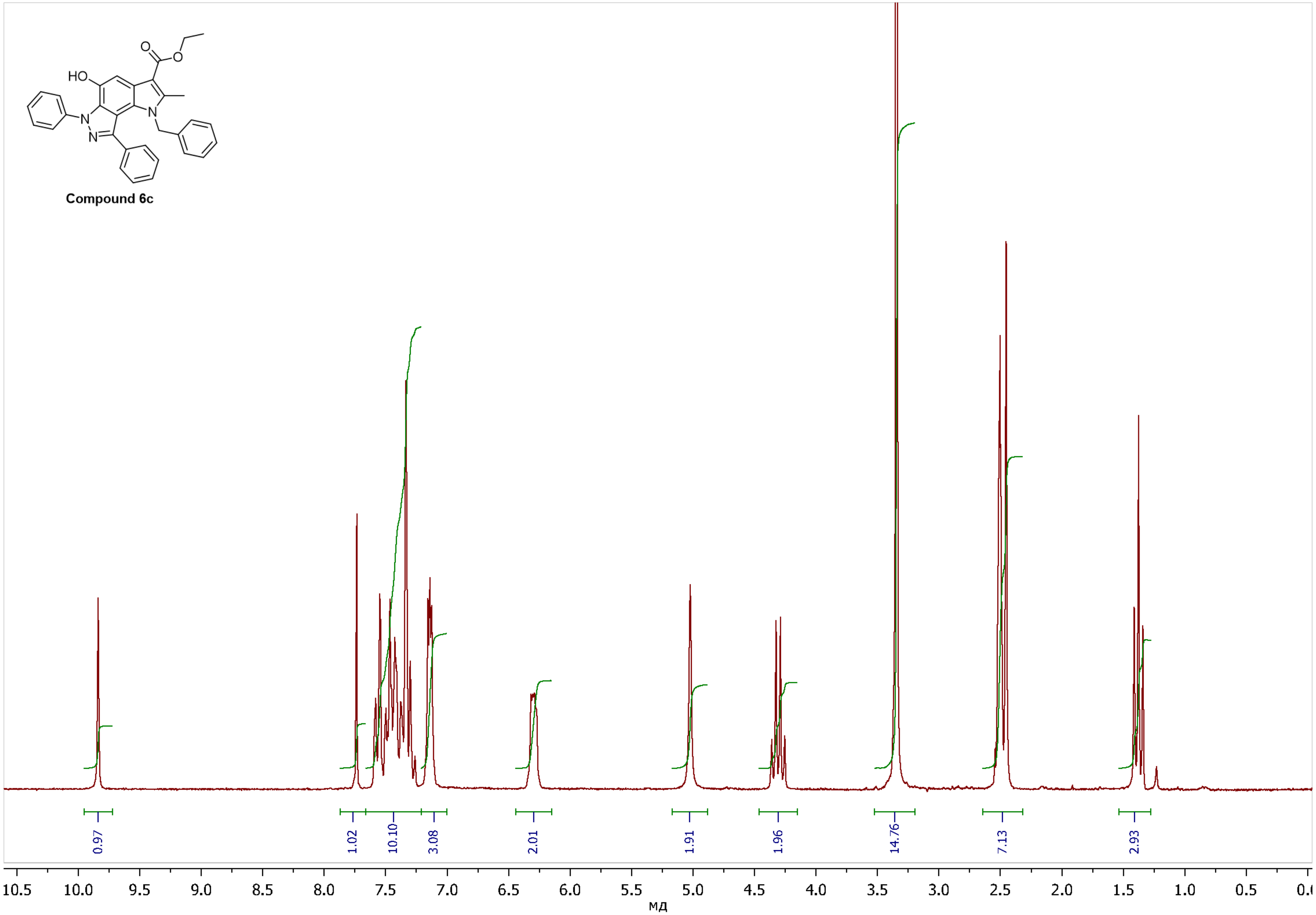

^{13}C NMR spectrum (50 MHz, DMSO-d_6) of compound **6c**

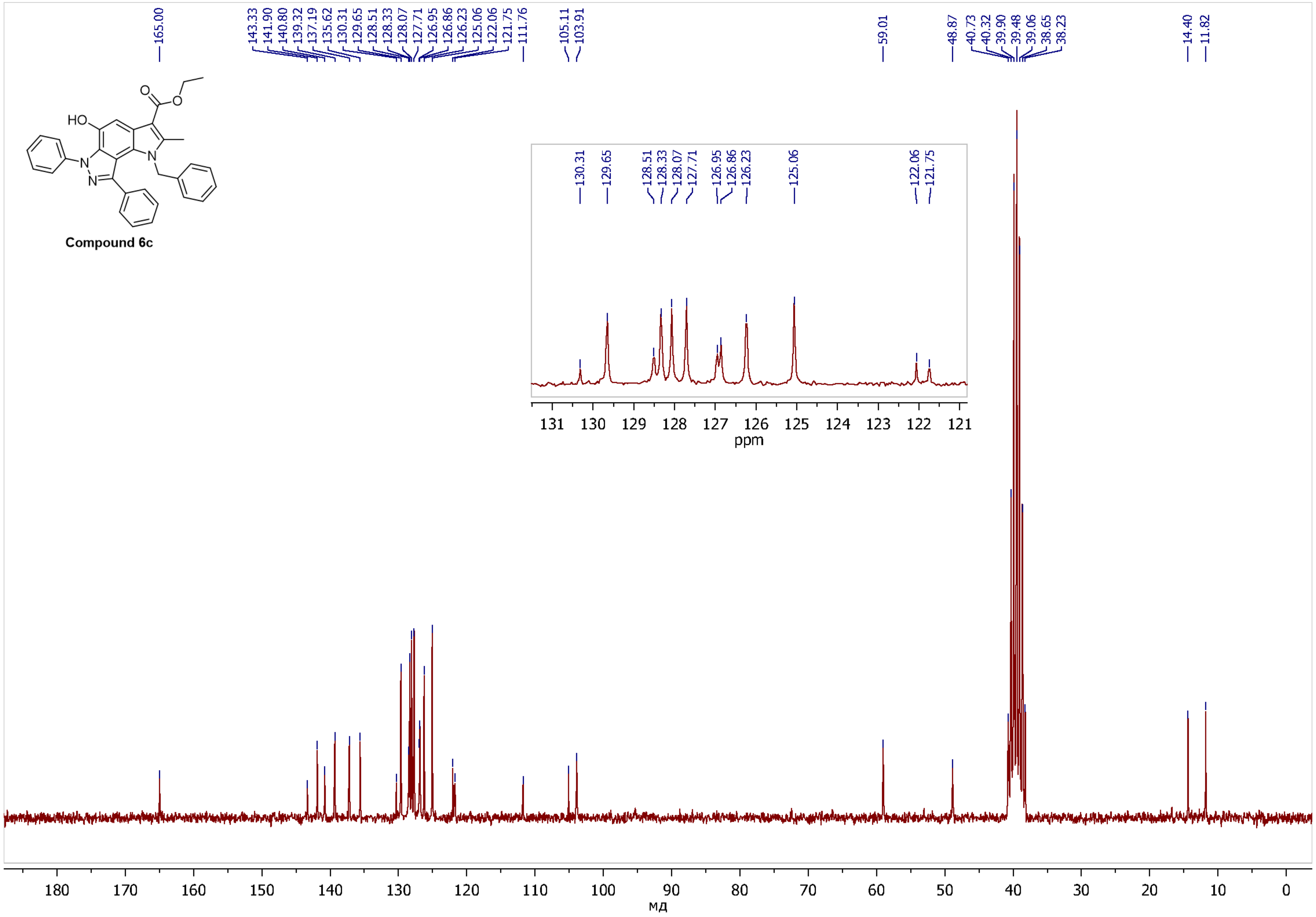

^{1}H NMR spectrum (200 MHz, DMSO-d_6) of compound **6d**

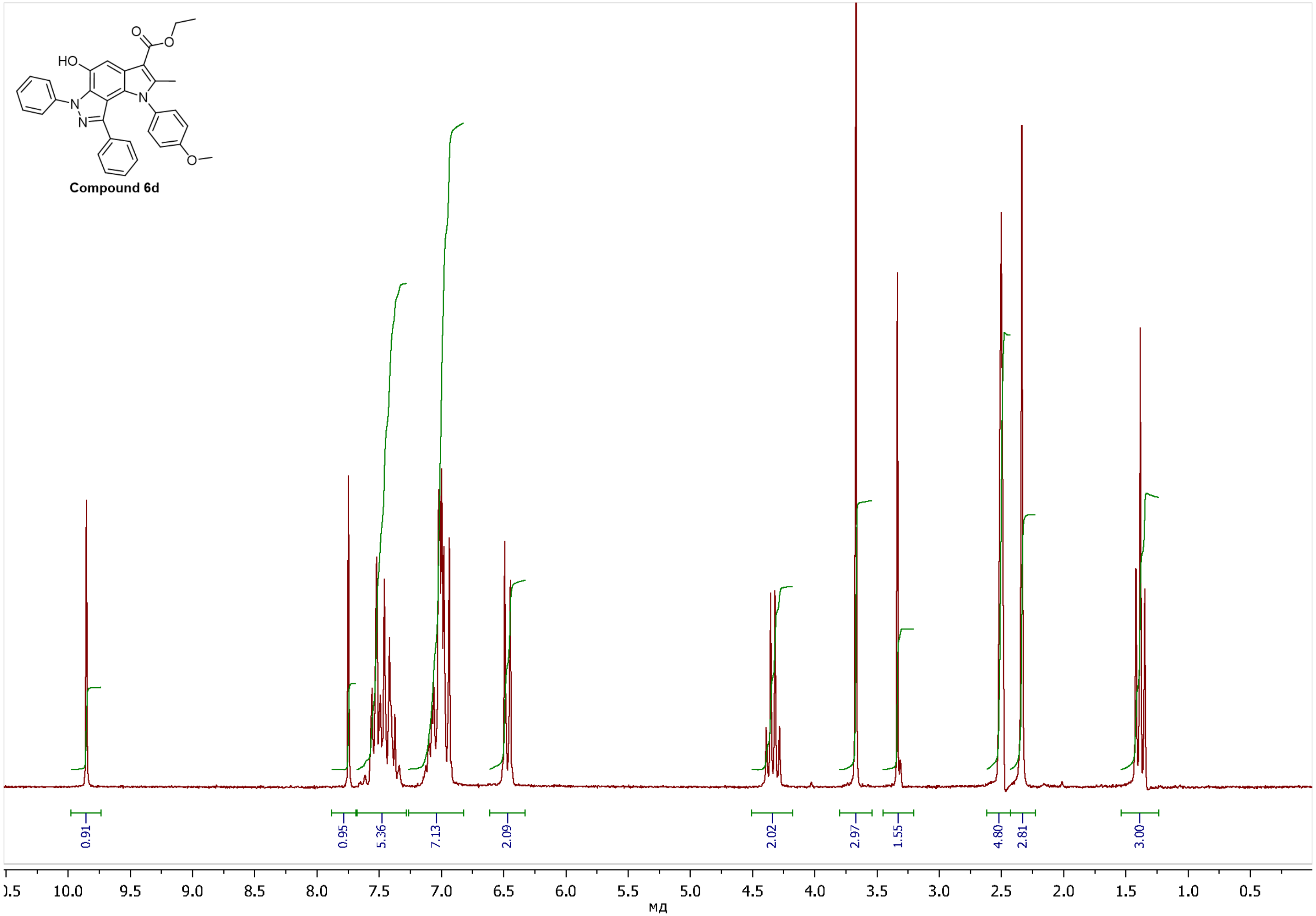

^{13}C NMR spectrum (50 MHz, DMSO-d_6) of compound **6d**

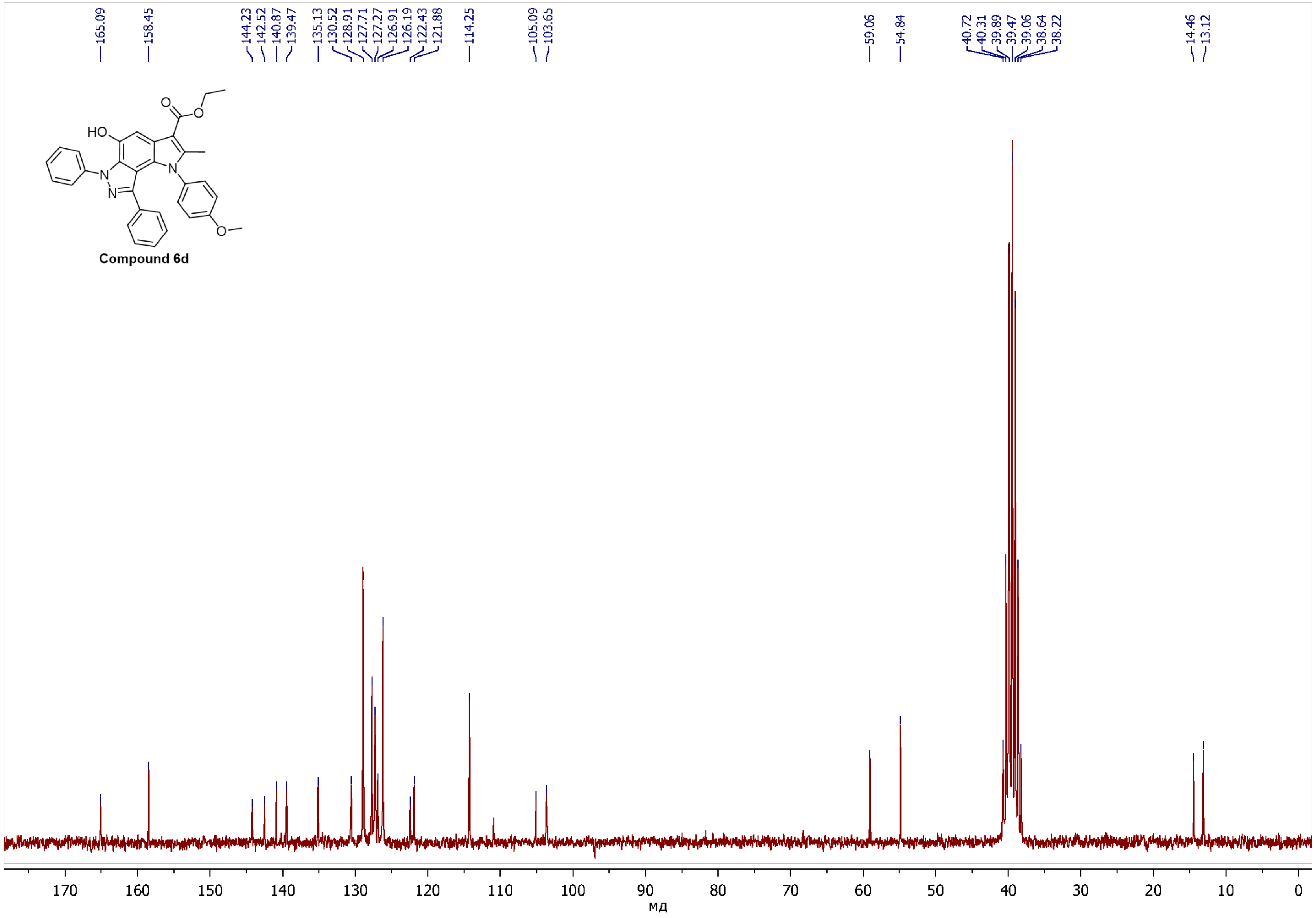

^{1}H NMR spectrum (200 MHz, DMSO-d$_6$) of compound **6e**

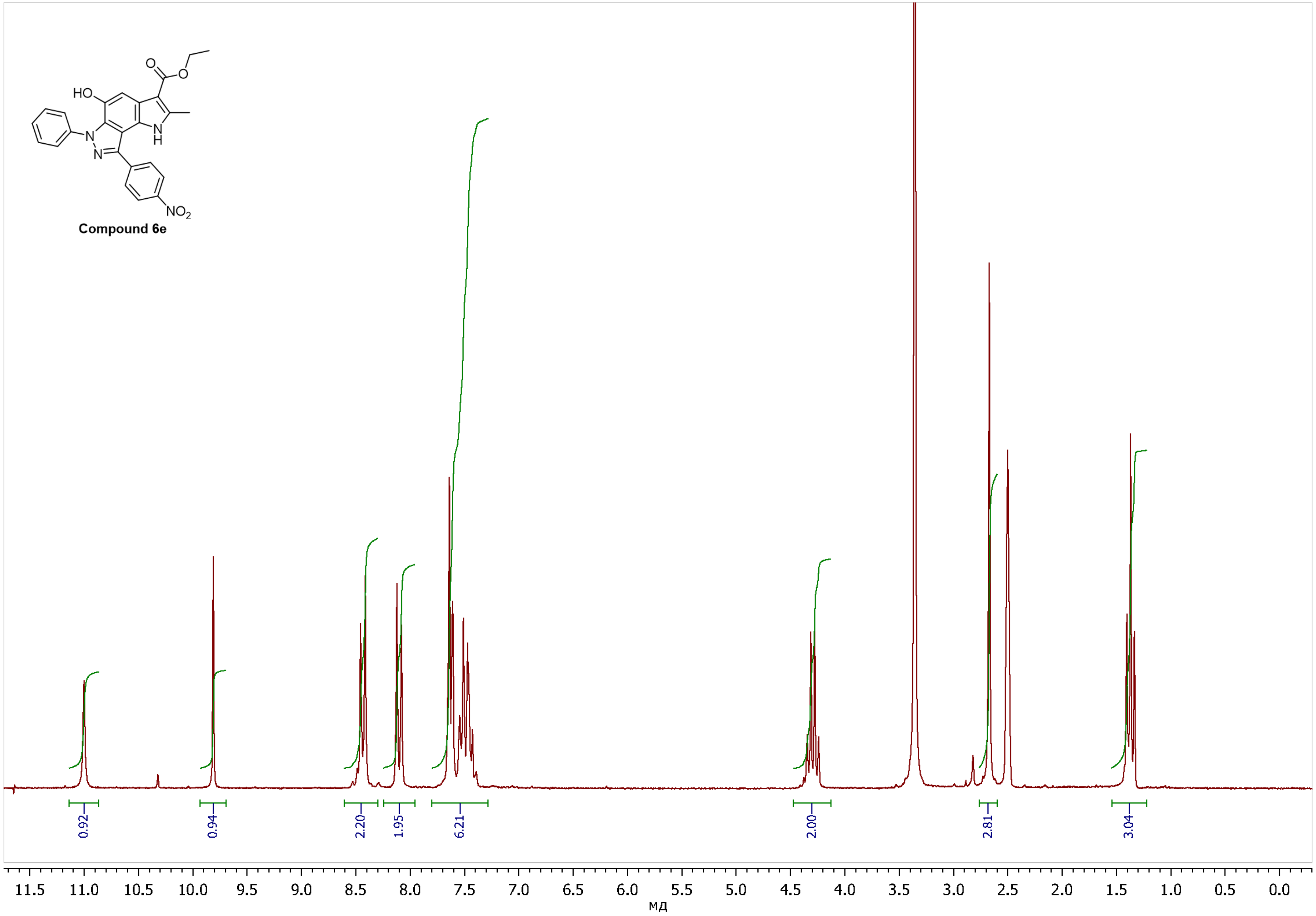

^{13}C NMR spectrum (50 MHz, DMSO-d_6) of compound **6e**

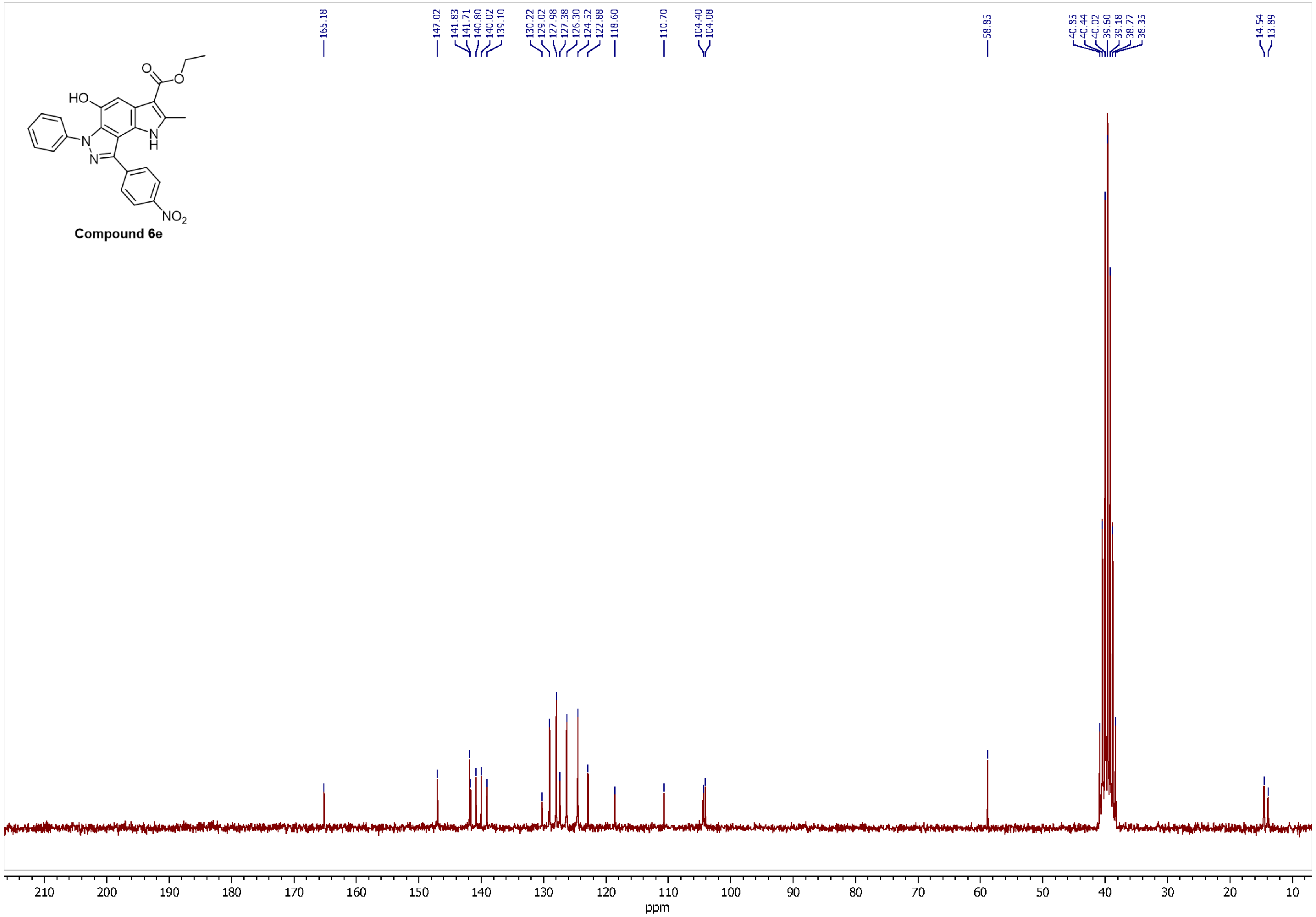

^{1}H NMR spectrum (200 MHz, DMSO-d_6) of compound **6f**

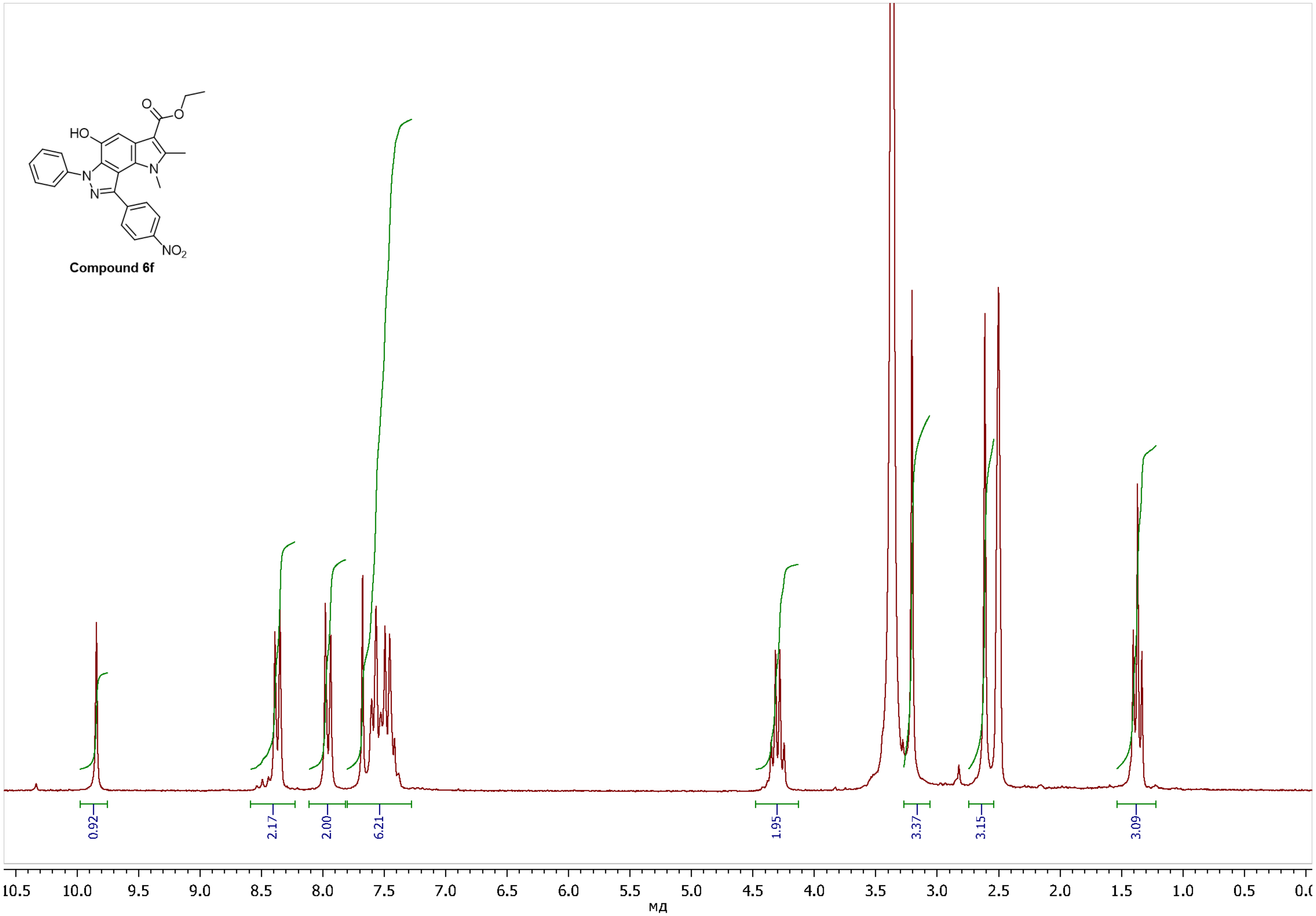

^{13}C NMR spectrum (50 MHz, DMSO-d_6) of compound **6f**

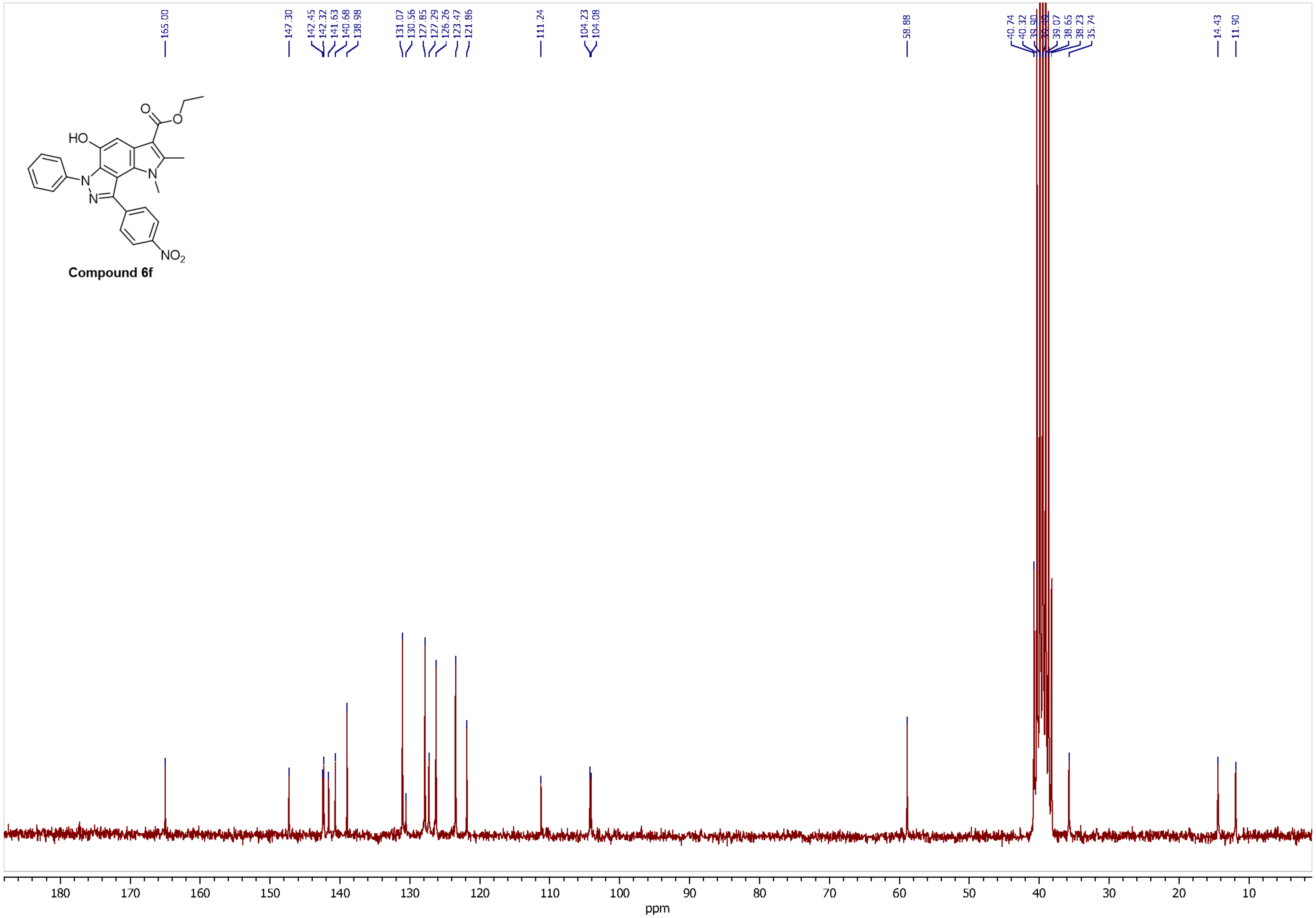

^{1}H NMR spectrum (200 MHz, DMSO-d_6) of compound **6g**

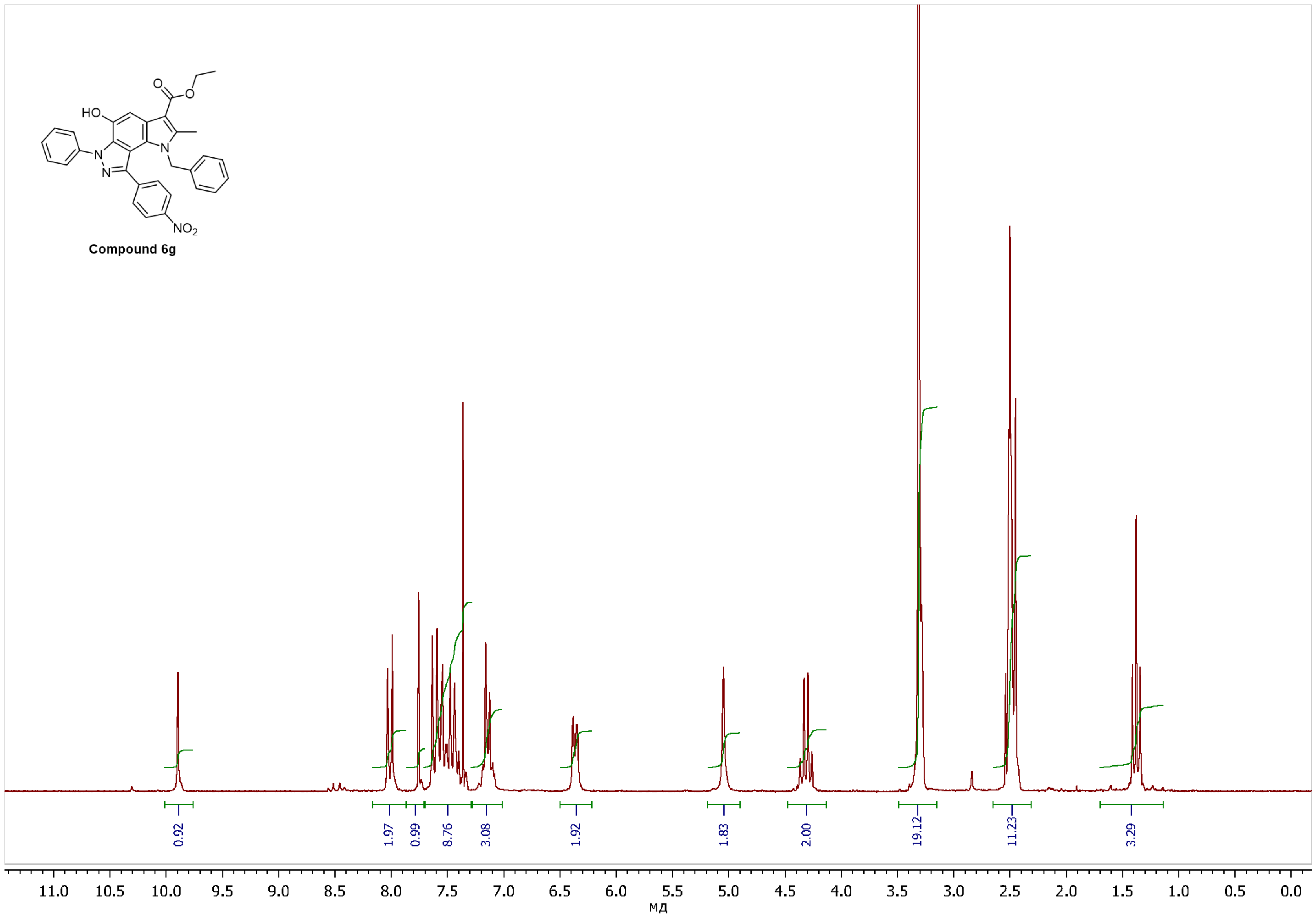

^{13}C NMR spectrum (50 MHz, DMSO-d$_6$) of compound **6g**

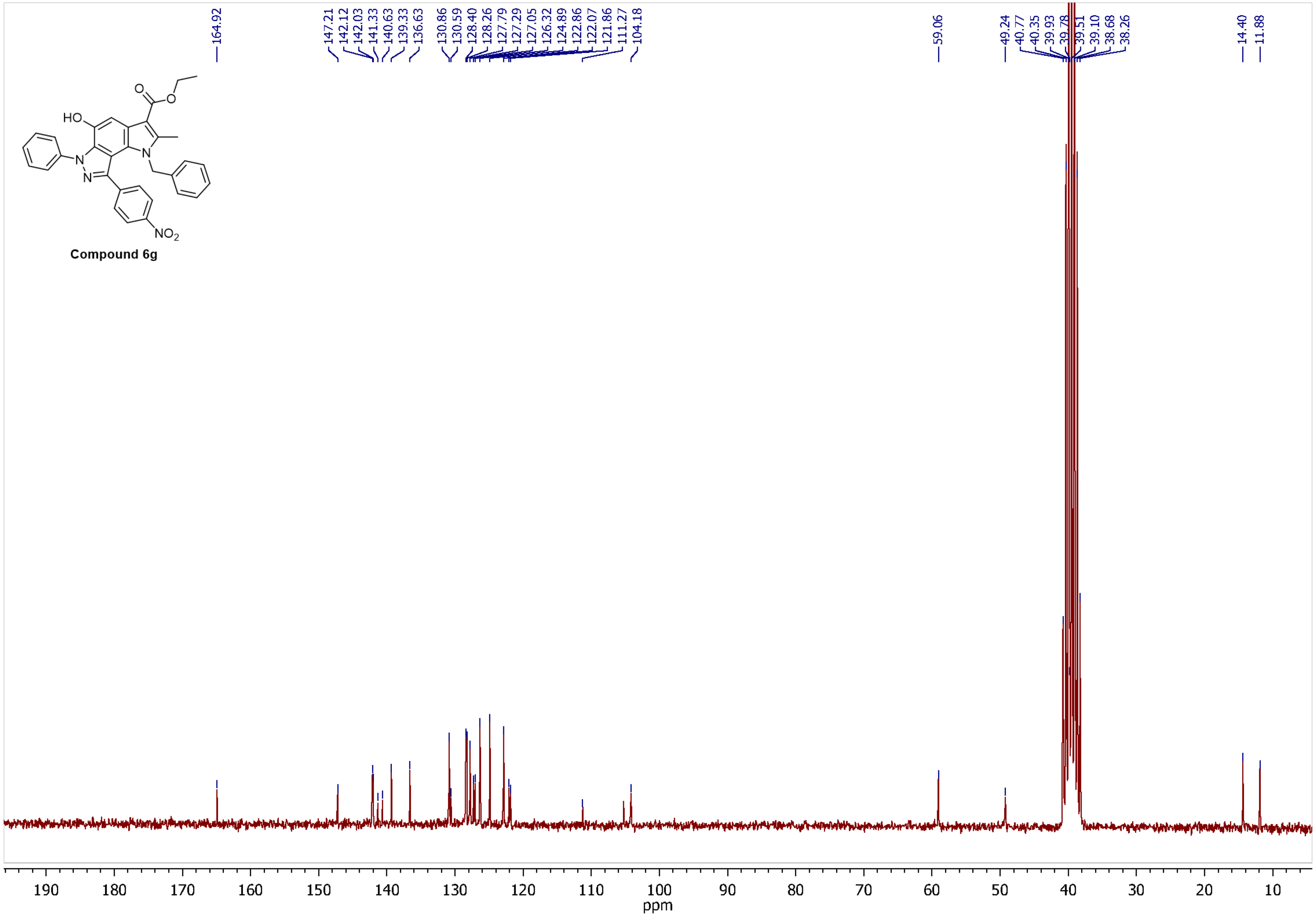

^{1}H NMR spectrum (500 MHz, DMSO-d_6) of compound **6h**

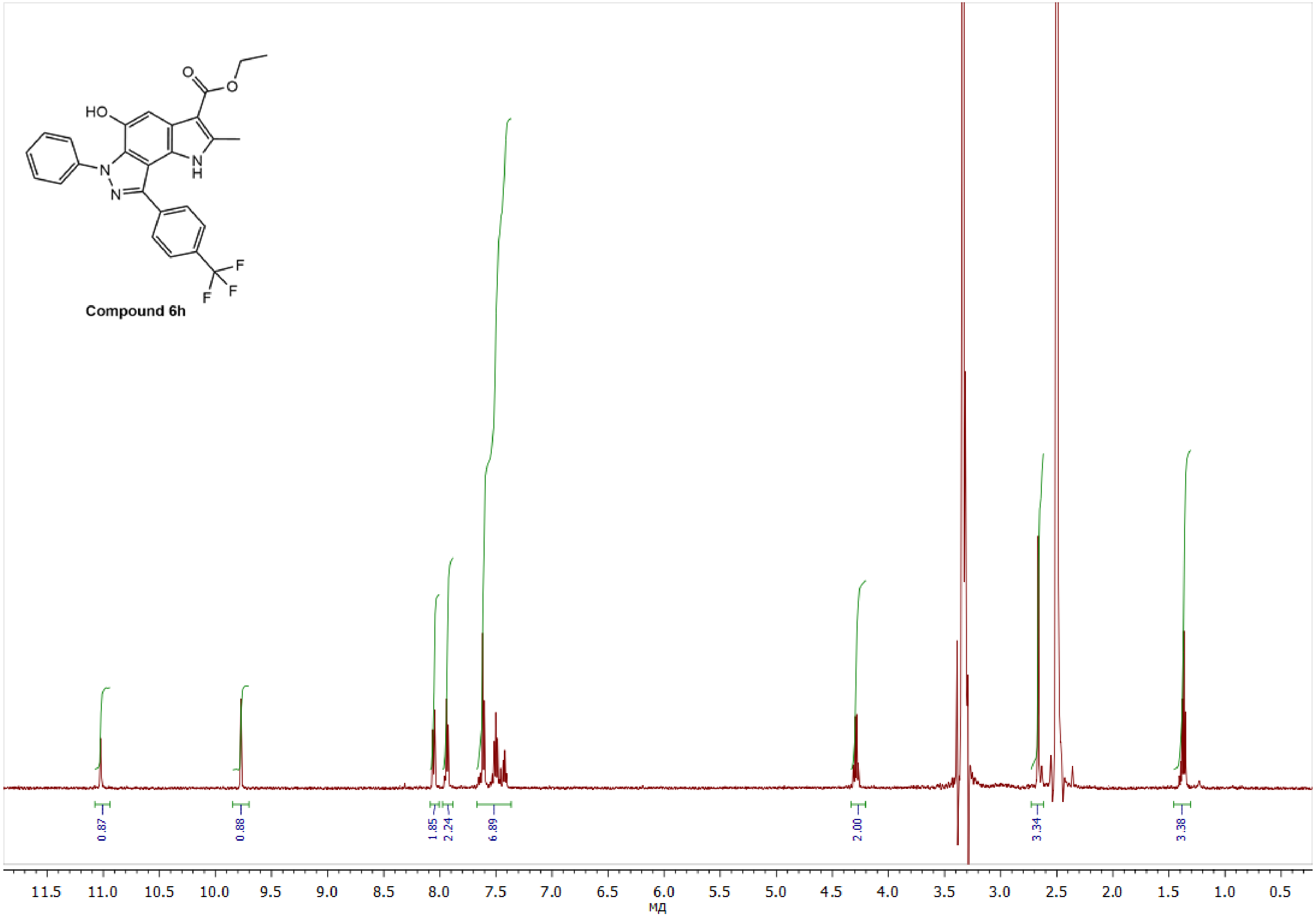

^{13}C NMR spectrum (126 MHz, DMSO-d_6) of compound **6h**

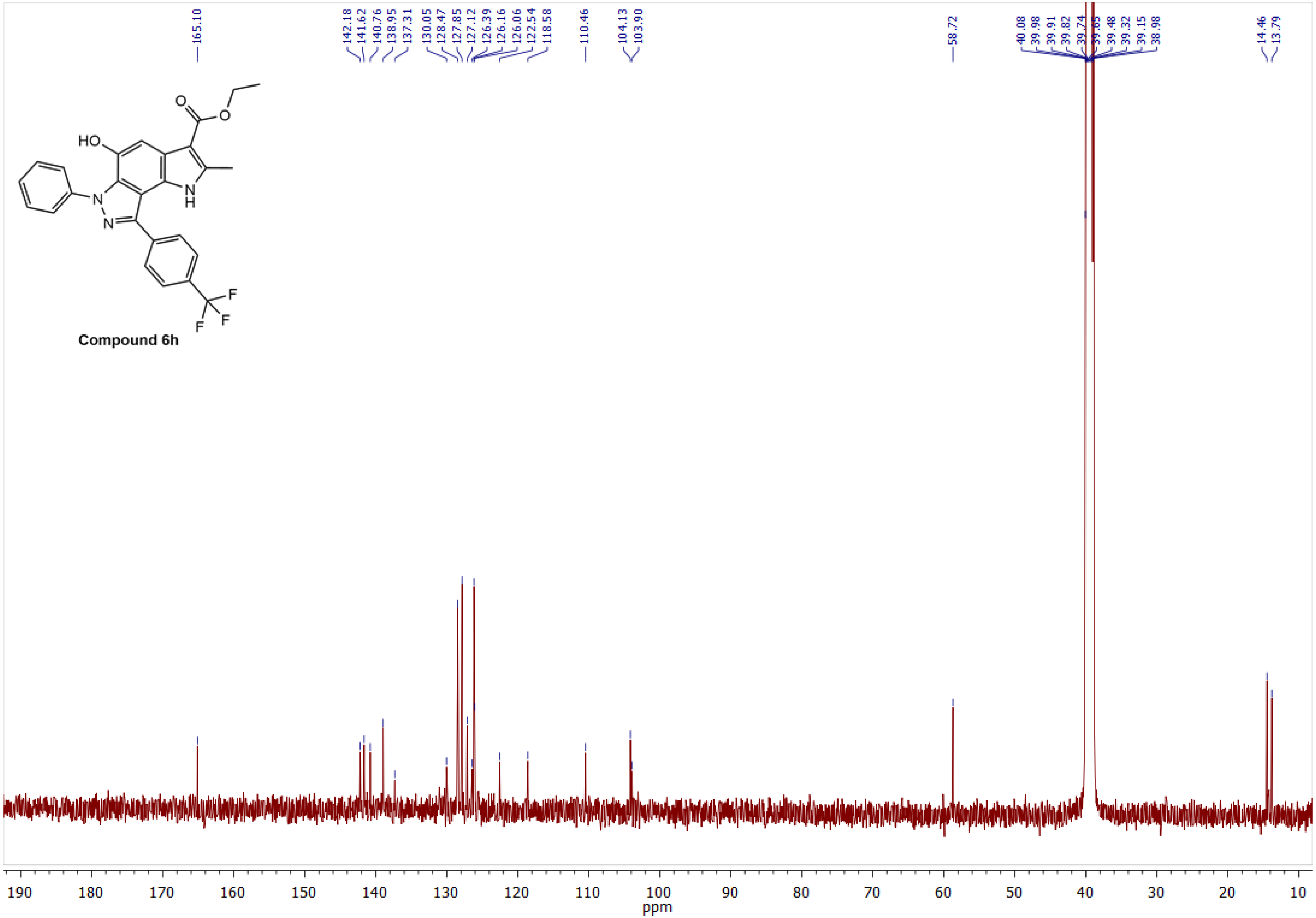

^{1}H NMR spectrum (500 MHz, DMSO-d_6) of compound **6i**

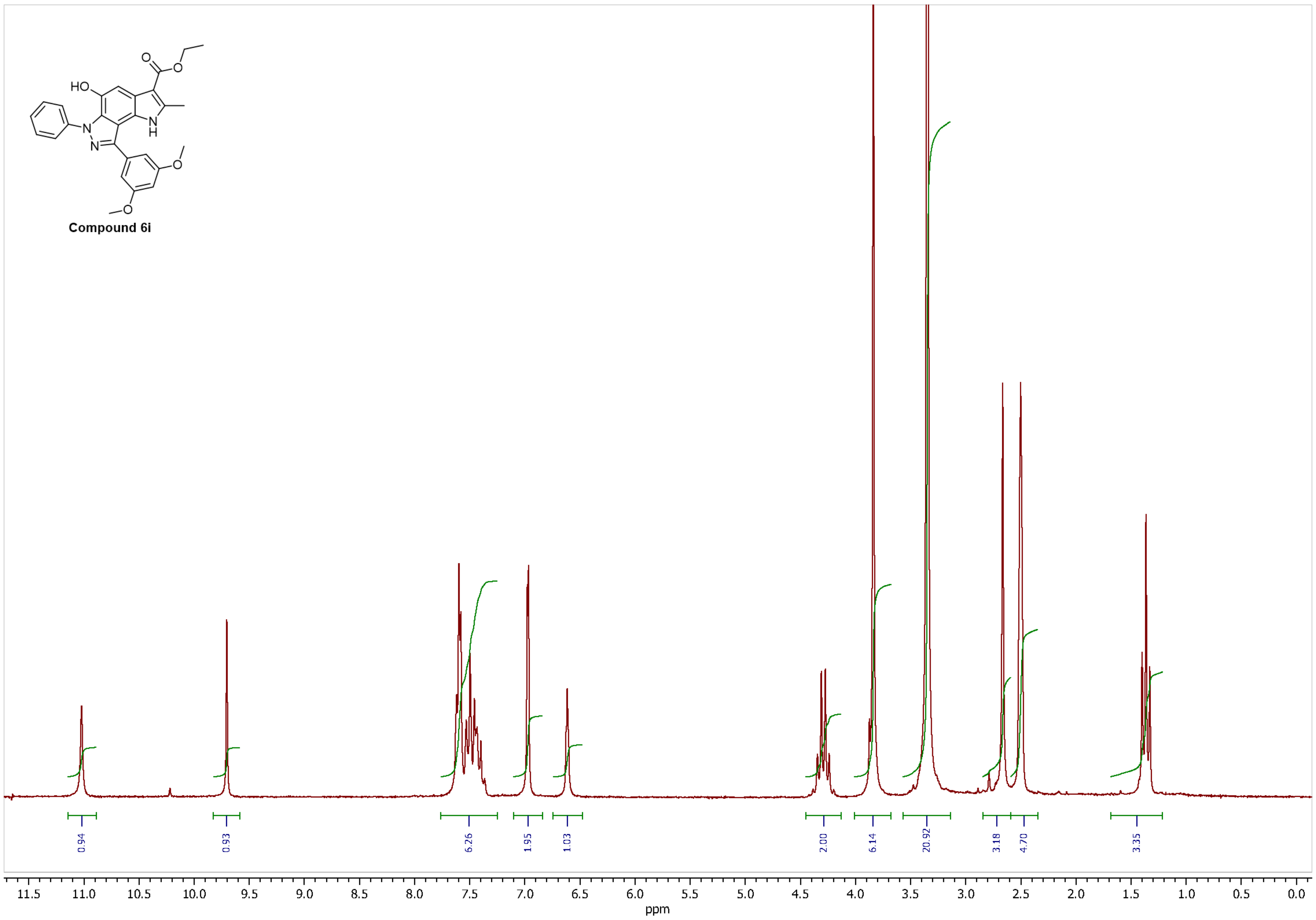

^{13}C NMR spectrum (126 MHz, DMSO-d_6) of compound **6i**

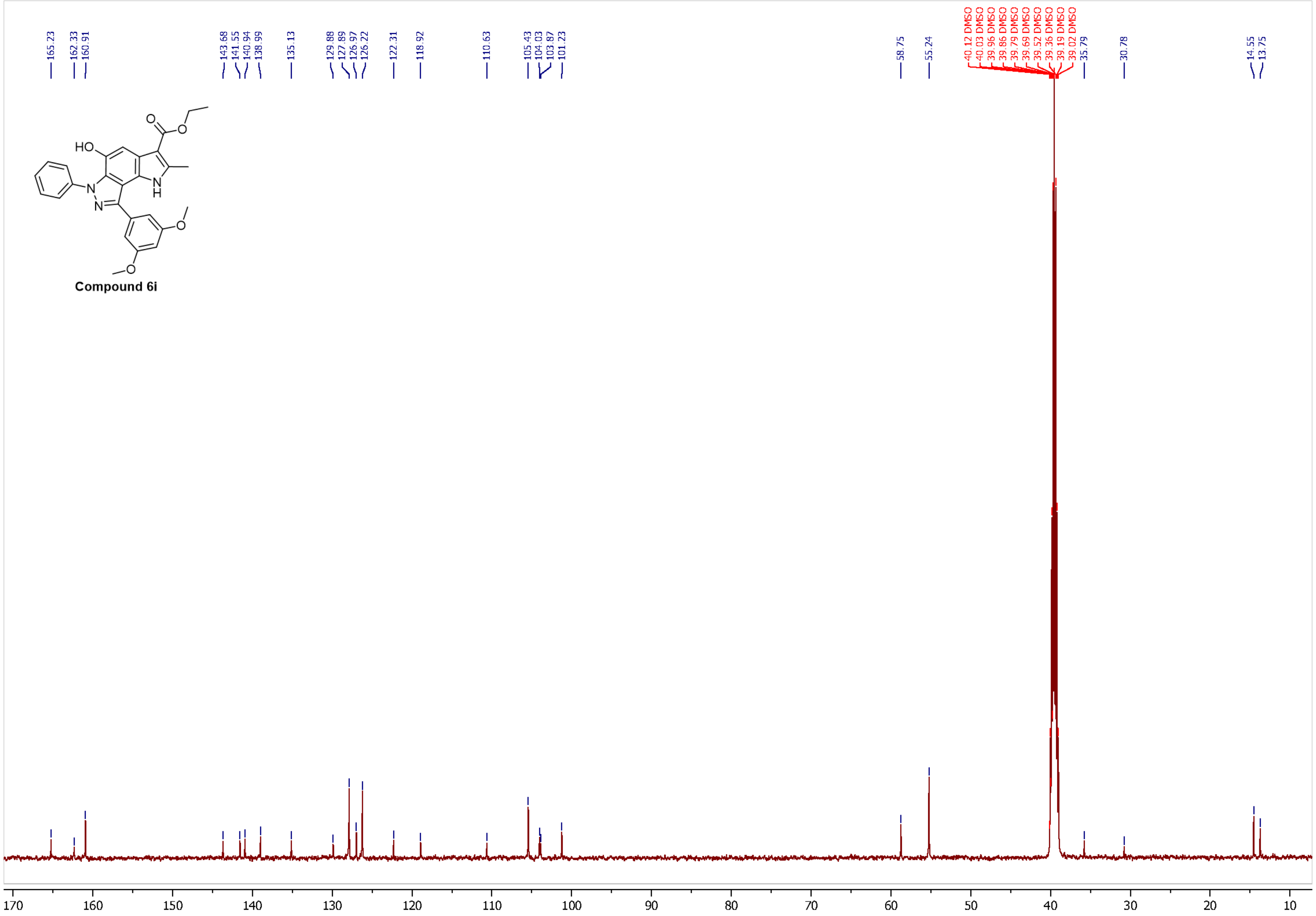

^{1}H NMR spectrum (500 MHz, DMSO-d_6) of compound **6j**

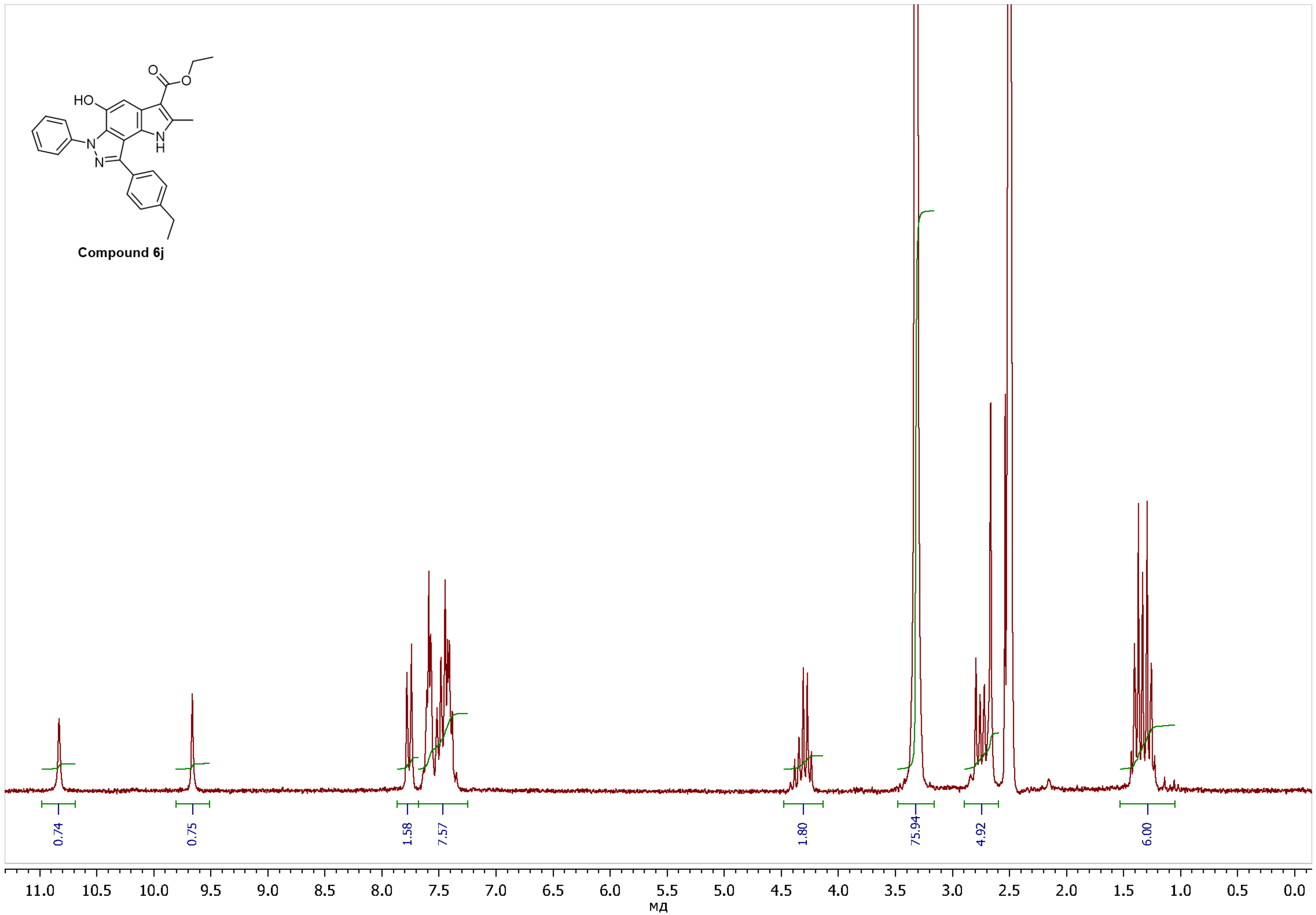

^{13}C NMR spectrum (300 MHz, DMSO-d_6) of compound **6j**

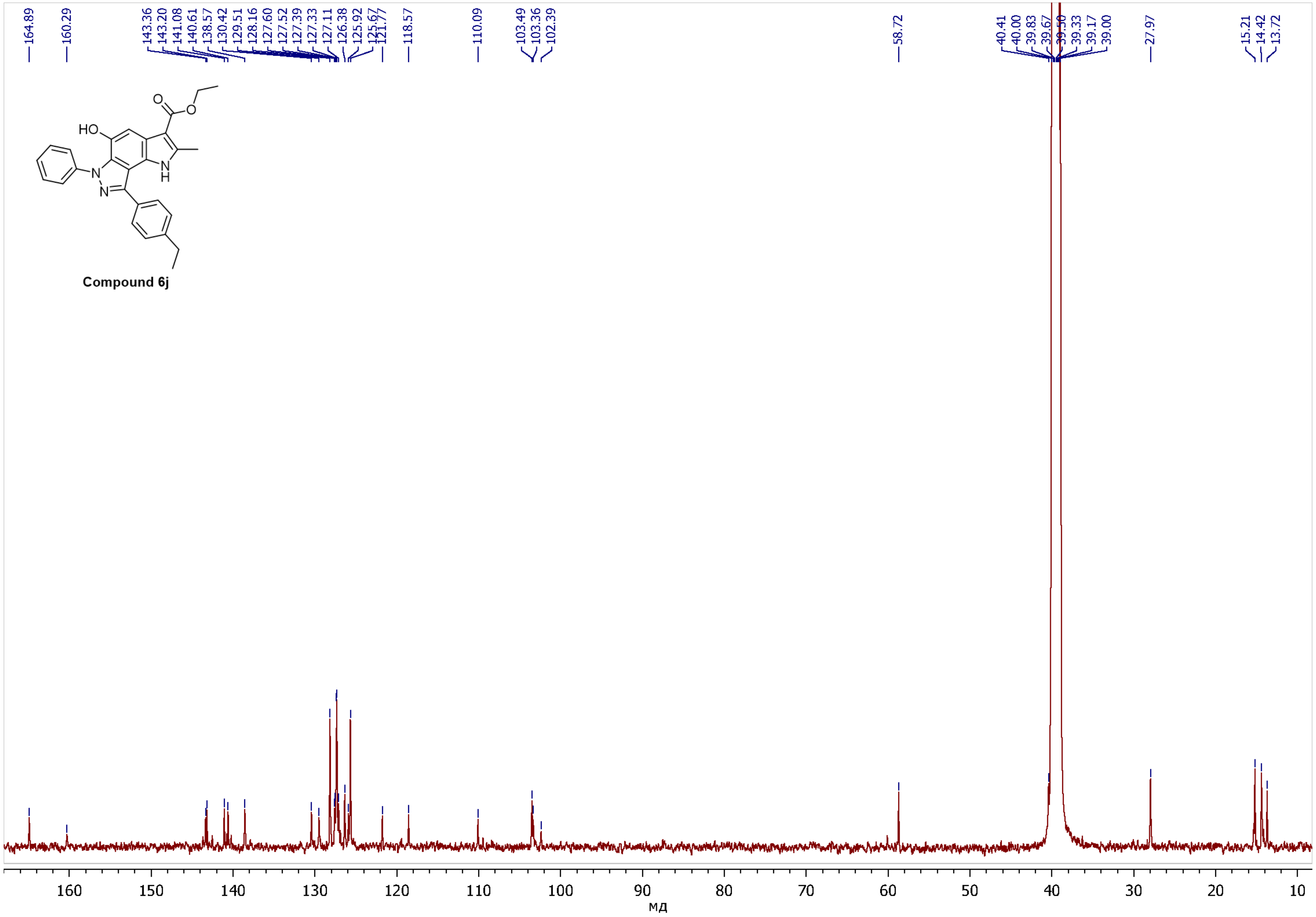

^{1}H NMR spectrum (200 MHz, DMSO-d$_6$) of compound **6k**

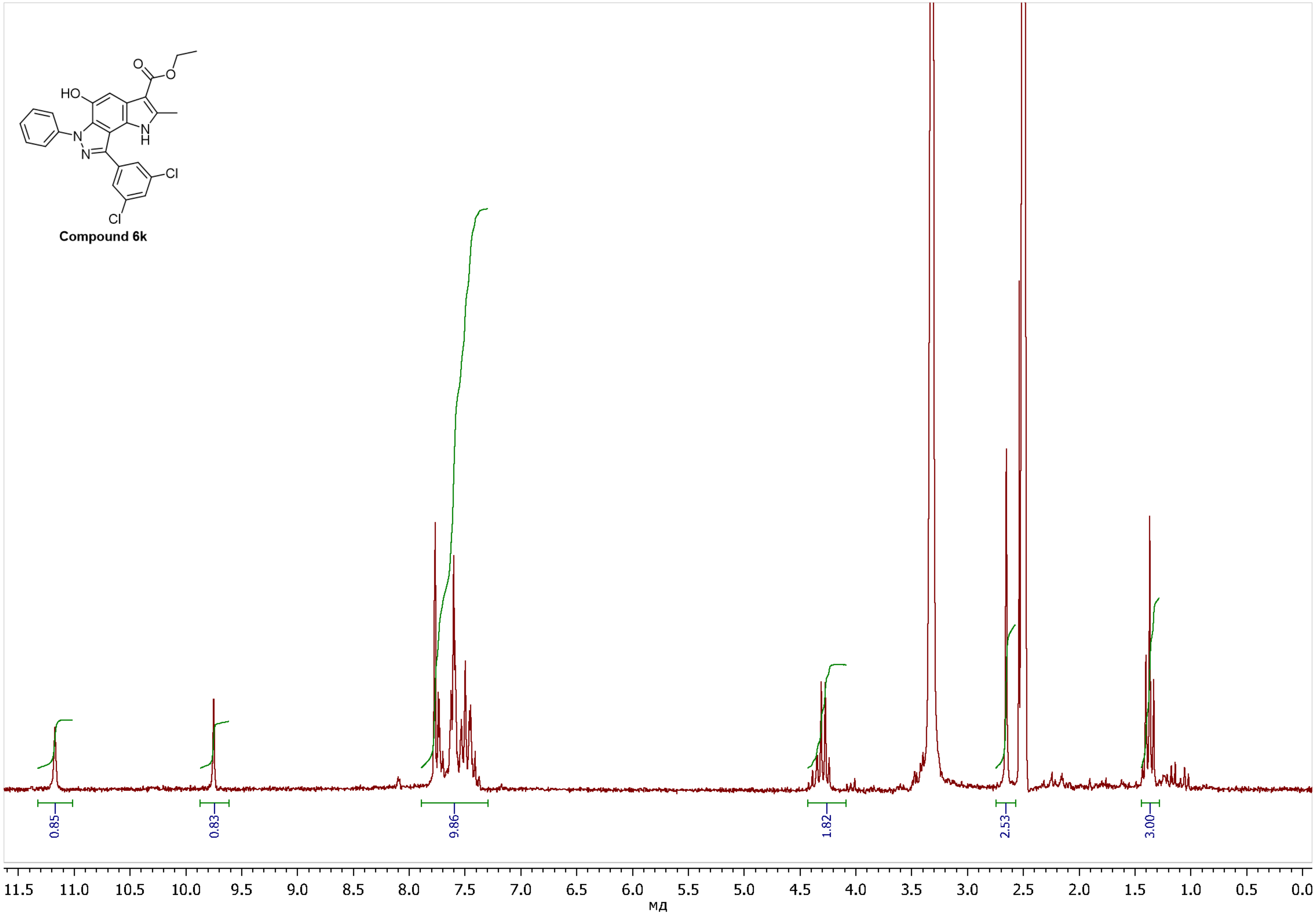

^{13}C NMR spectrum (300 MHz, DMSO-d_6) of compound **6k**

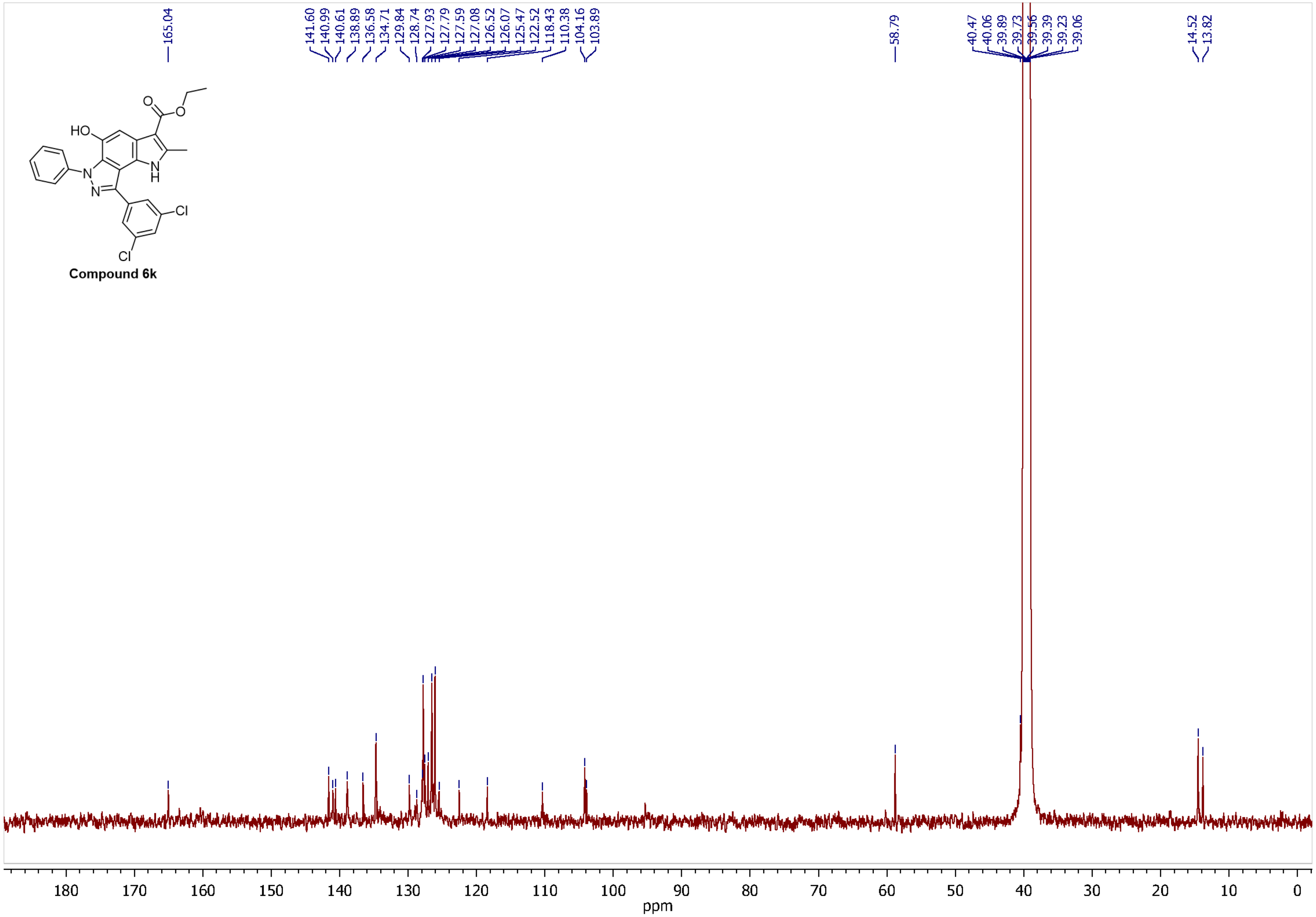

^{1}H NMR spectrum (200 MHz, DMSO-d_6) of compound **7a**

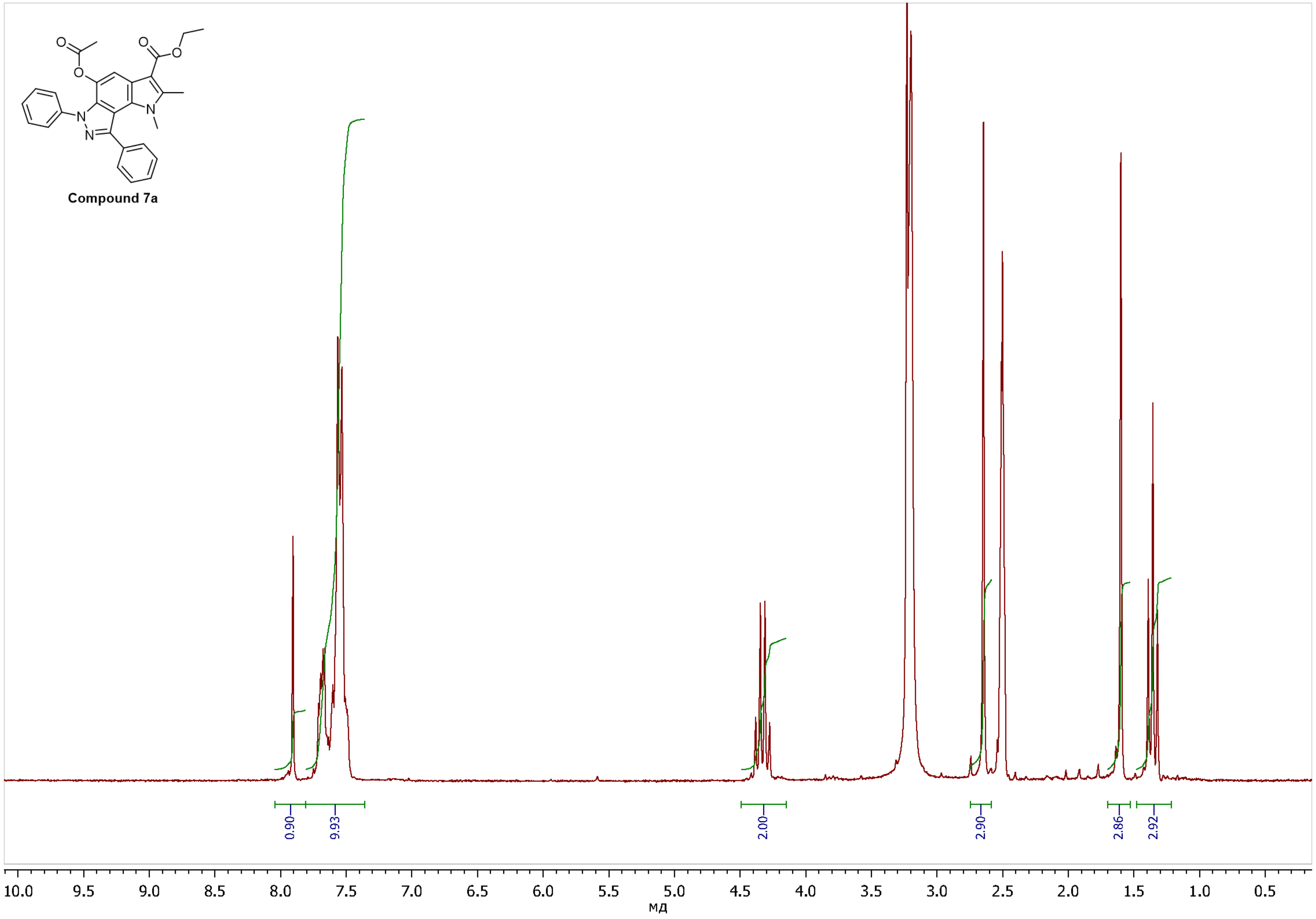

^{13}C NMR spectrum (50 MHz, DMSO-d_6) of compound **7a**

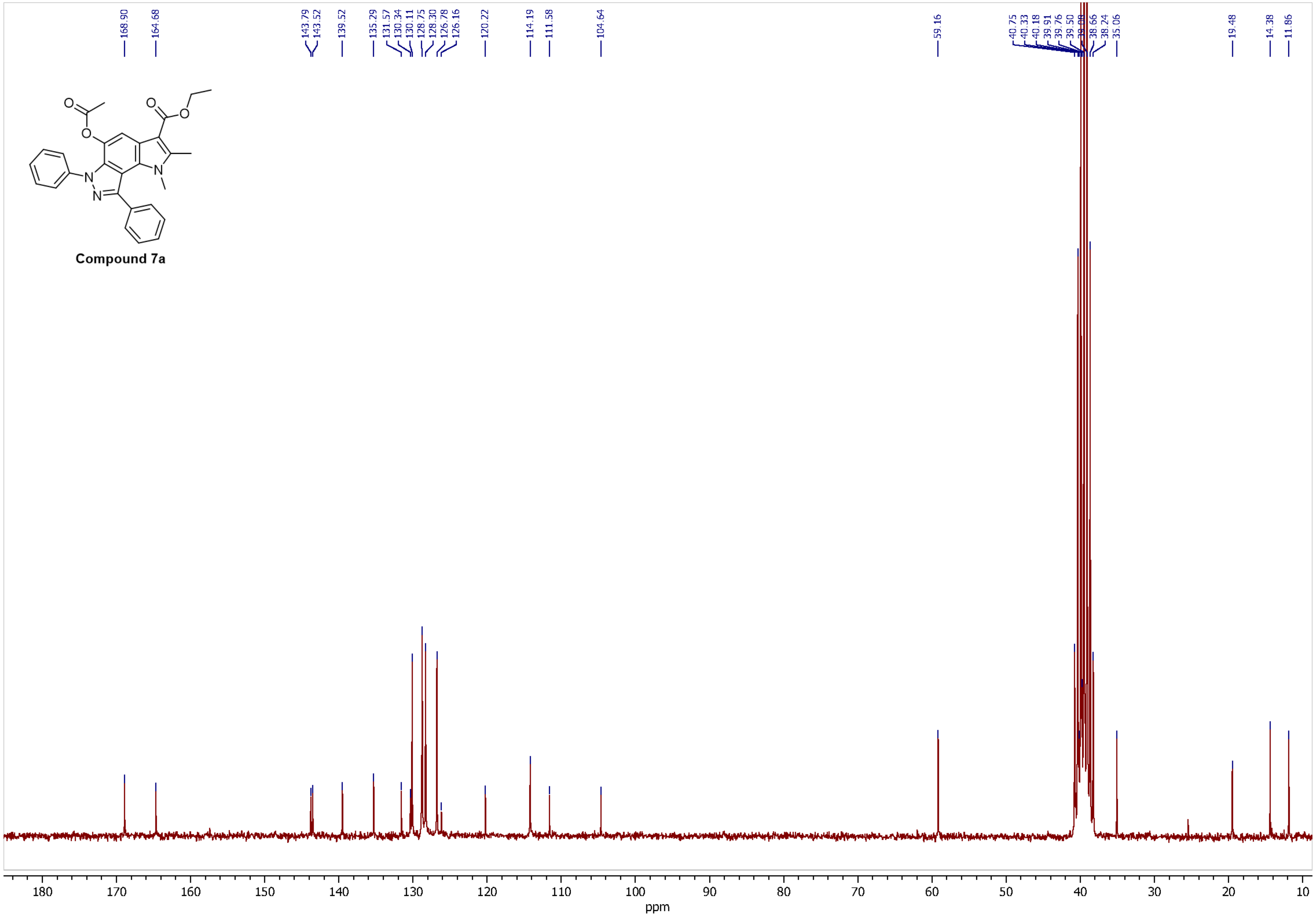

^{1}H NMR spectrum (200 MHz, DMSO-d$_6$) of compound **7b**

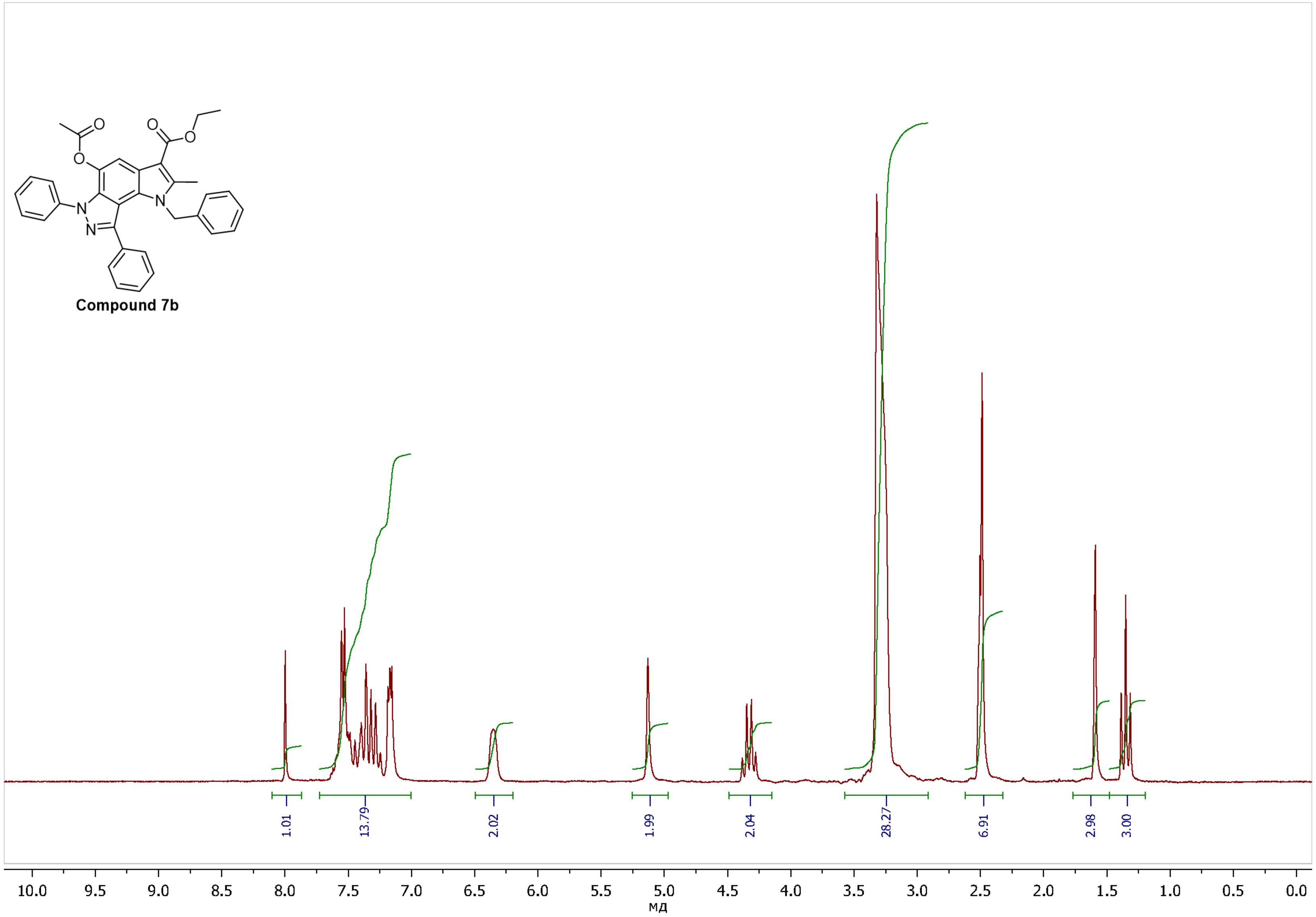

^{13}C NMR spectrum (50 MHz, DMSO-d_6) of compound **7b**

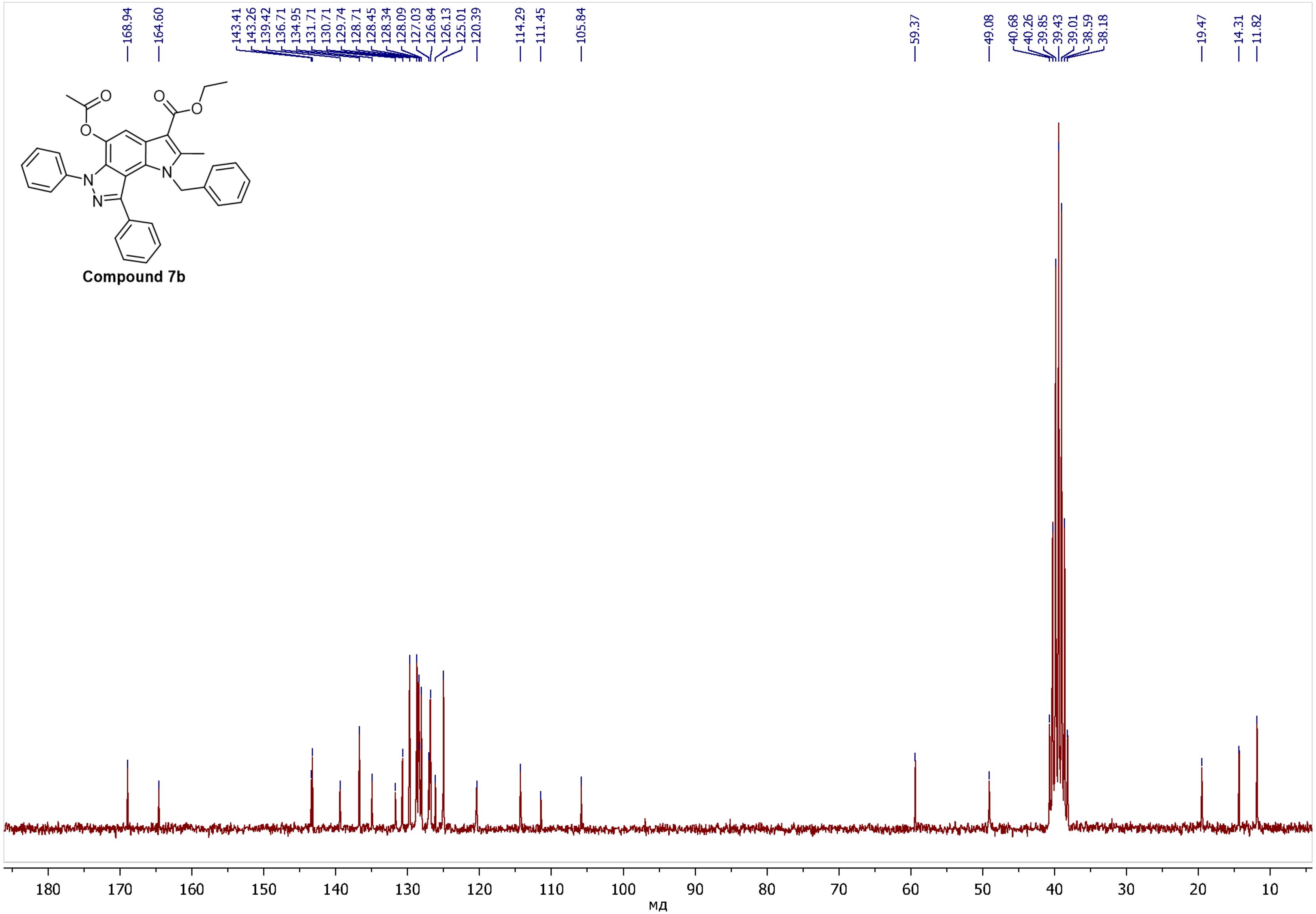

^1H NMR spectrum (200 MHz, DMSO-d_6) of compound **9a**

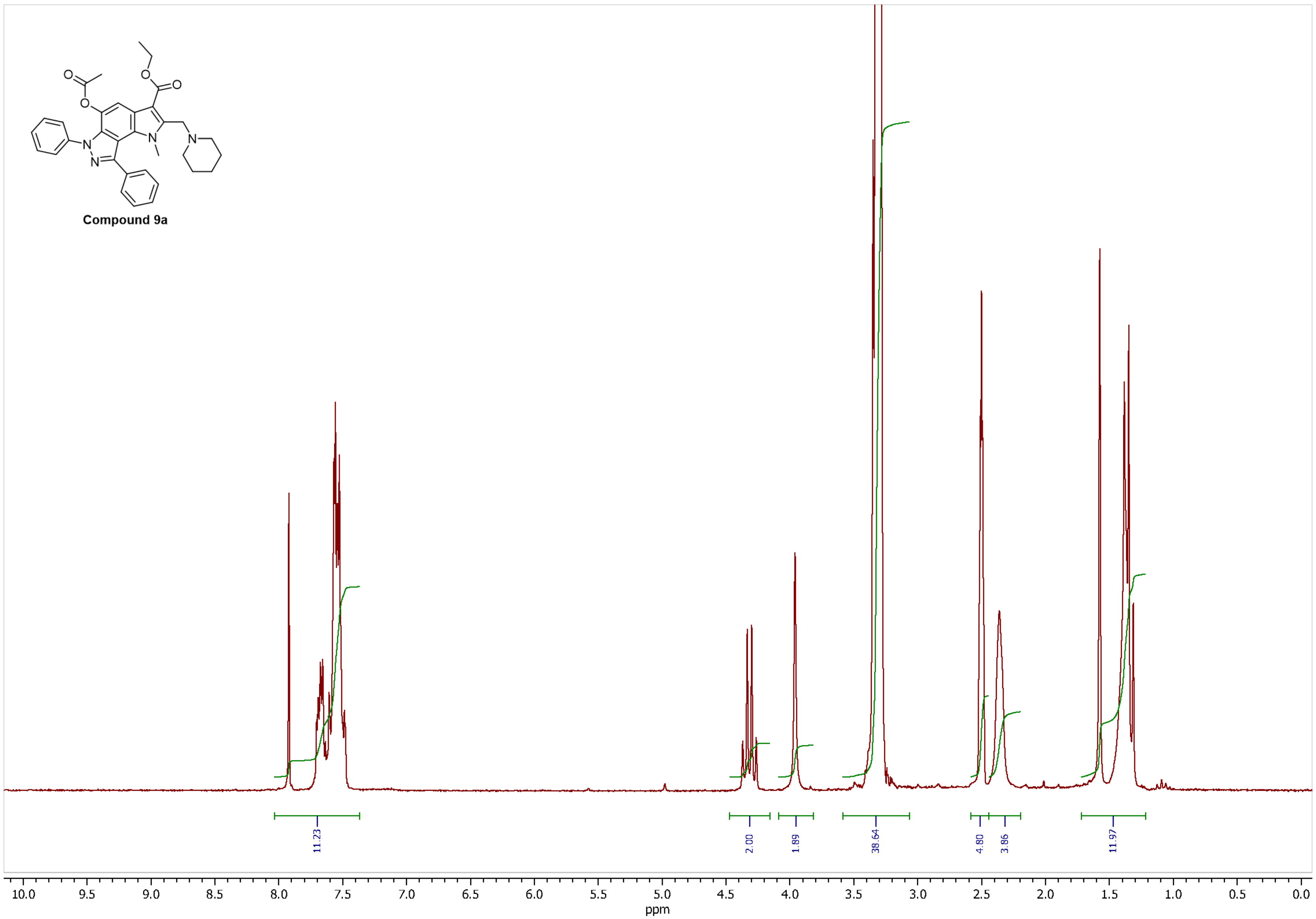

^{13}C NMR spectrum (50 MHz, DMSO-d$_6$) of compound **9a**

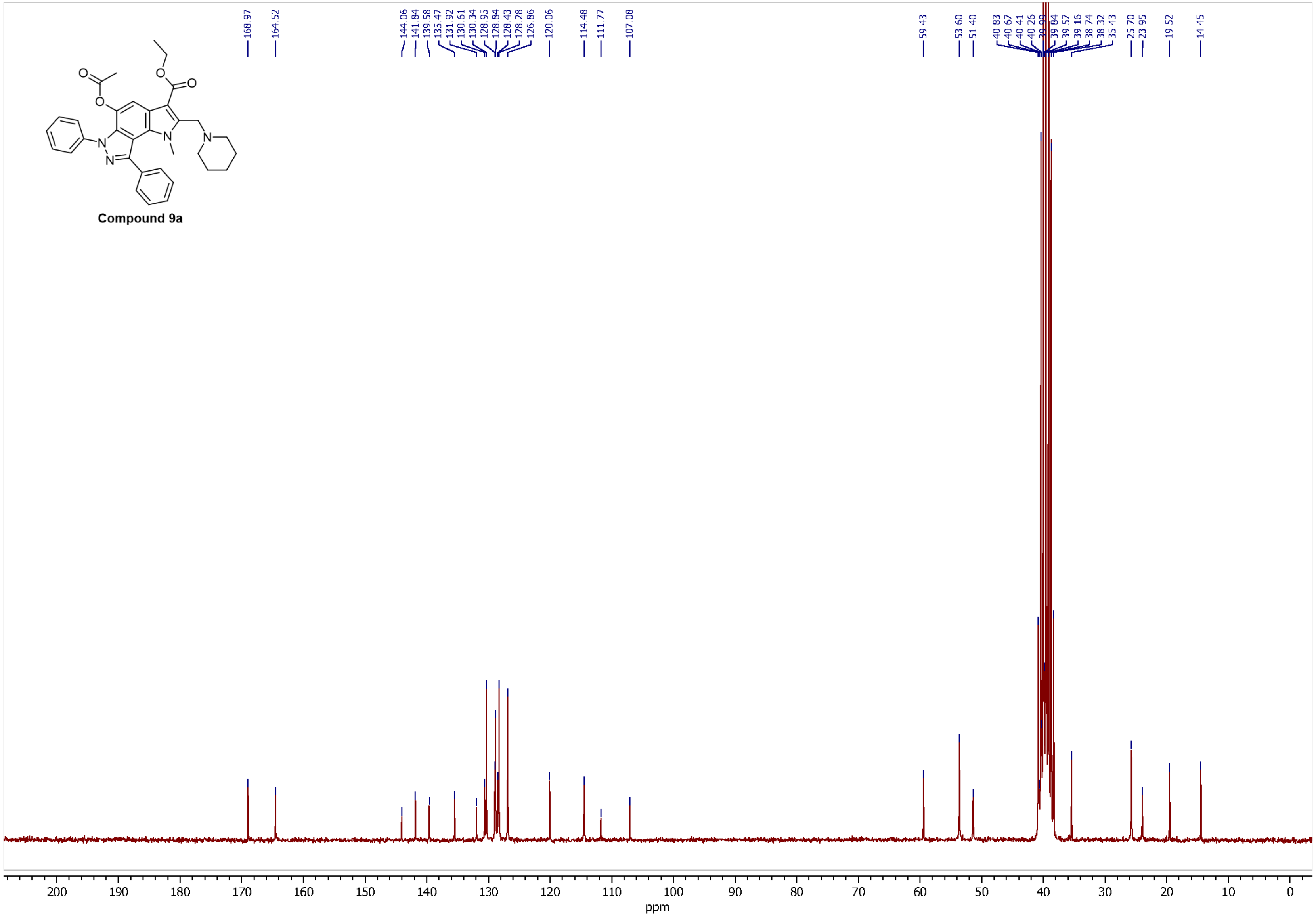

^{1}H NMR spectrum (200 MHz, DMSO-d$_6$) of compound **9b**

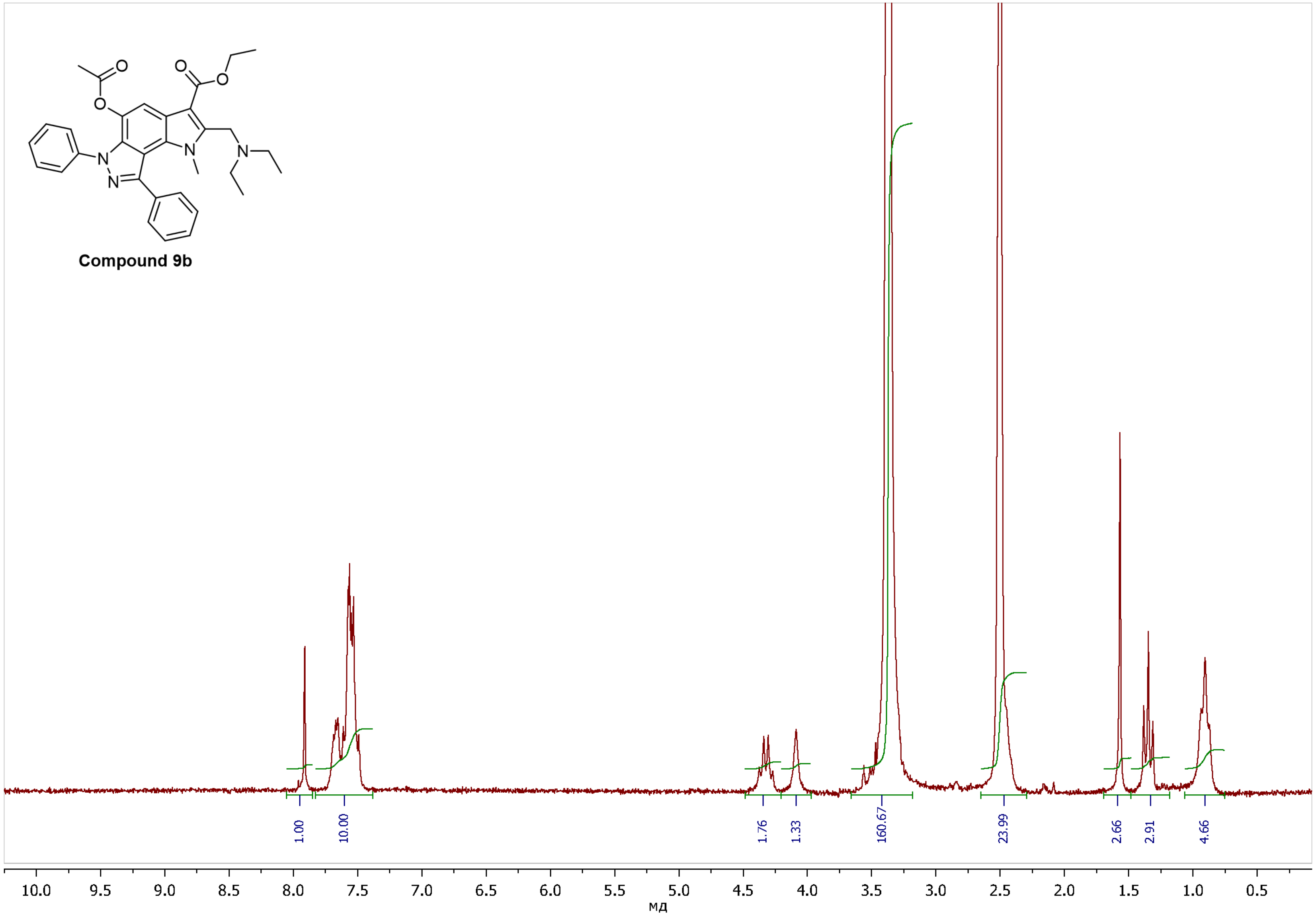

^{13}C NMR spectrum (50 MHz, DMSO-d$_6$) of compound **9b**

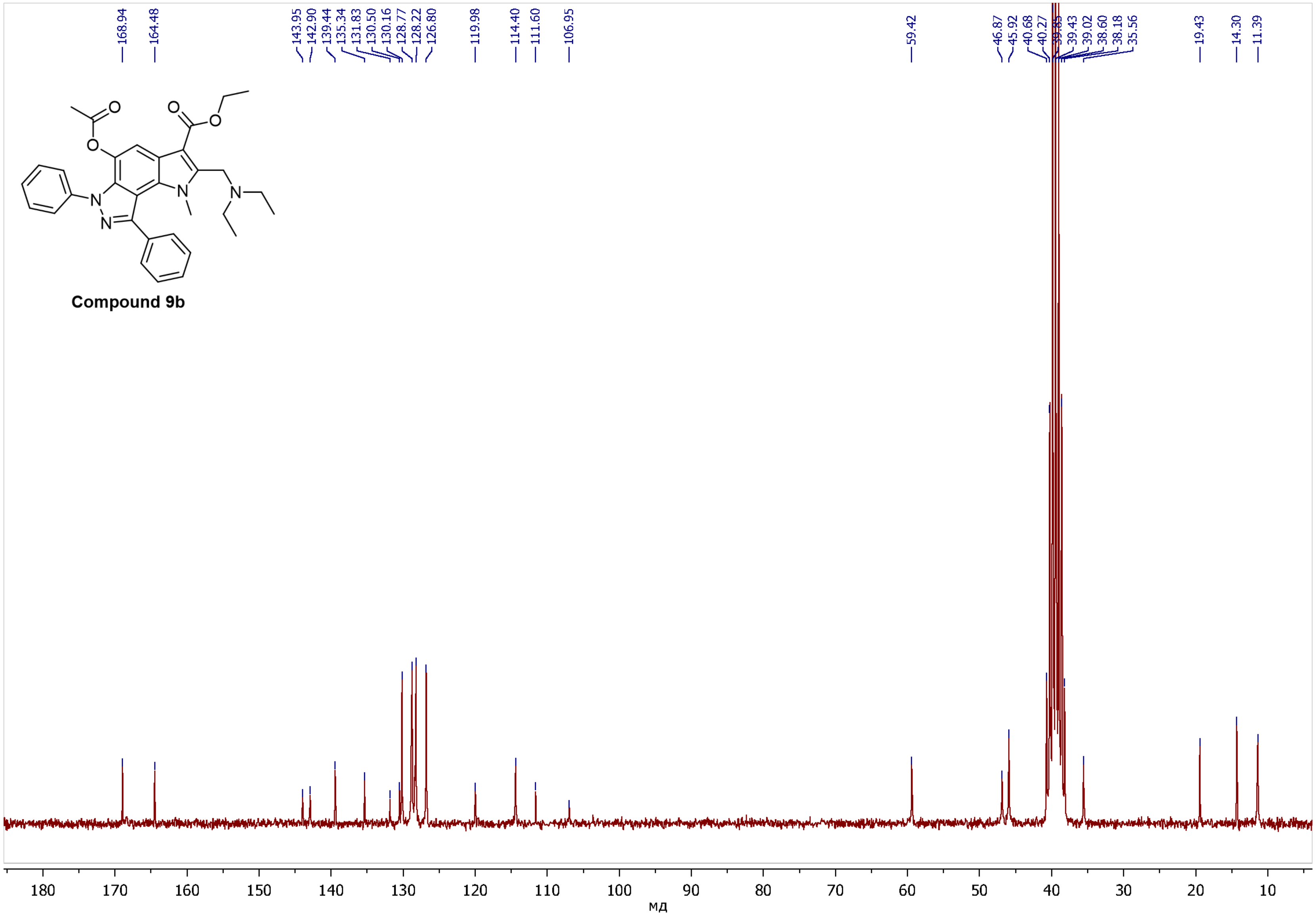

^{1}H NMR spectrum (200 MHz, DMSO-d$_6$) of compound **9c**

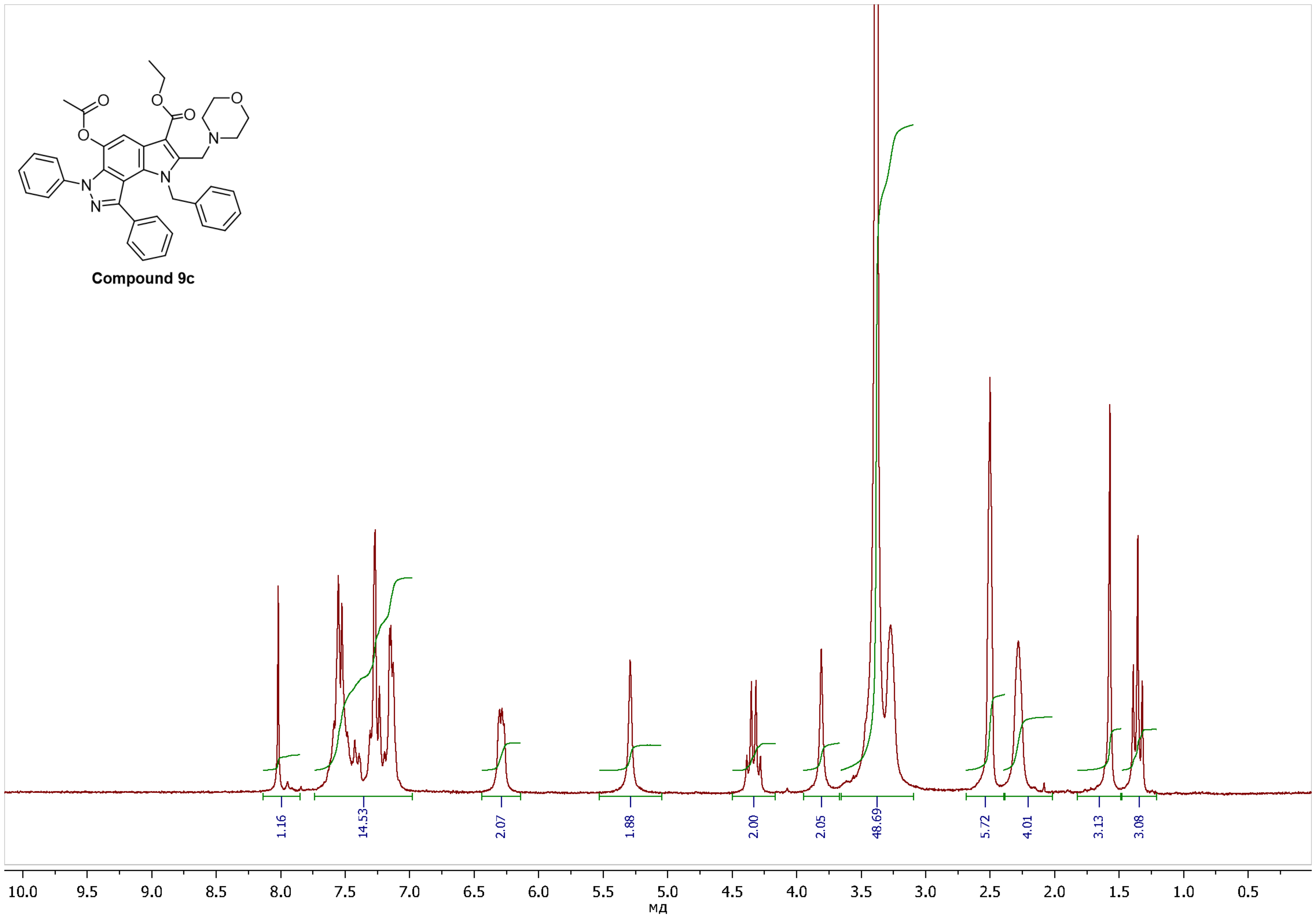

^{13}C NMR spectrum (50 MHz, DMSO-d_6) of compound **9c**

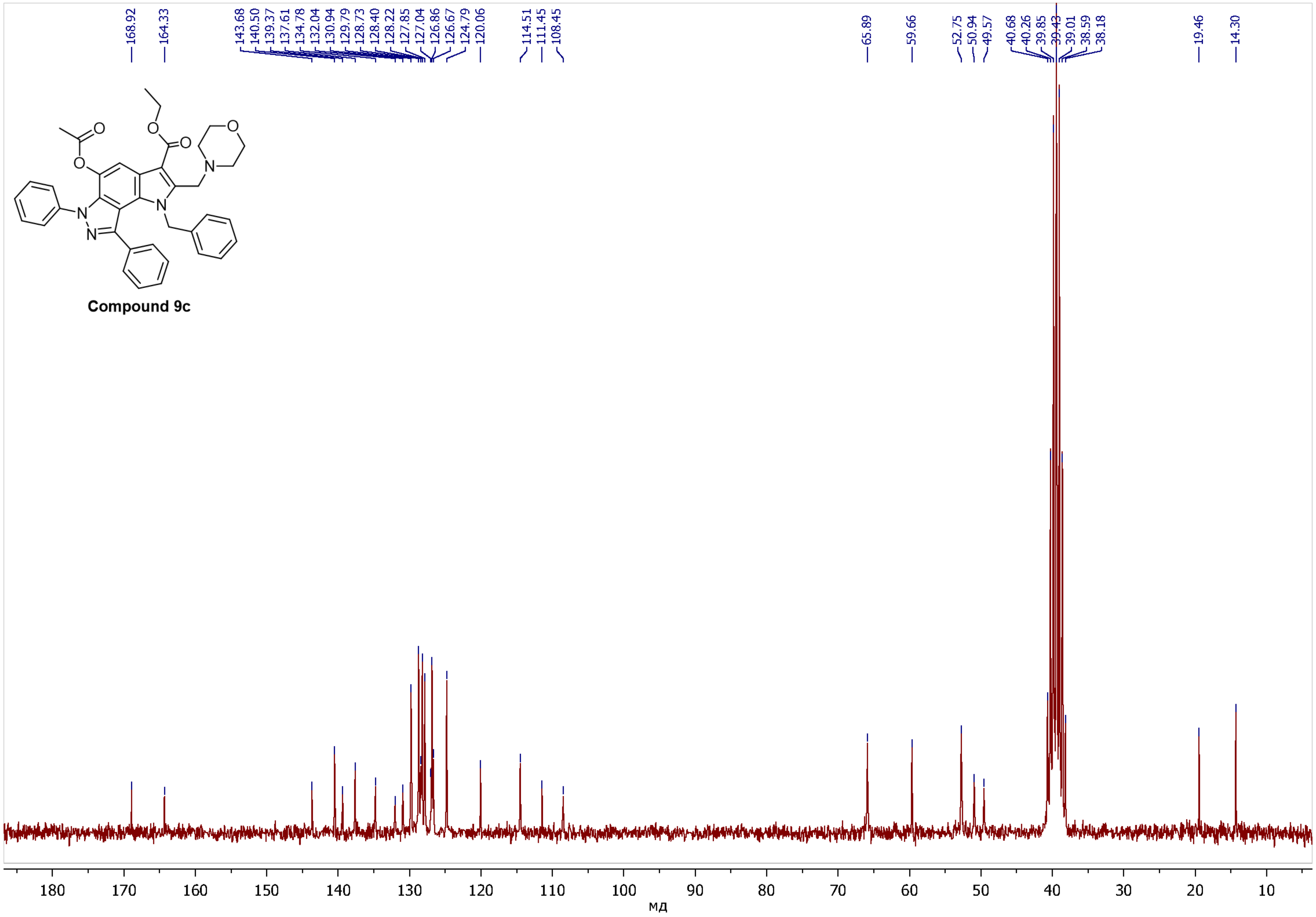

^{1}H NMR spectrum (200 MHz, DMSO-d_6) of compound **10a**

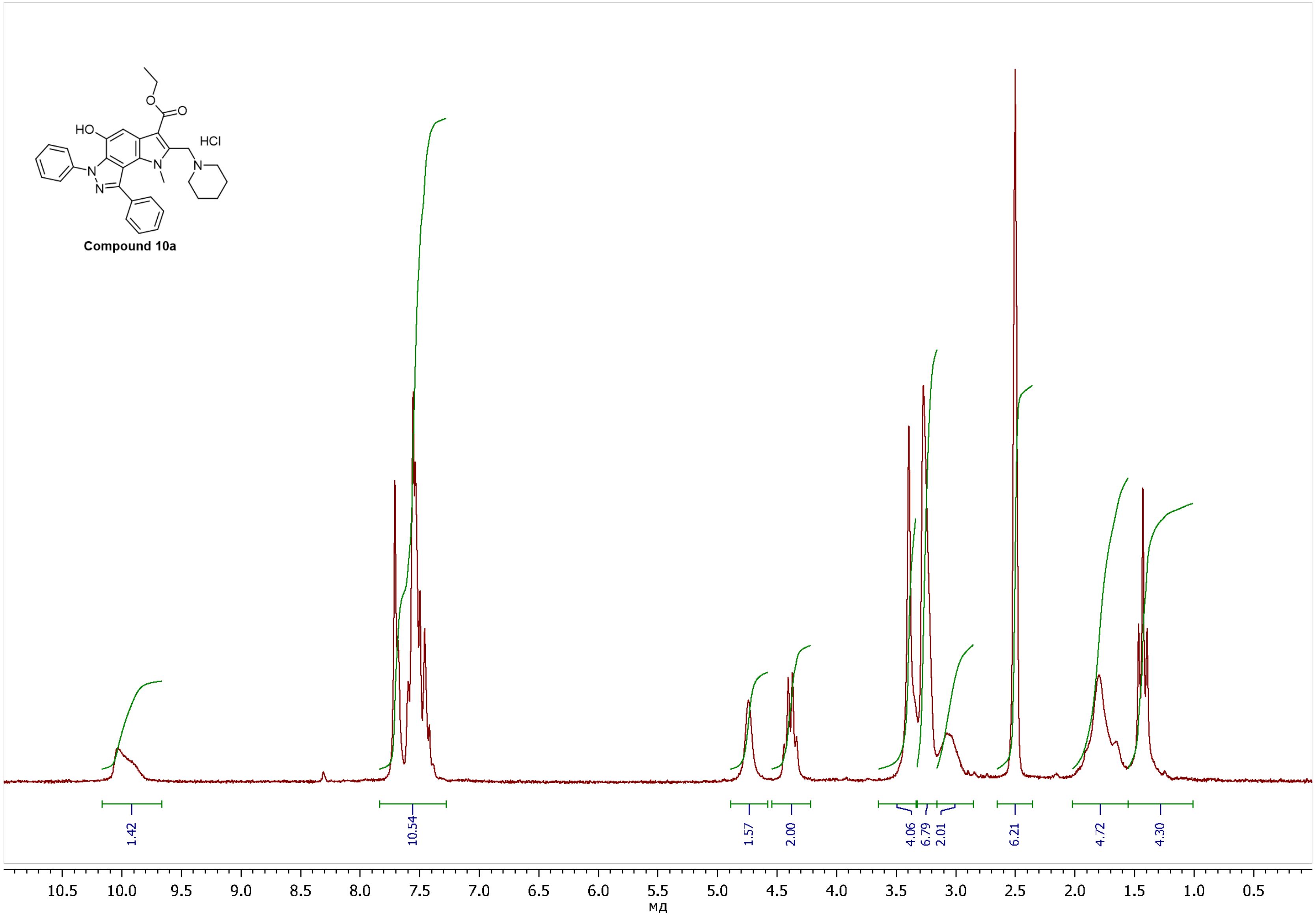

^{13}C NMR spectrum (50 MHz, DMSO-d_6) of compound **10a**

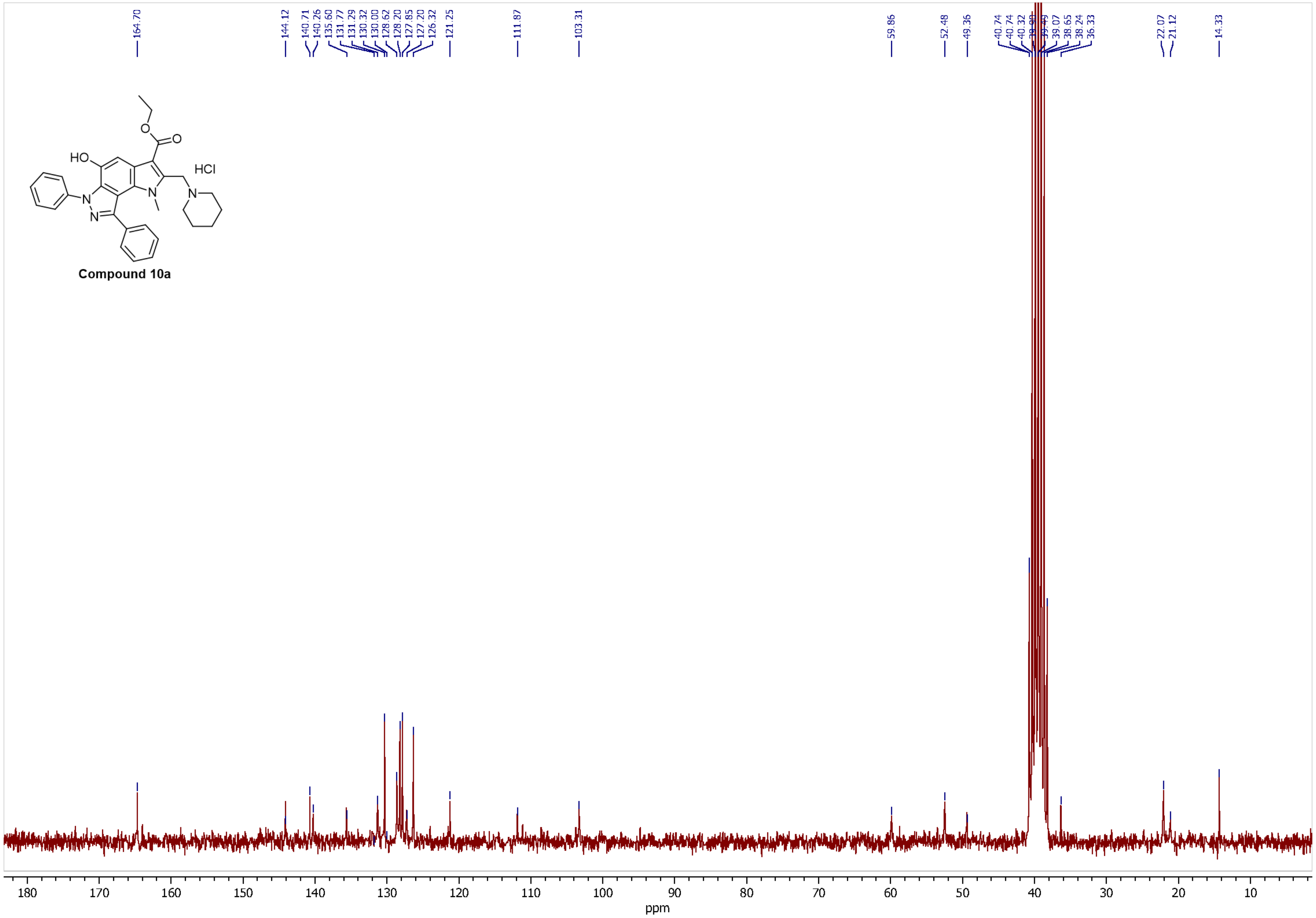

^{1}H NMR spectrum (200 MHz, DMSO-d_6) of compound **10b**

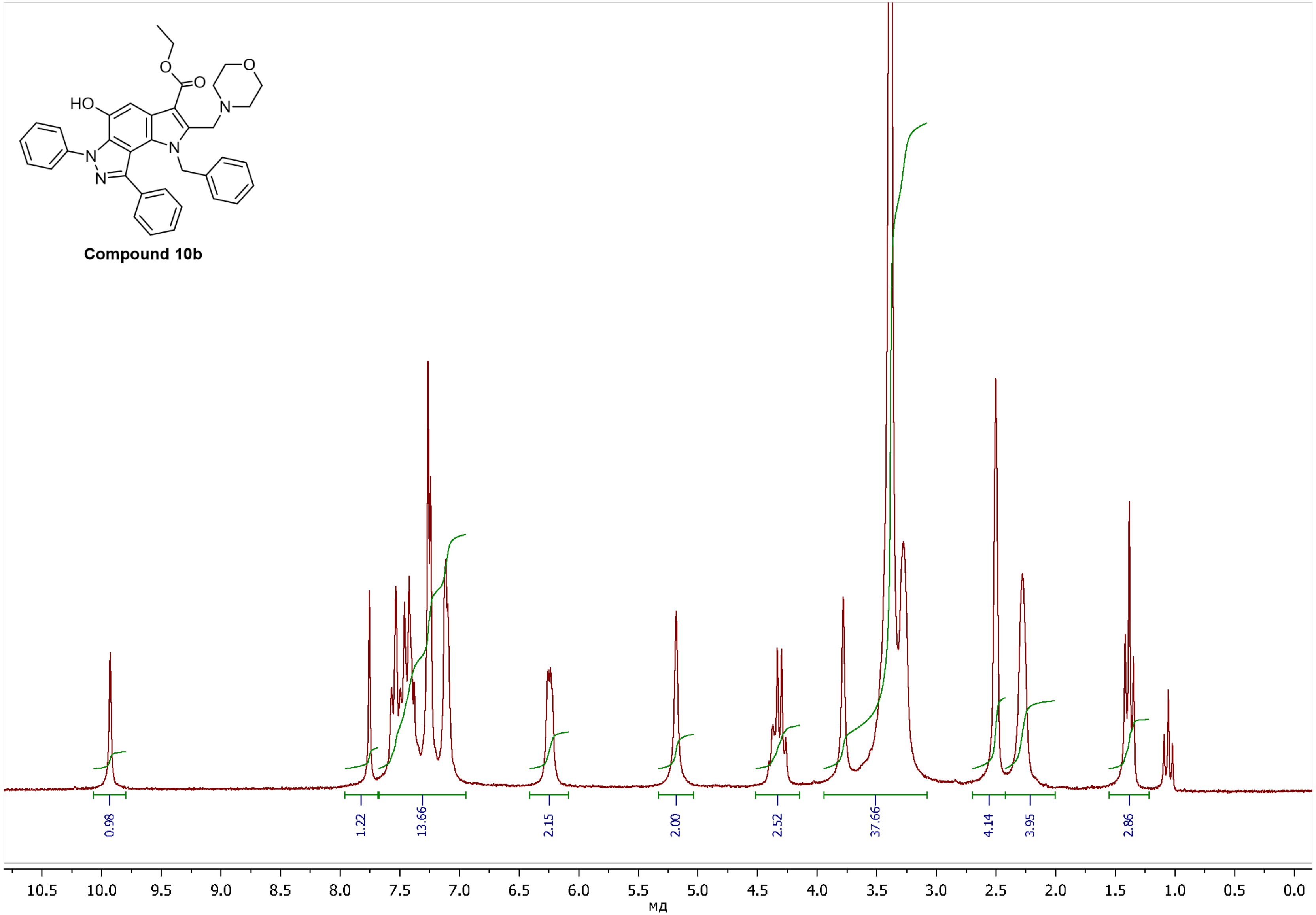

^{13}C NMR spectrum (50 MHz, DMSO-d$_6$) of compound **10b**

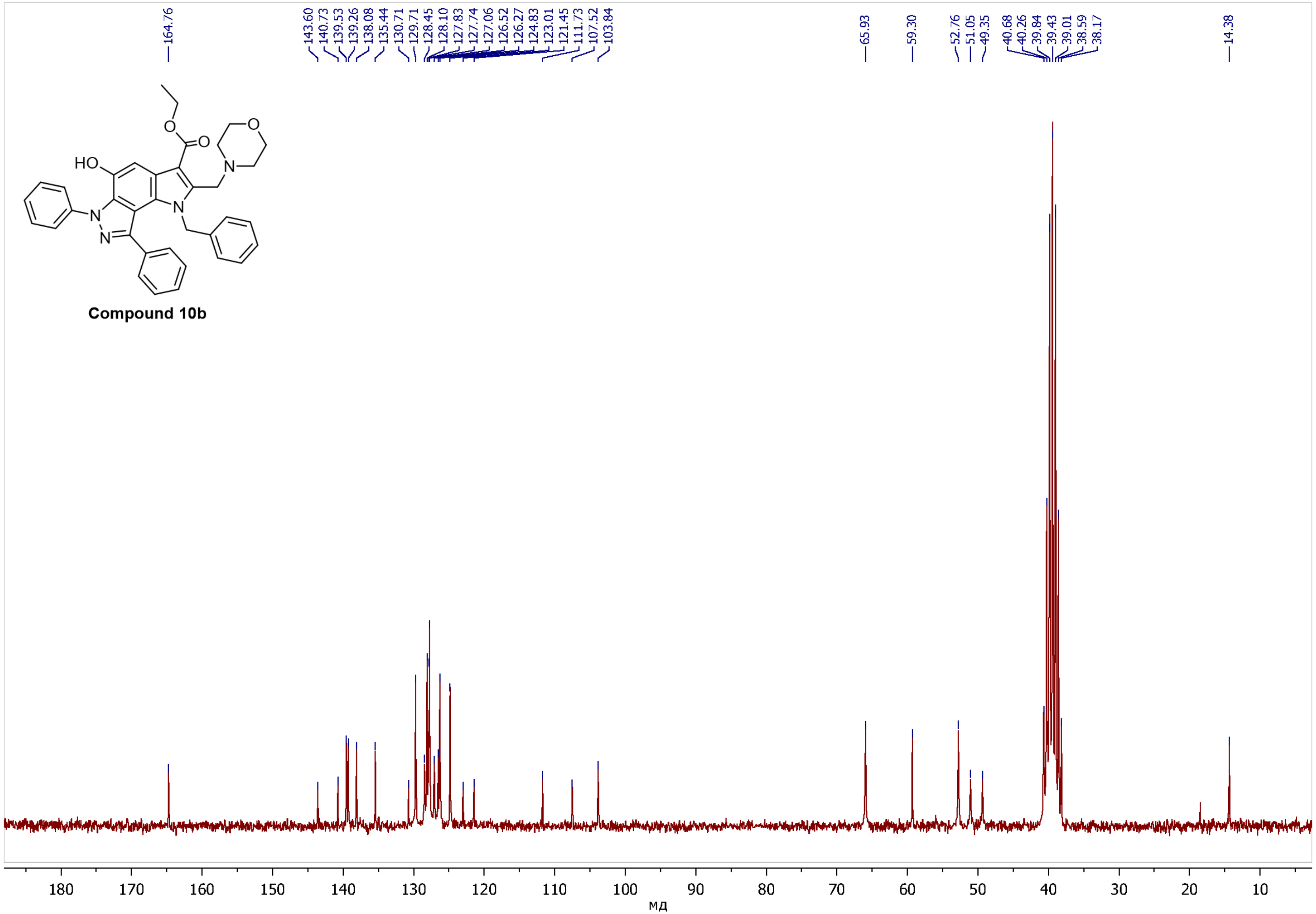

^{1}H NMR spectrum (200 MHz, DMSO-d$_6$) of compound **11a**

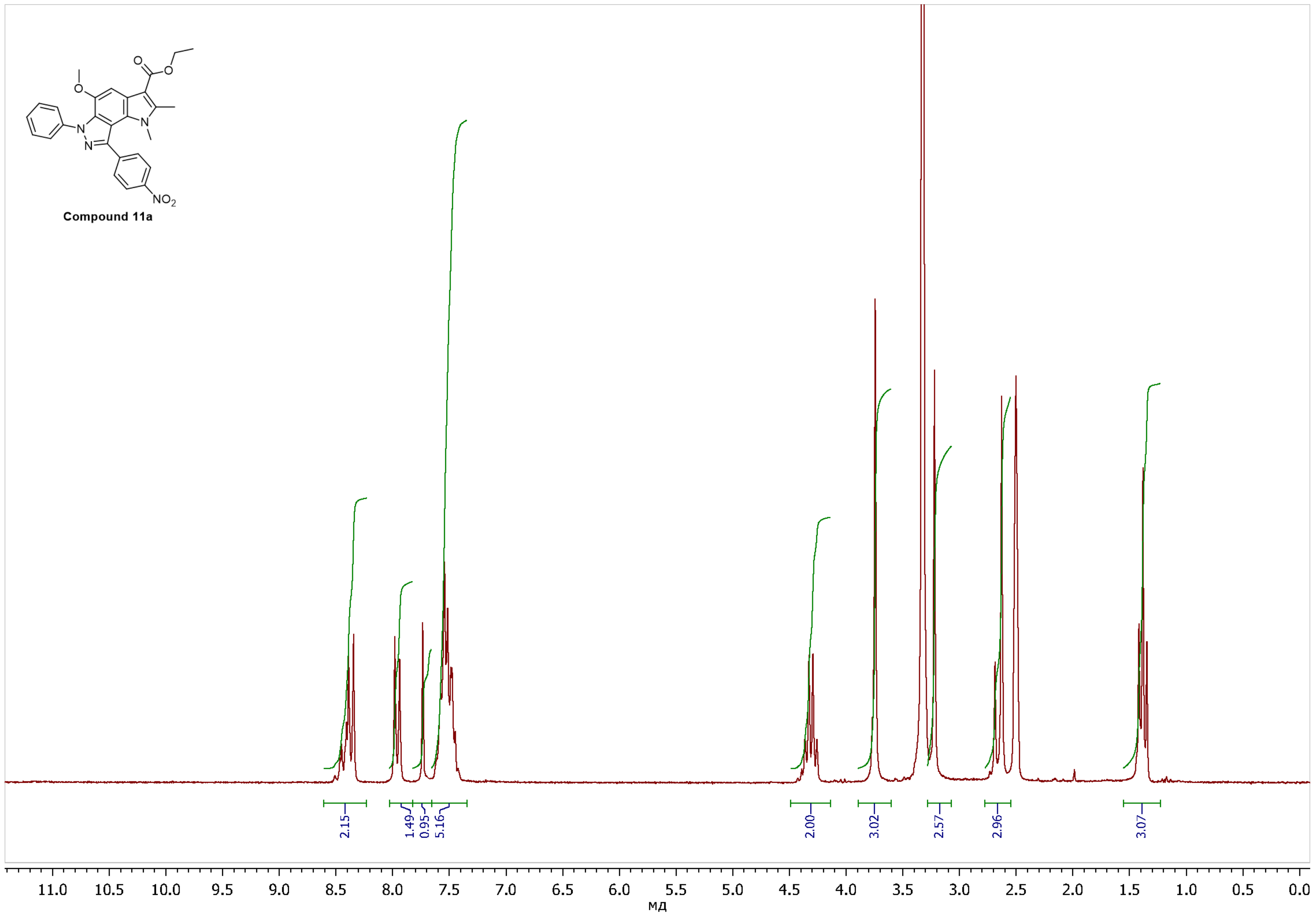

^{13}C NMR spectrum (50 MHz, DMSO-d$_6$) of compound **11a**

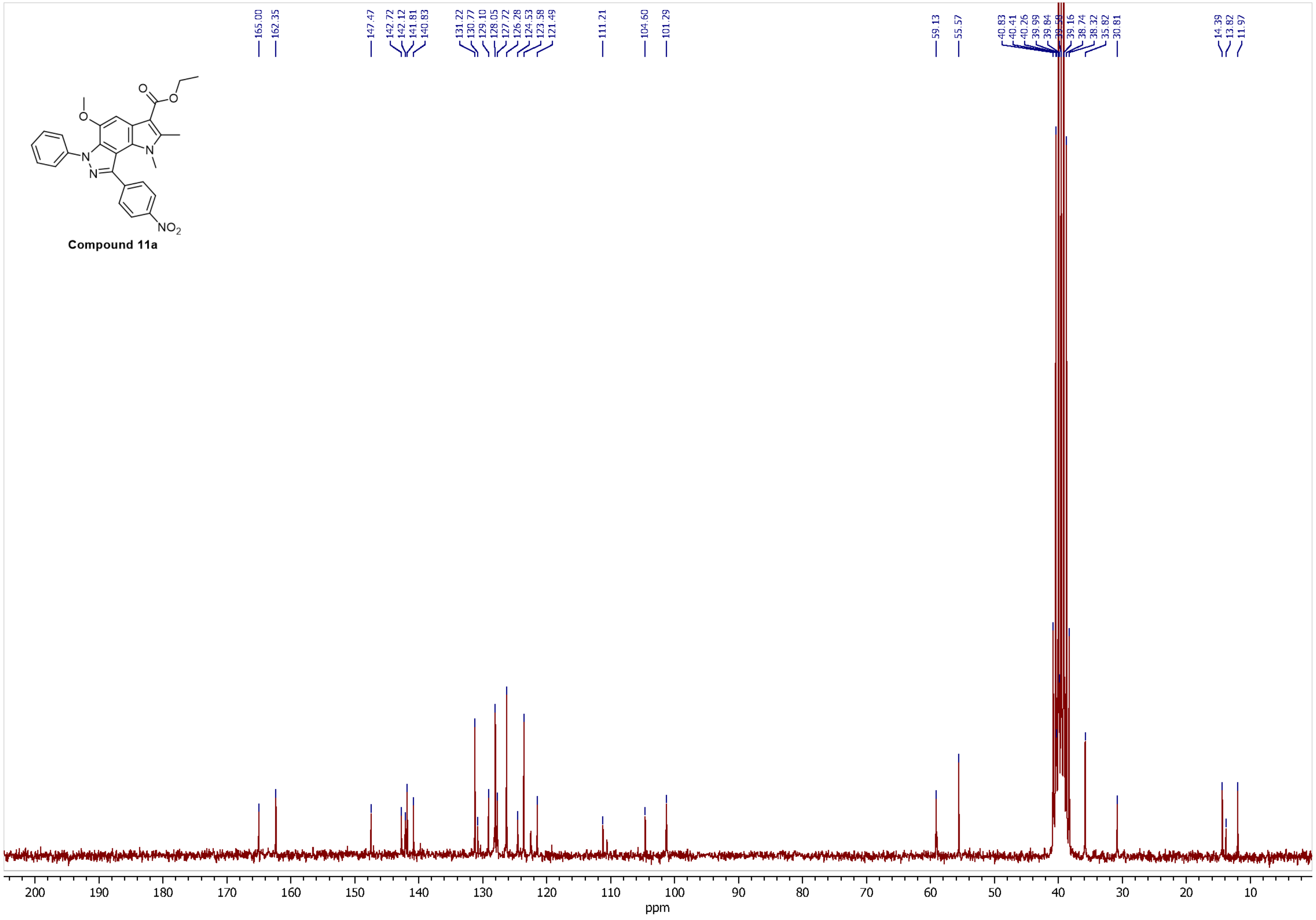

^{1}H NMR spectrum (200 MHz, DMSO-d_6) of compound **11b**

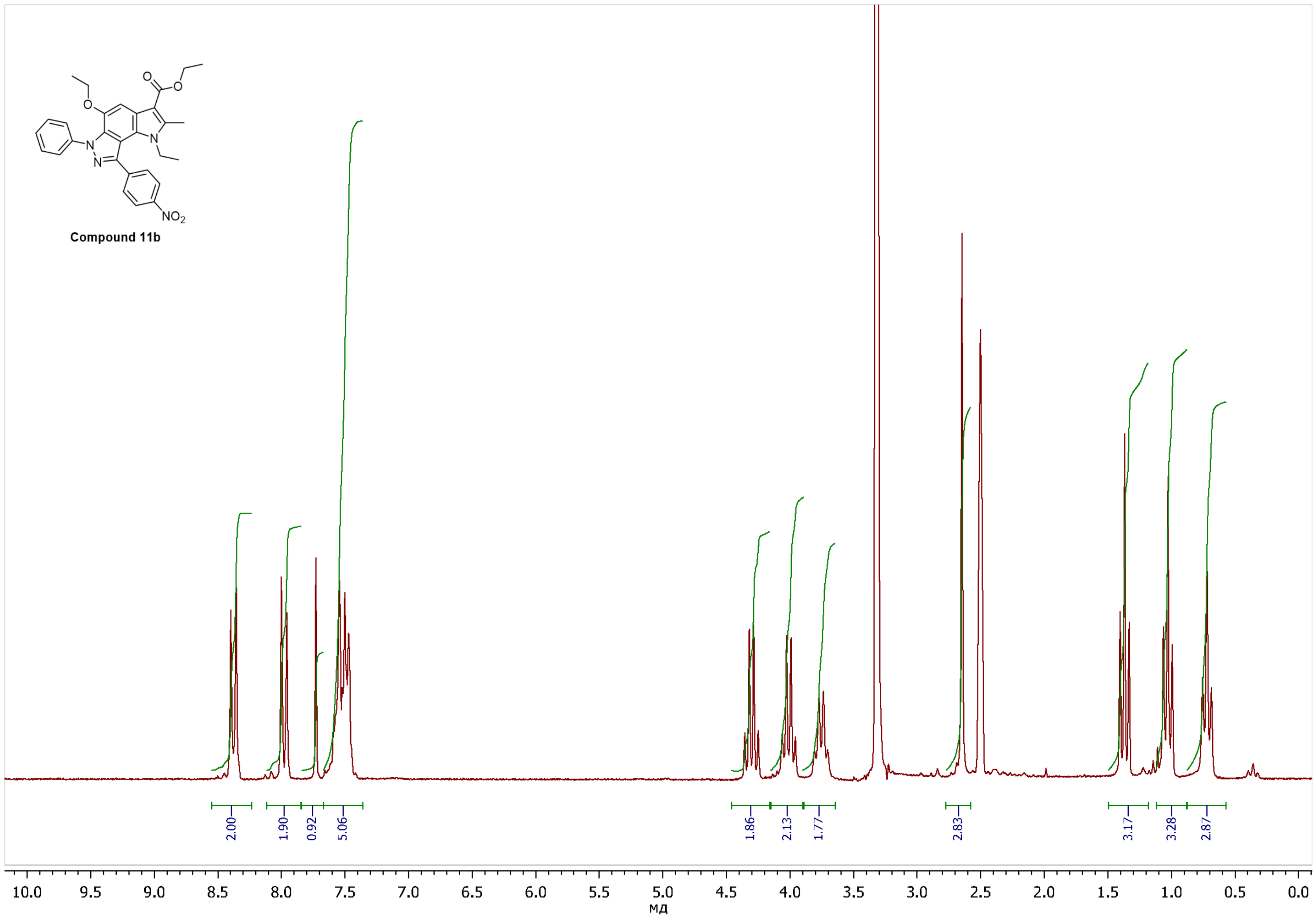

^{13}C NMR spectrum (50 MHz, DMSO-d$_6$) of compound **11b**

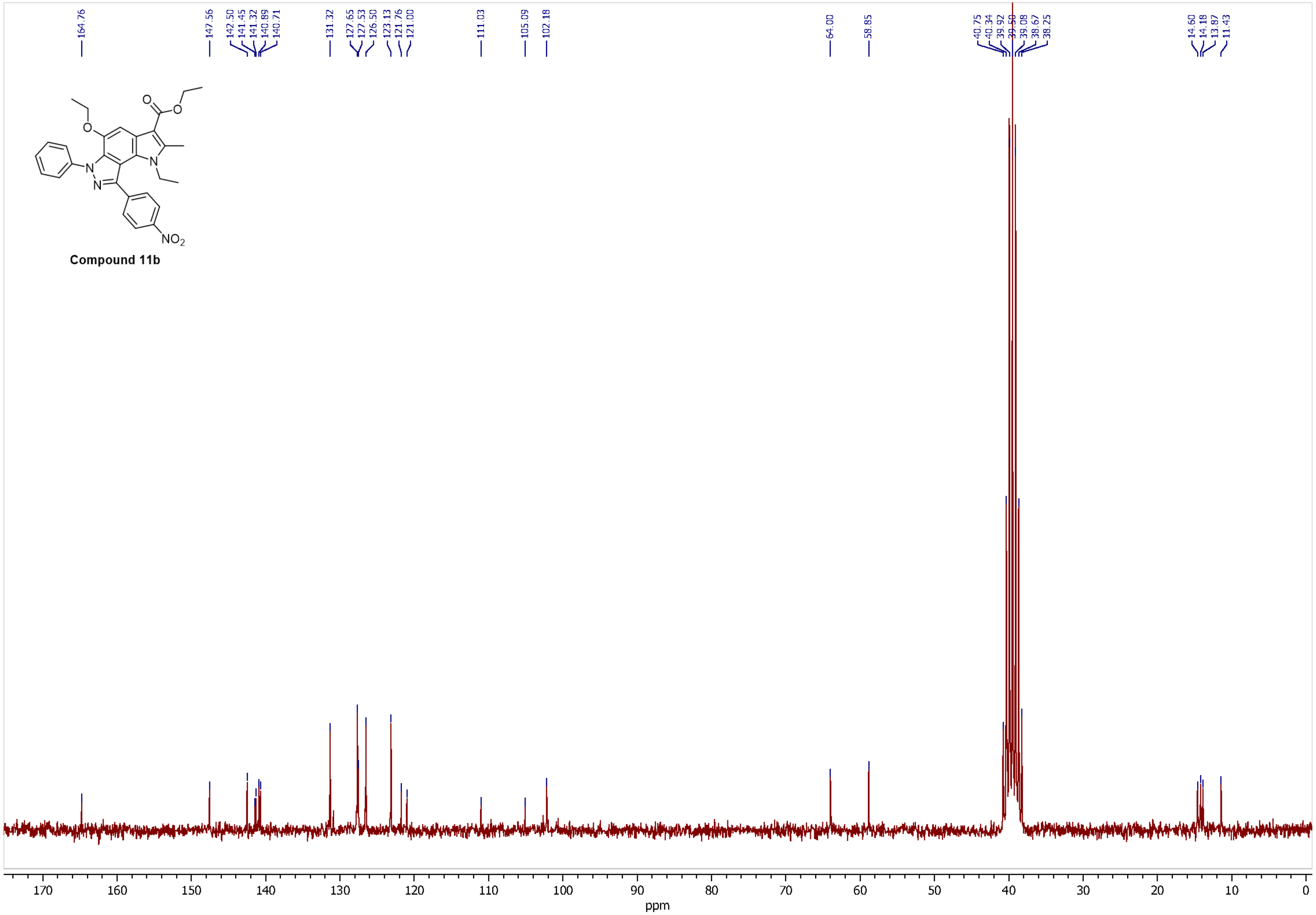

^{1}H NMR spectrum (500 MHz, DMSO-d_6) of compound **11c**

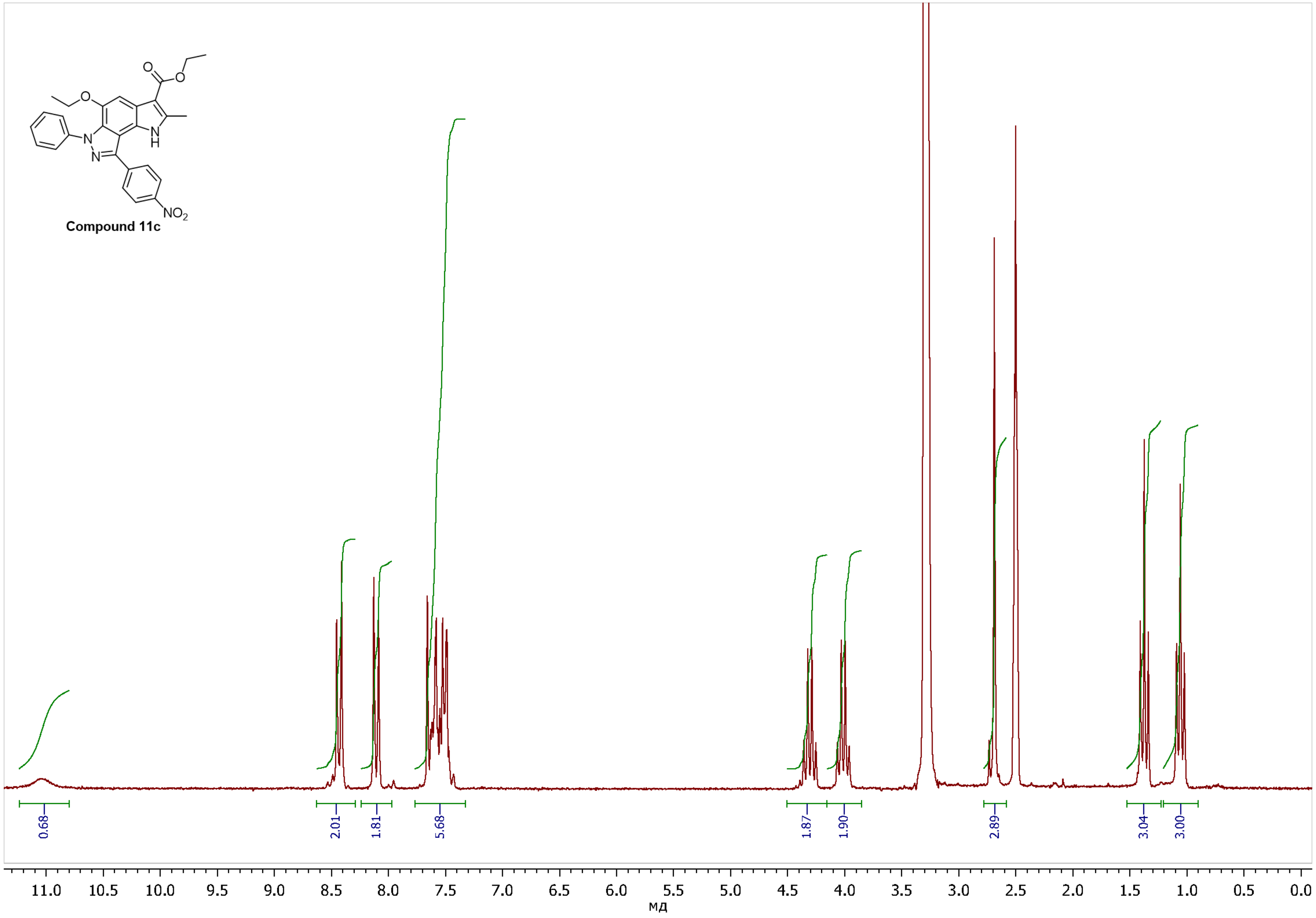

^{13}C NMR spectrum (126 MHz, DMSO-d$_6$) of compound **11c**

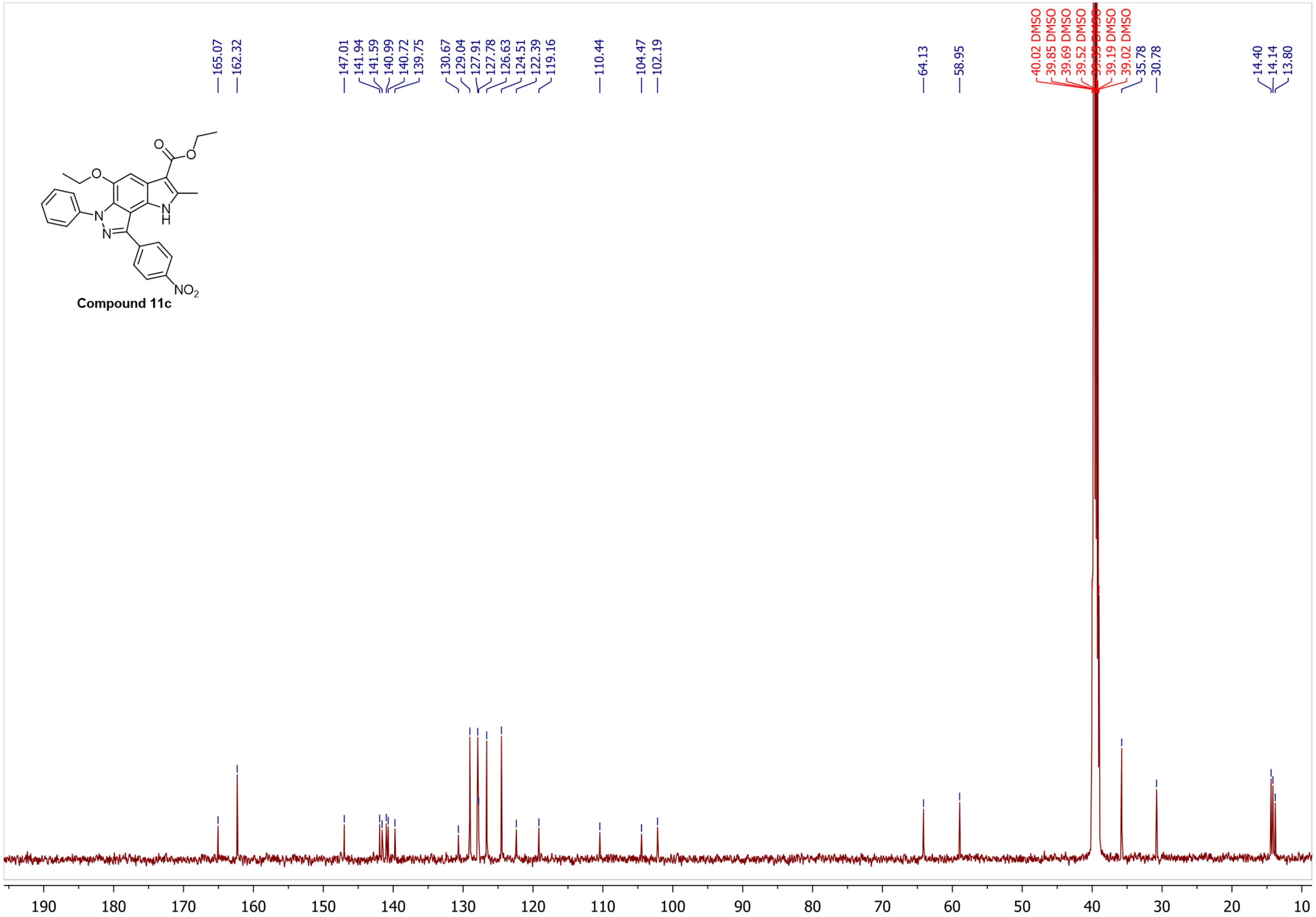

^{1}H NMR spectrum (200 MHz, DMSO-d_6) of compound **11d**

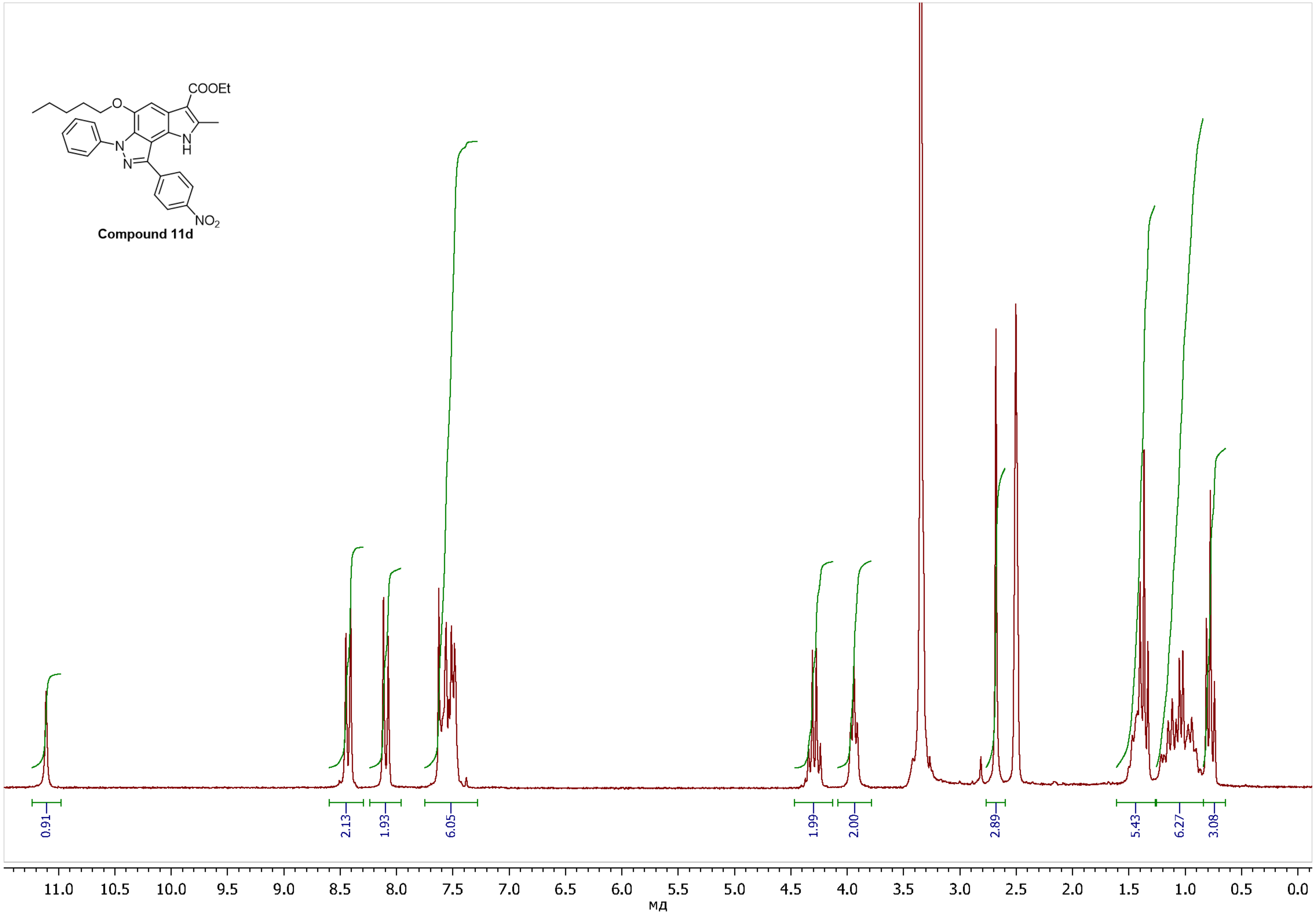

^{13}C NMR spectrum (50 MHz, DMSO-d_6) of compound **11d**

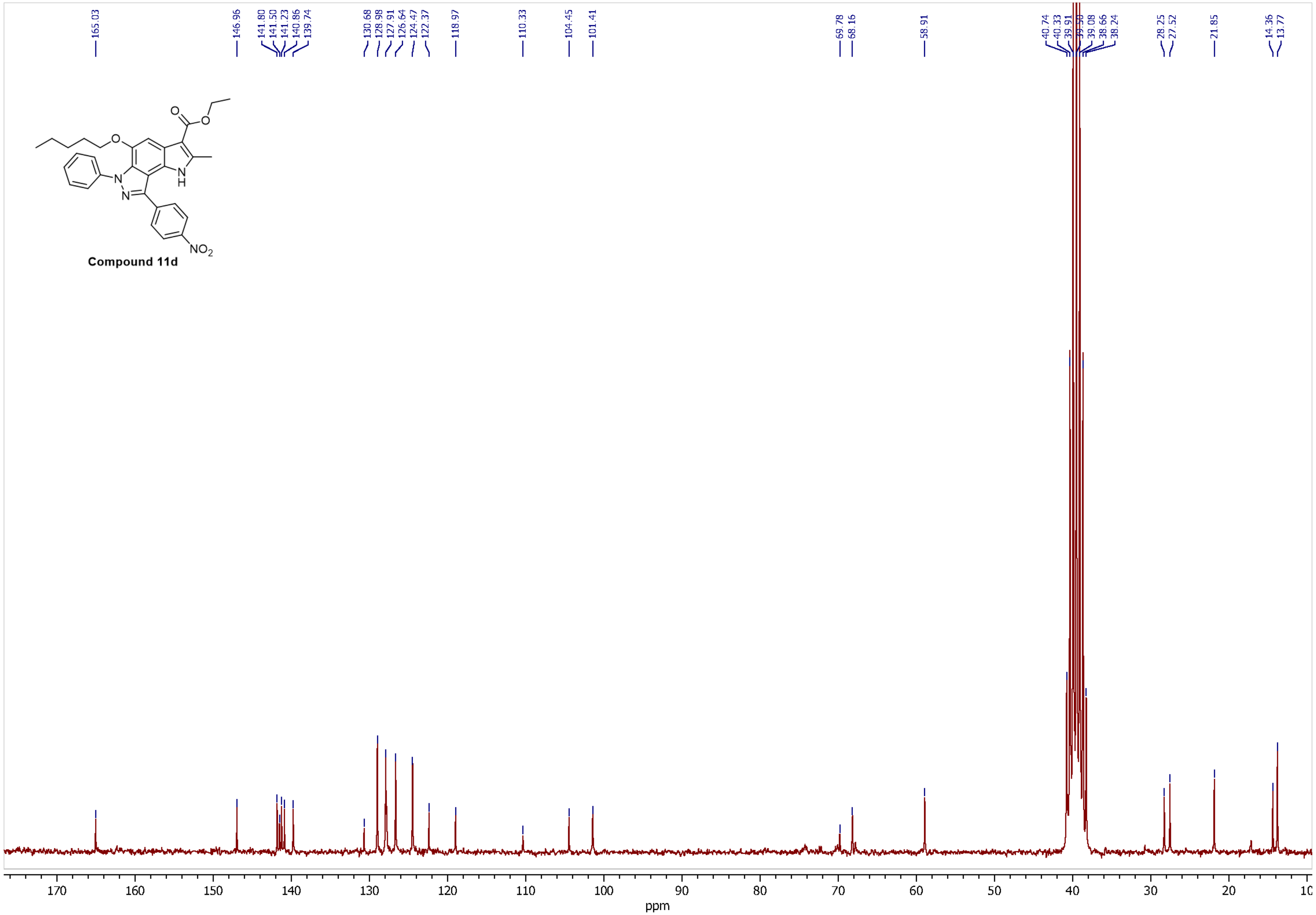

^1H NMR spectrum (200 MHz, DMSO-d_6) of compound **11e**

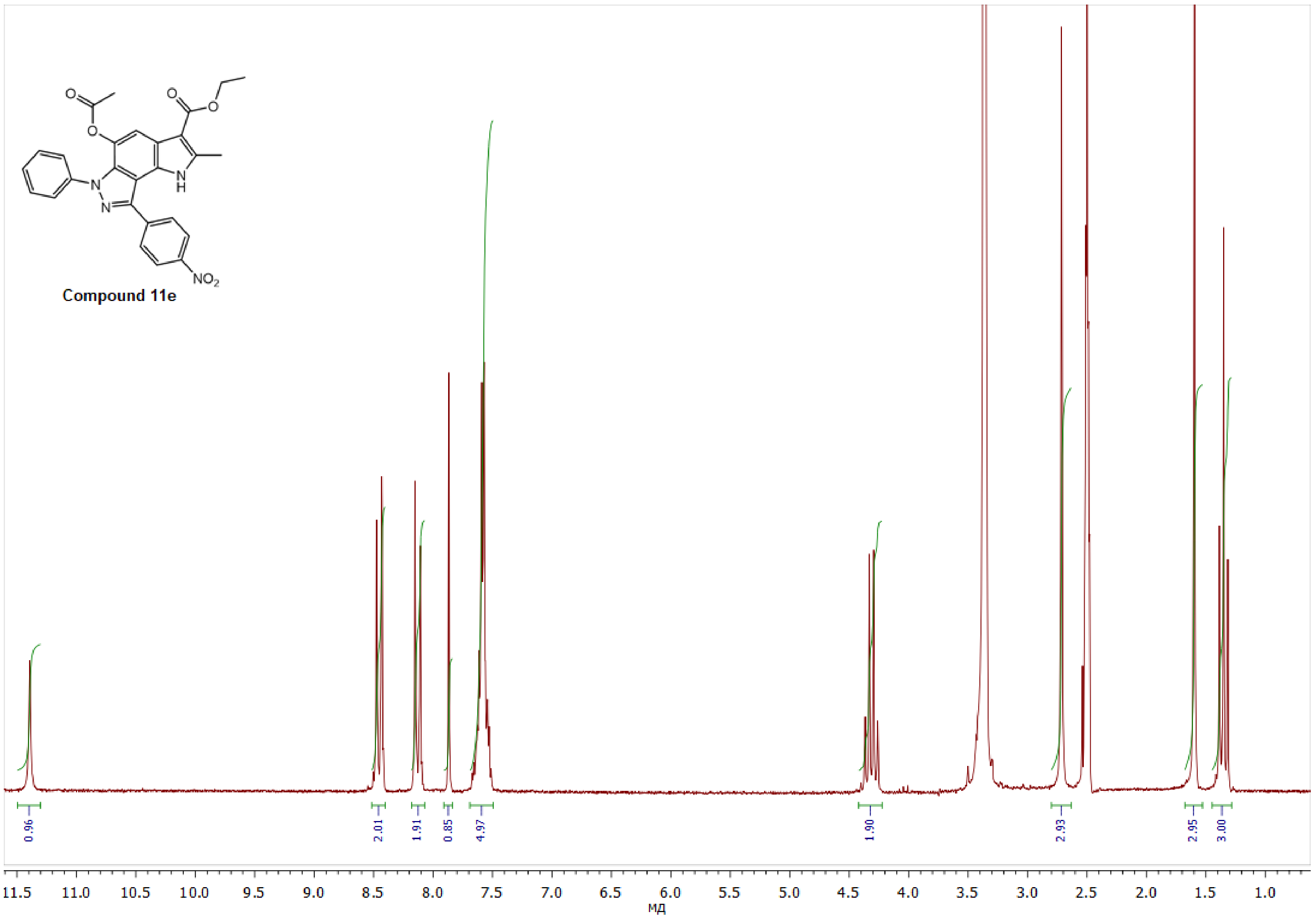

^{13}C NMR spectrum (50 MHz, DMSO-d_6) of compound **11e**

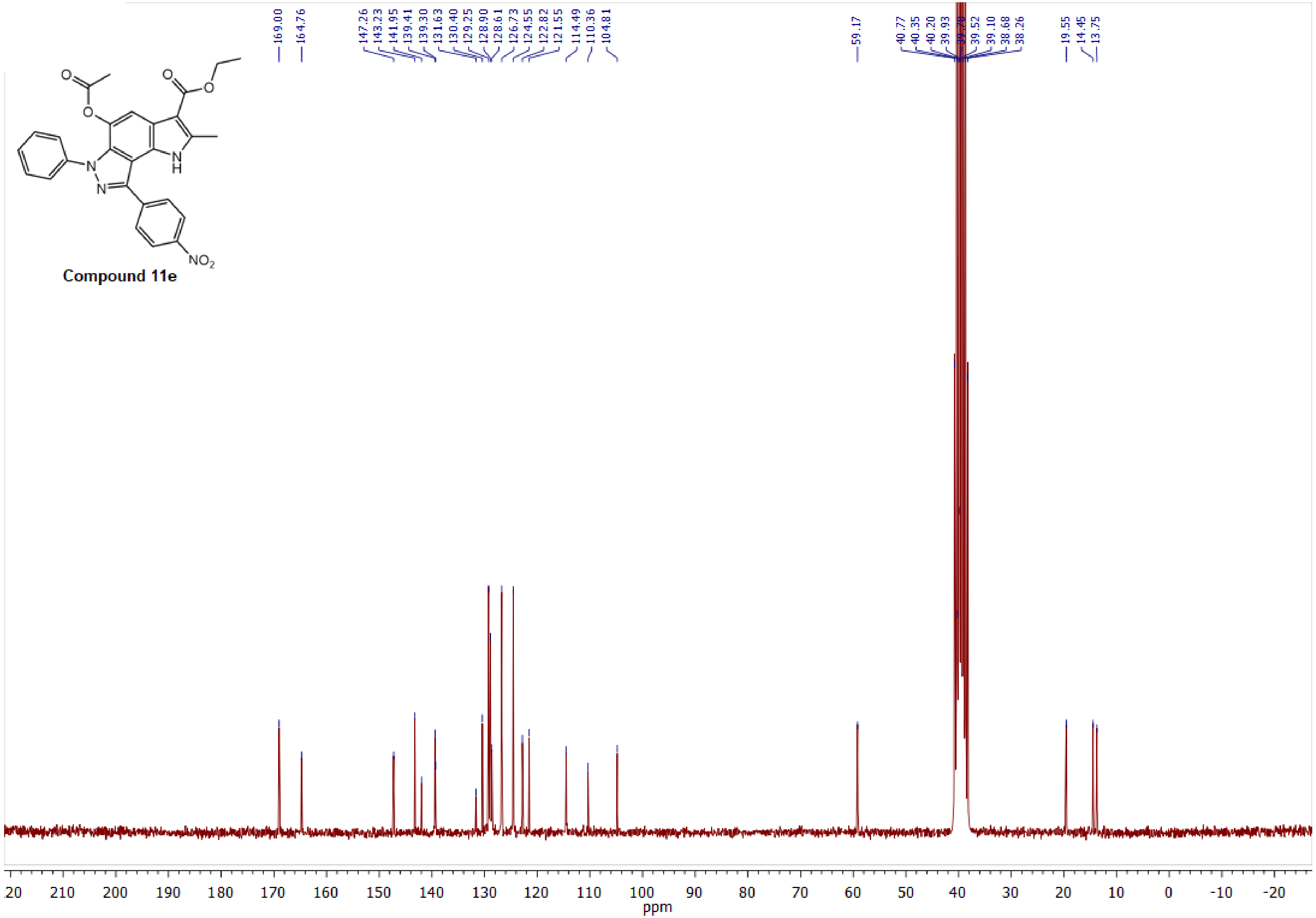

^{1}H NMR spectrum (200 MHz, DMSO-d_6) of compound **12**

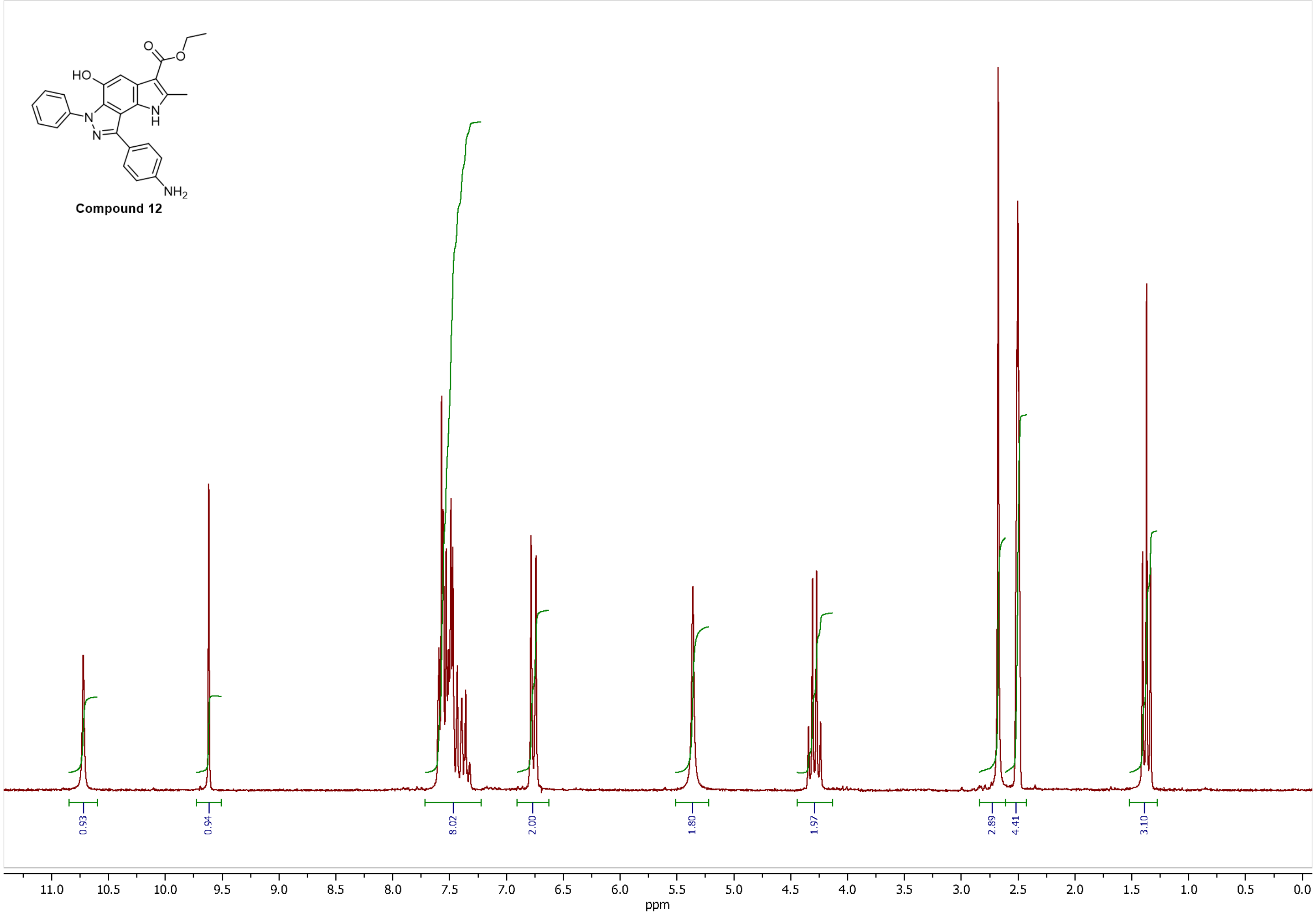

^{13}C NMR spectrum (50 MHz, DMSO-d$_6$) of compound **12**

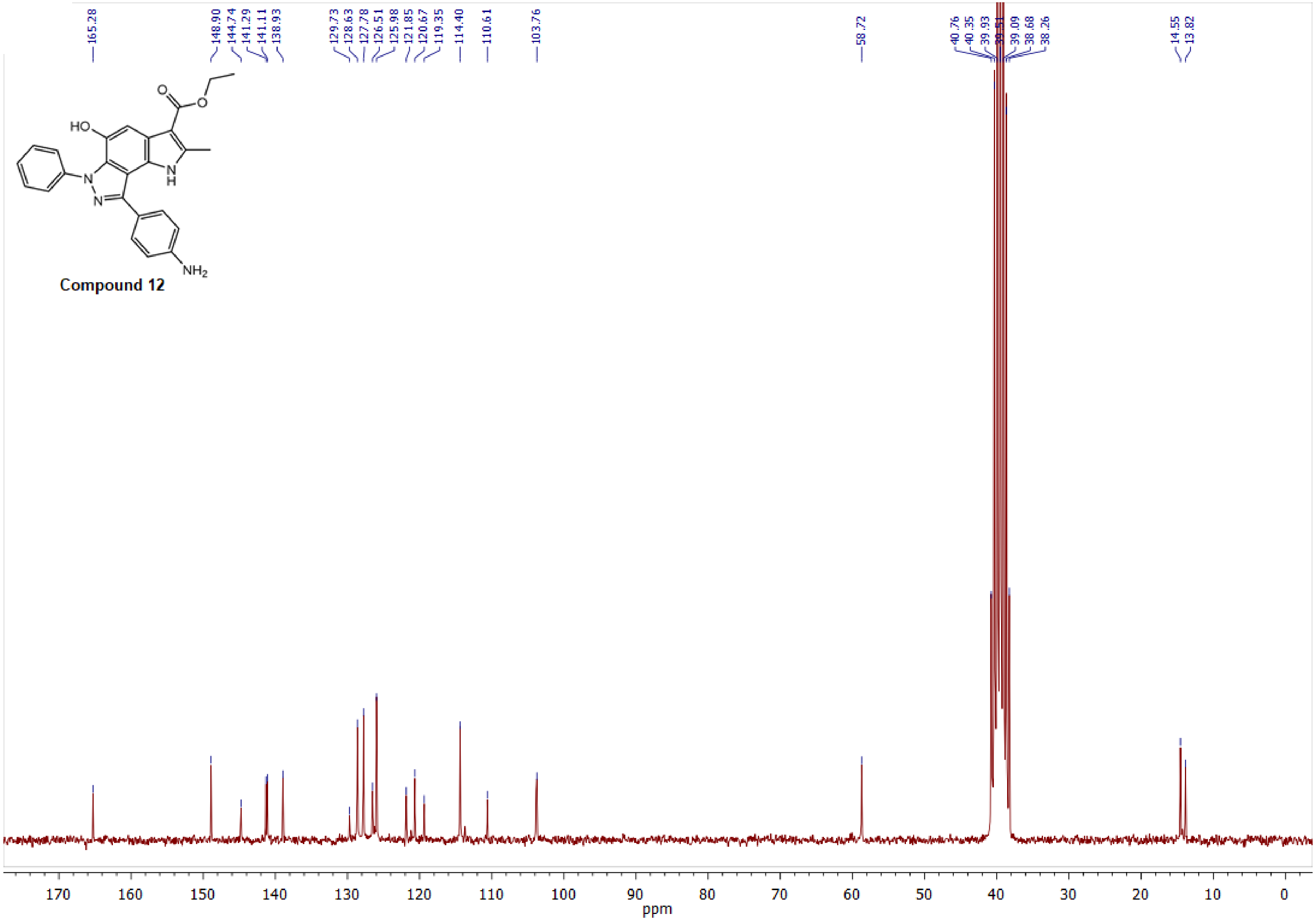

^{1}H NMR spectrum (200 MHz, DMSO-d6) of compound **13**

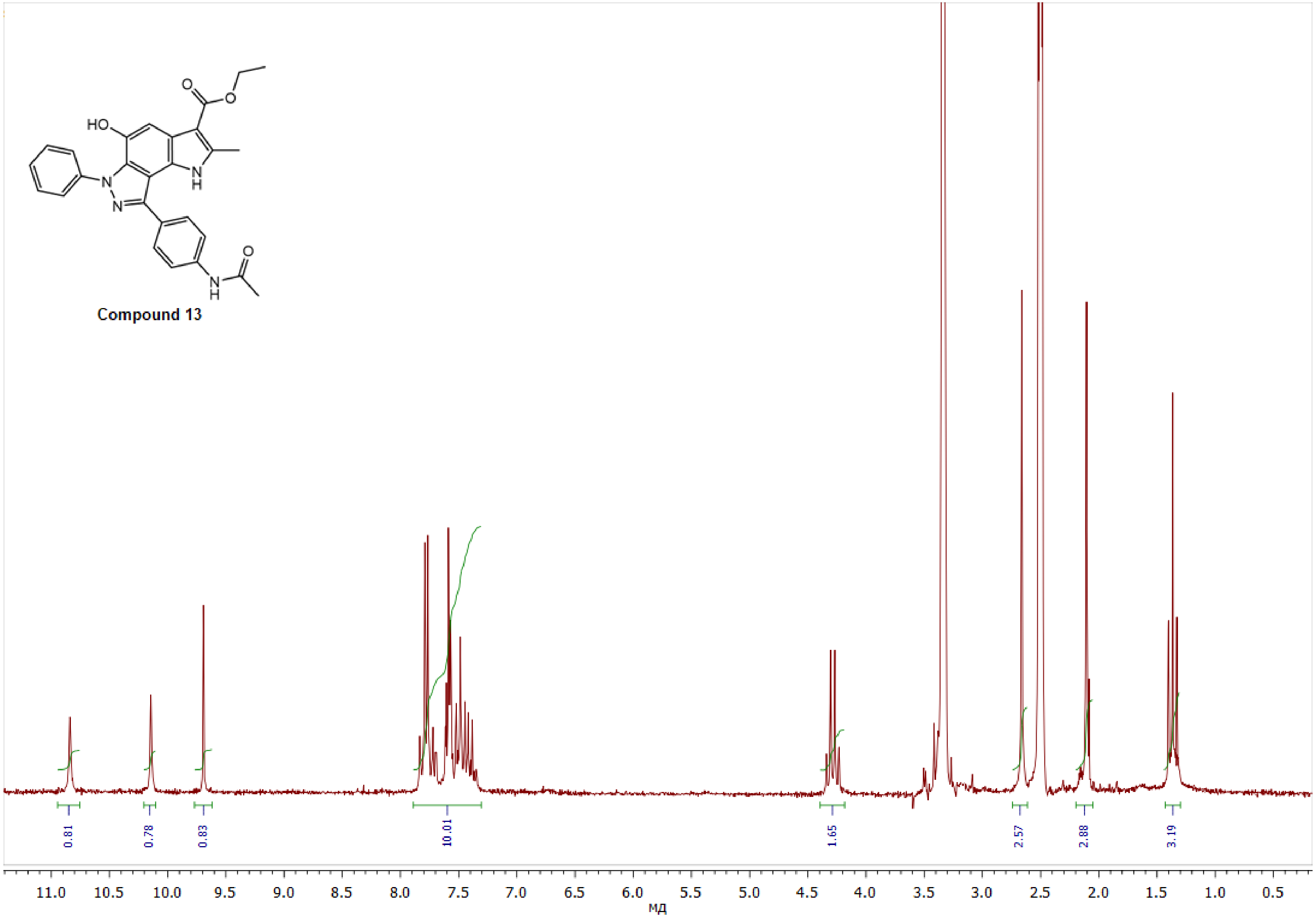

^{13}C NMR spectrum (50 MHz, DMSO-d_6) of compound **13**

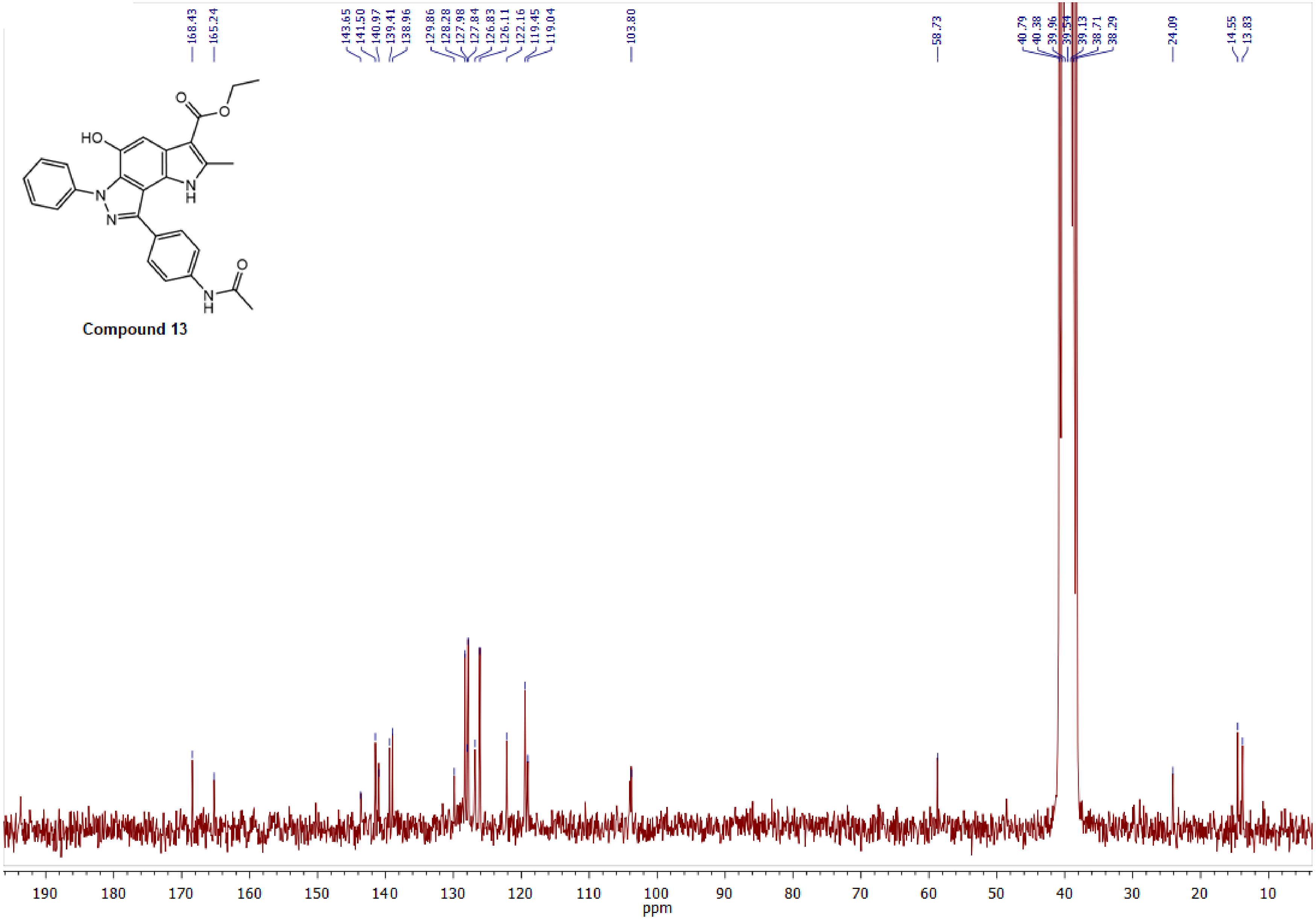

^{1}H NMR spectrum (200 MHz, DMSO-d$_6$) of compound **14**

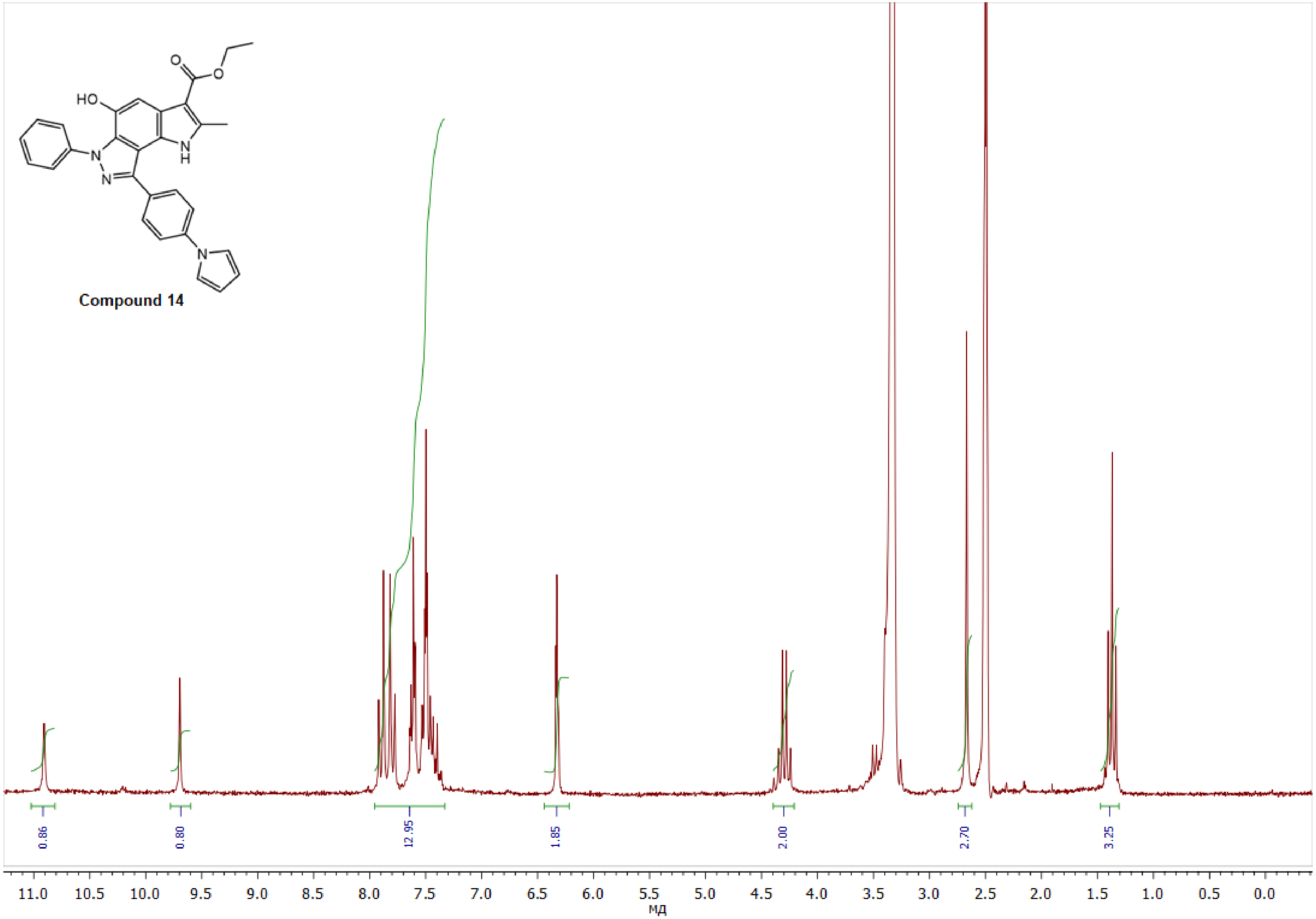

^{13}C NMR spectrum (50 MHz, DMSO-d_6) of compound **14**

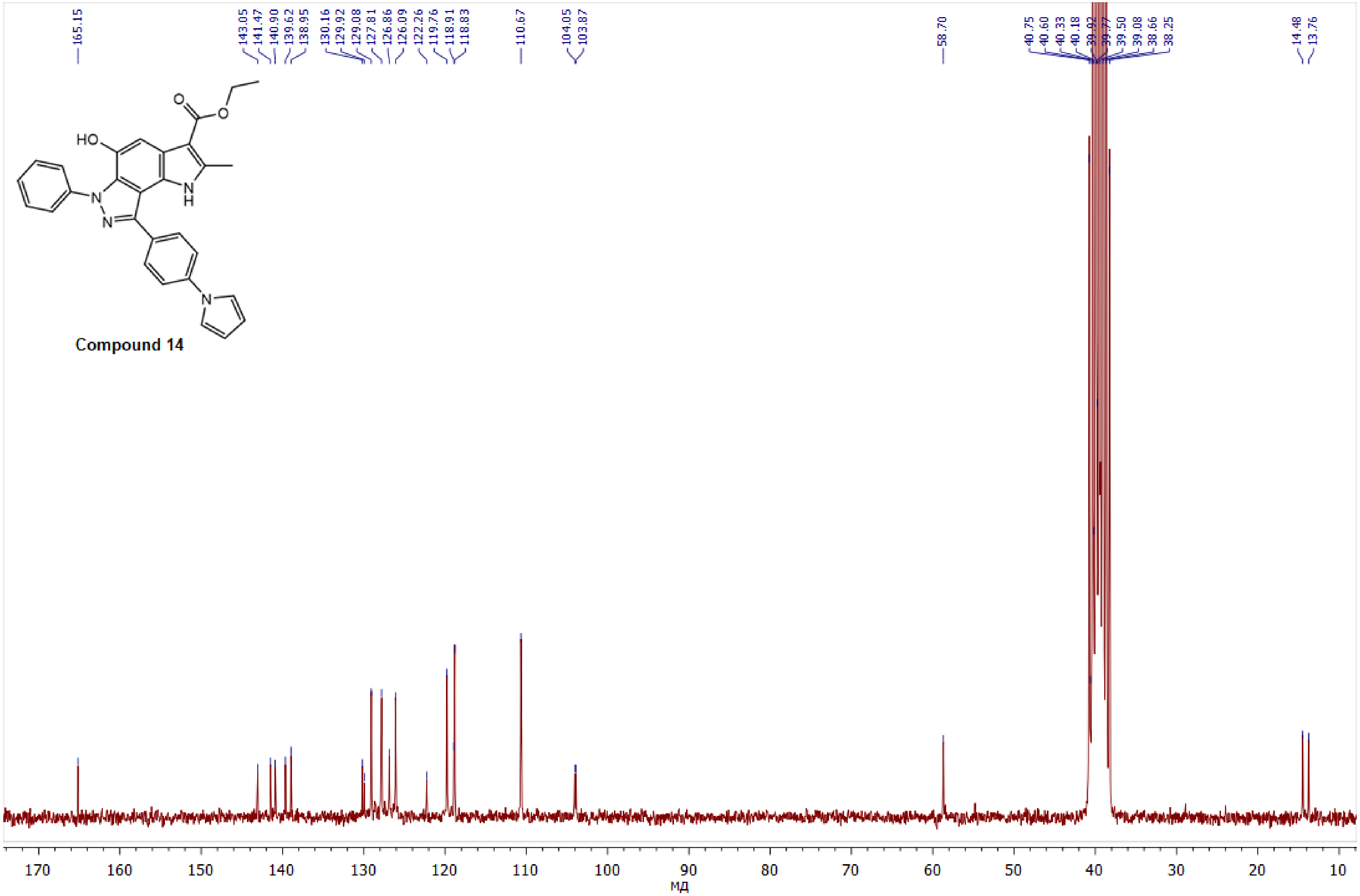

Representative HPLC Traces

HPLC purity of 5-(acetyloxy)-3-ethoxycarbonyl-1,2-dimethyl-6,8-diphenyl-pyrrolo[2,3-*e*]indazole **7a**

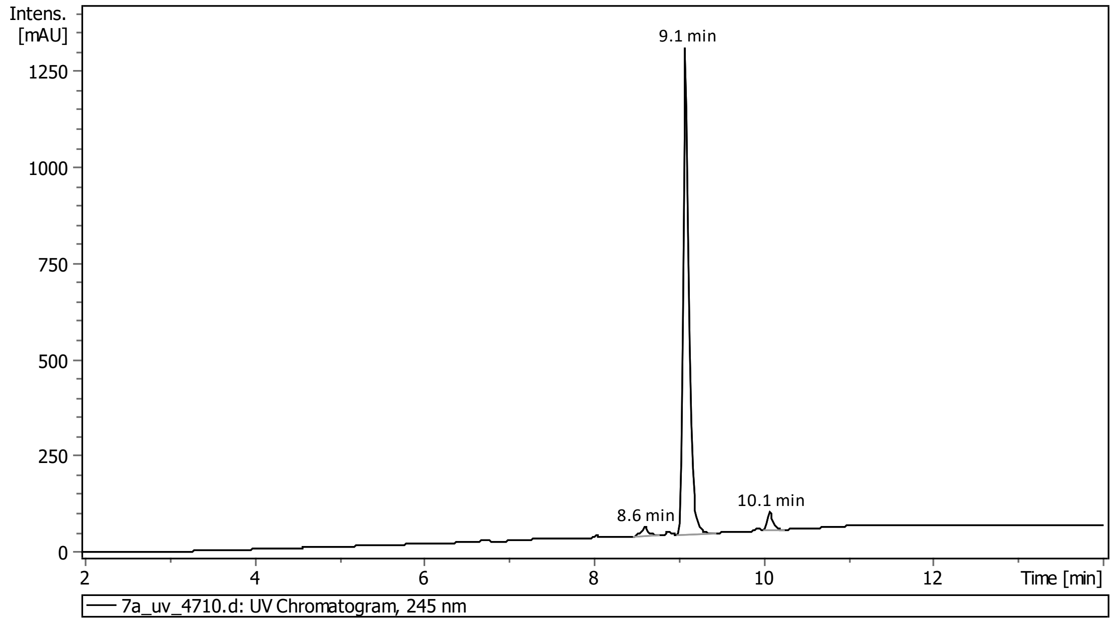

Peak Results

#	RT [min]	Area	S/N	Area Total [%]
1	8.6	122.77	496.4	1.87
2	9.1	6221.53	27653.9	95.10
3	10.1	206.19	1051.6	3.03

HPLC purity of 5-hydroxy-3-ethoxycarbonyl-1-methyl-6,8-diphenyl-2-(piperidin-1-ylmethyl)-pyrrolo[2,3-*e*]indazole hydrochloride **10a**

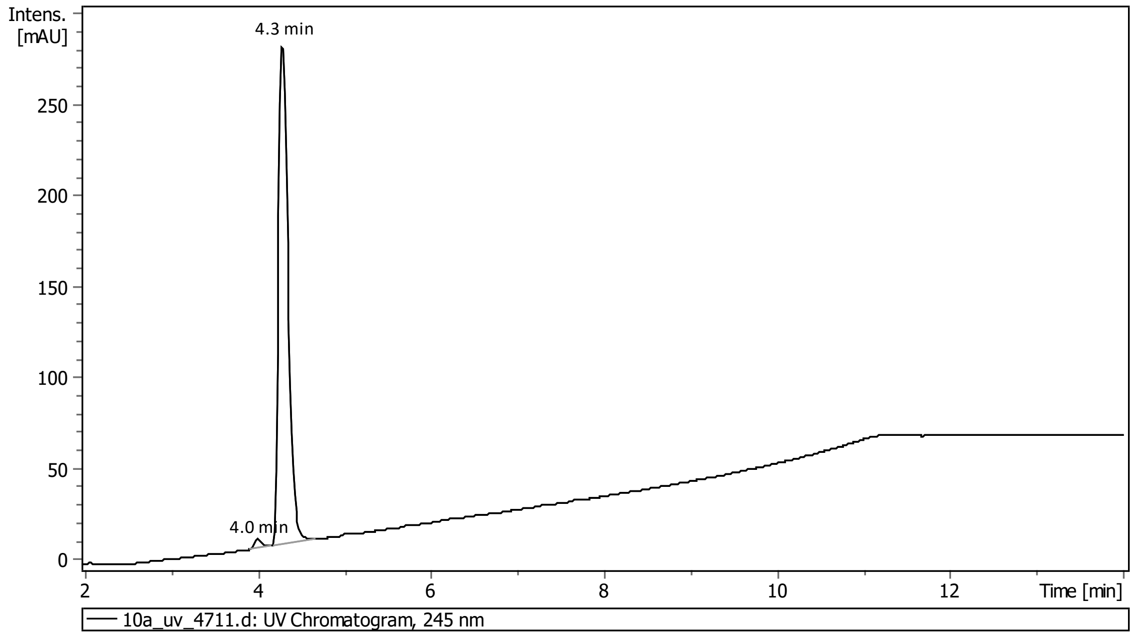

Peak Results

#	RT [min]	Area	S/N	Area Total [%]
1	4.0	28.986	113.4	1.34
2	4.3	2130.749	6323.0	98.66

HPLC purity of 5-methoxy-3-ethoxycarbonyl-1,2-dimethyl-8-(4-nitrophenyl)-6-phenyl-pyrrolo[2,3-*e*]indazole **11a**

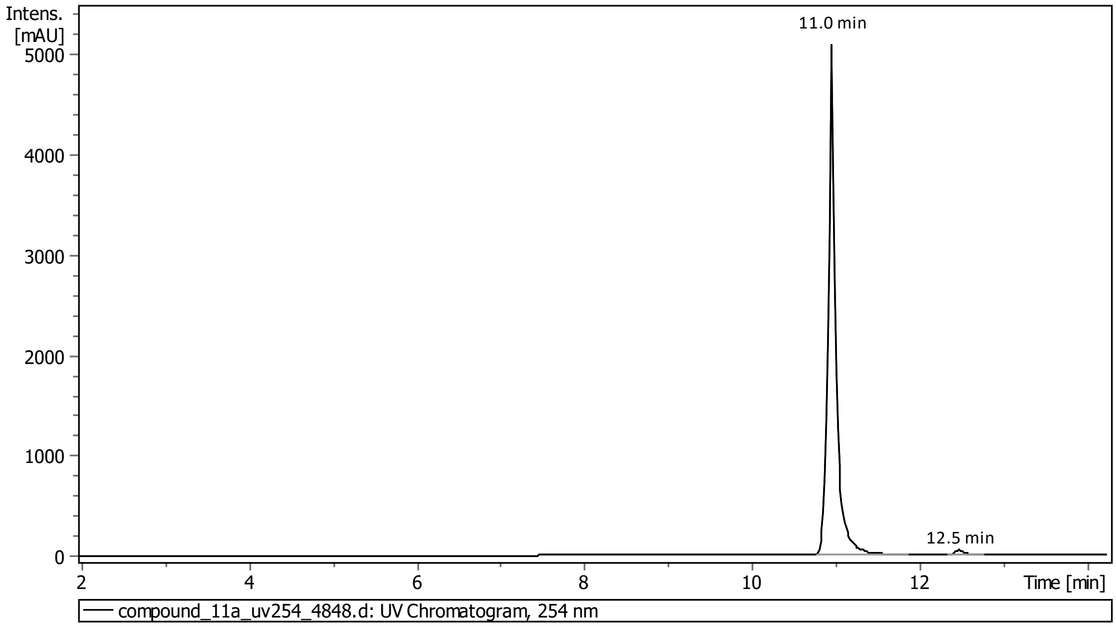

Peak Results

#	RT [min]	Area	S/N	Area Total [%]
1	11.0	32367.17	140790.3	99.08
2	12.5	301.01	1245.0	0.92

HPLC purity of 8-(4-aminophenyl)-5-hydroxy-3-ethoxycarbonyl-2-methyl-6-phenyl-pyrrolo[2,3-*e*]indazole **12**

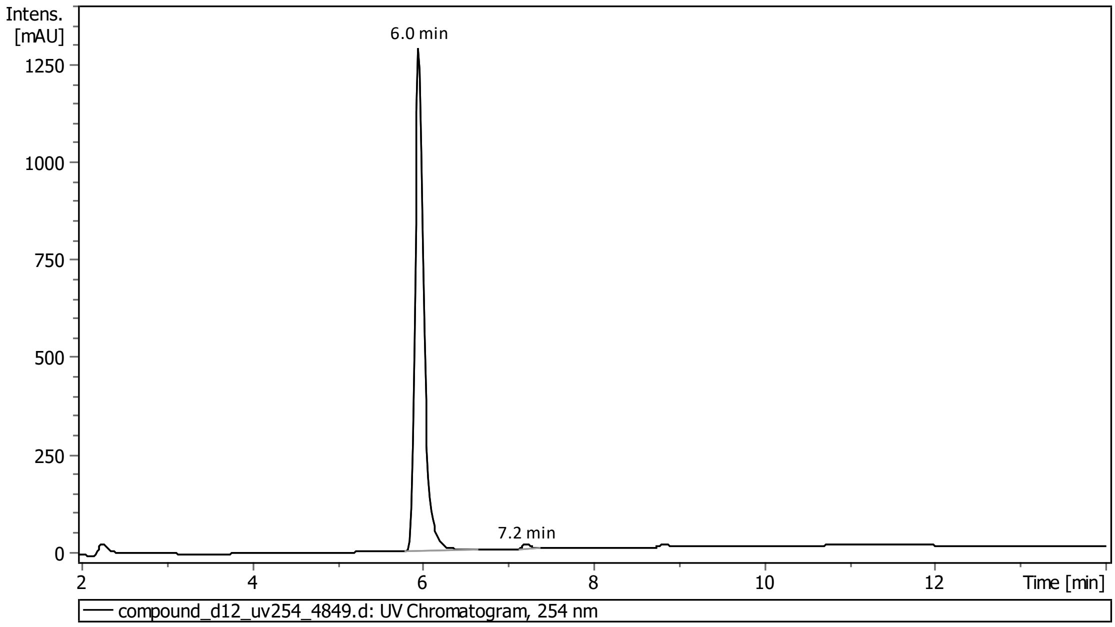

Peak Results

#	RT [min]	Area	S/N	Area Total [%]
1	6.0	9305.151	25315.0	99.17
2	7.2	77.765	222.8	0.83

Representative IR Spectra

IR spectrum (KBr) for compound 6h

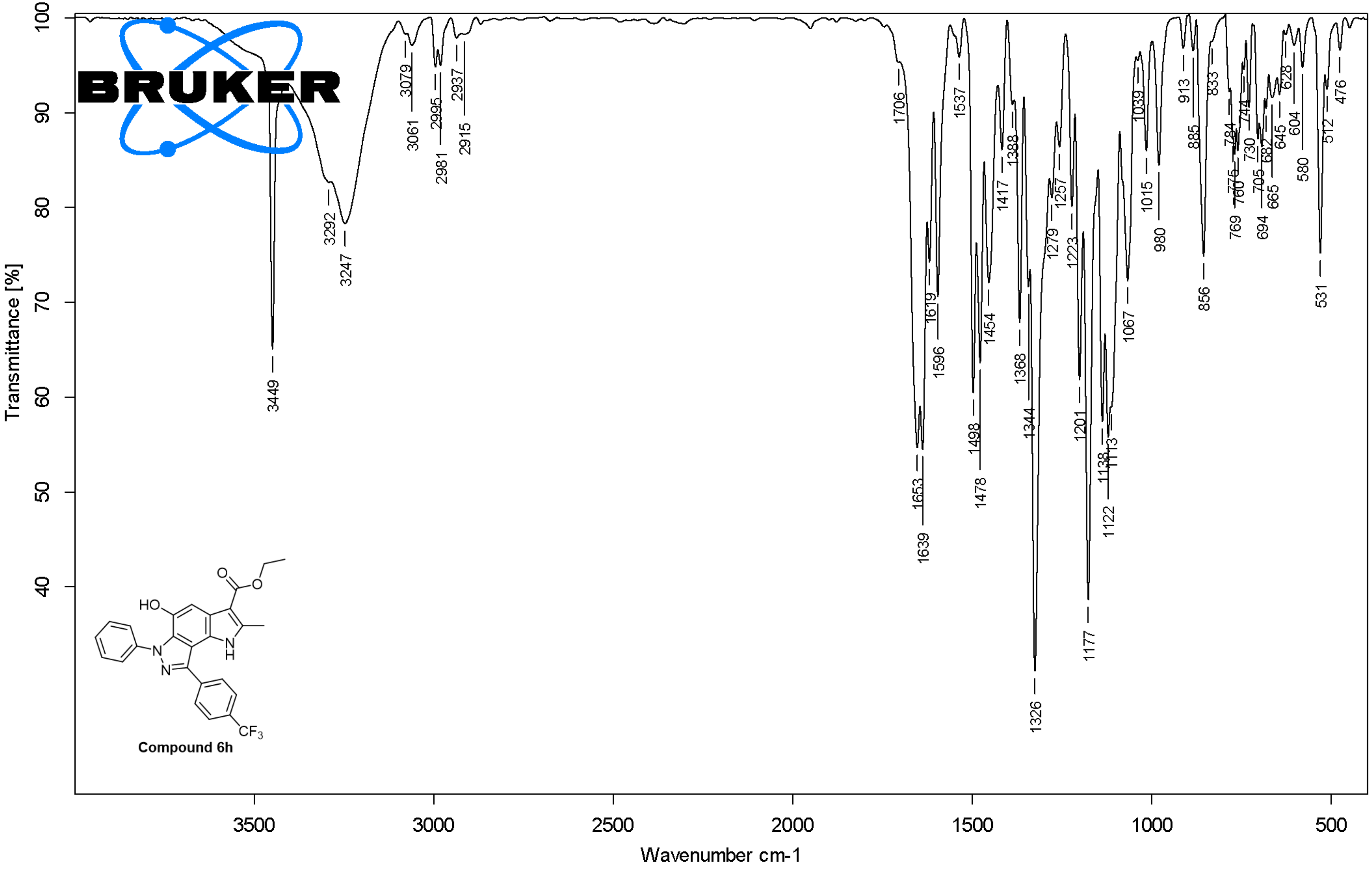

IR spectrum (KBr) for compound 12

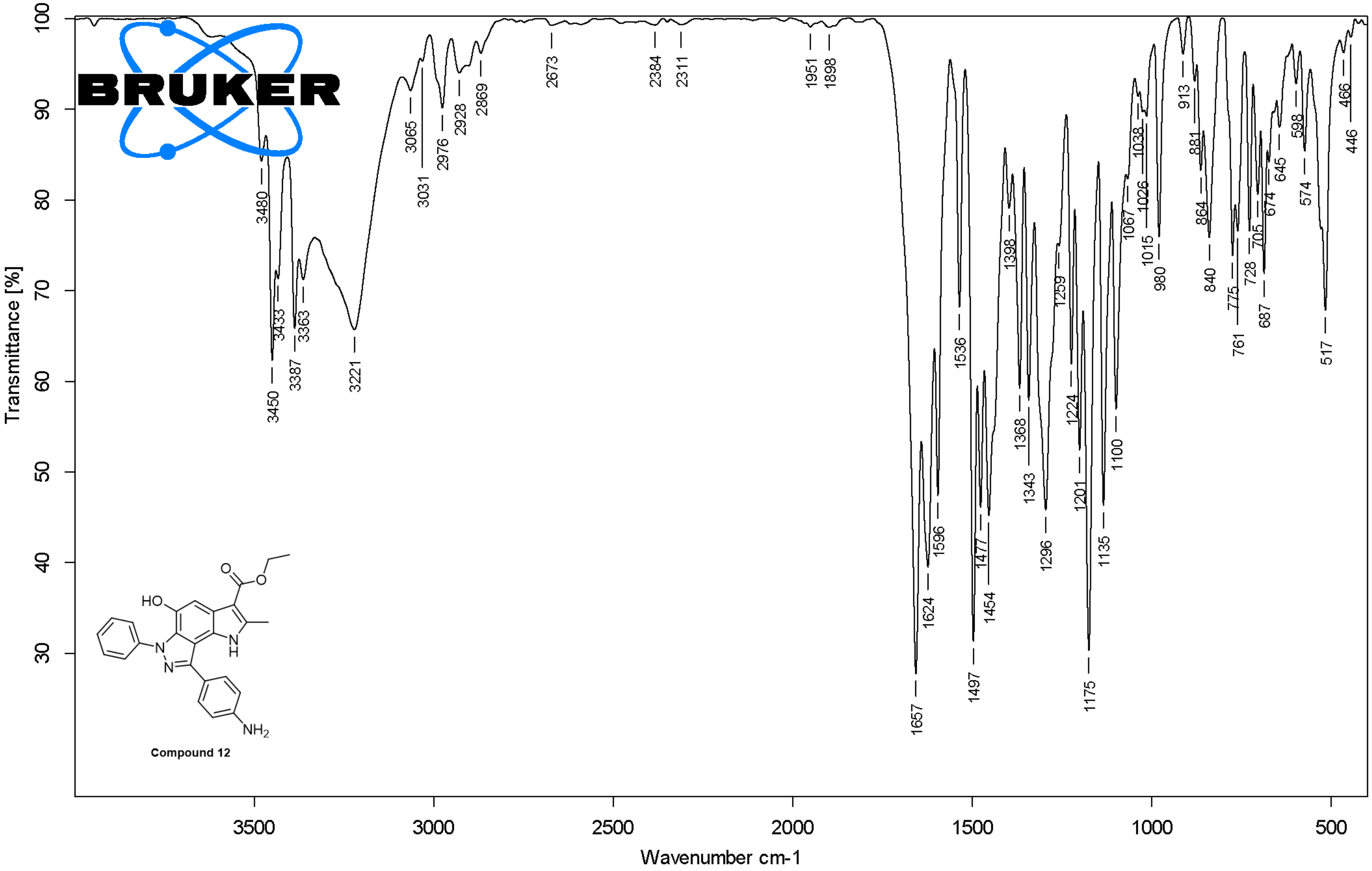

References

1. Safaei-Ghomi J, Masoomi R. Microwave-assisted synthesis of fulleropyrazolines/ fulleroisoxazolines mediated by (diacetoxyiodo)benzene: a rapid and green procedure. RSC Adv. 2014; 4: 2954-2960. doi: 10.1039/c3ra44567d.
2. Lyubchanskaya VM, Alekseeva LM, Savina SA, Granik GV. Indazolequinones in the Nenitzescu reaction. Synthesis of pyrrolo[2,3-*e*]- and furo[2,3-*e*]indazoles. Chem Heterocycl Compd. 2000; 36: 1276-1283. doi: 10.1023/A:1017515300244.
3. Sheldrick GM. A short history of SHELX. Acta Crystallogr A. 2008; 64(Pt 1): 112-122. doi: 10.1107/S0108767307043930.
4. Zhukov SG, Chernyshev VV, Babaev EV, Sonneveld EJ, Schenk H. Application of simulated annealing approach for structure solution of molecular crystals from X-ray laboratory powder data. Z. Kristallogr. Cryst. Mater. 2009; 216: 5-9. doi: 10.1524/zkri.216.1.5.18998.
5. Zlokazov VB, Chernyshev VV. MRIA - a program for a full profile analysis of powder multiphase neutron-diffraction time-of-flight (direct and Fourier) spectra. J. Appl. Crystallogr. 1992, 25, 447-451. doi: 10.1107/S0021889891013122.
6. Tafeenko VA, Chernyshev VV, Yatsenko AV, Makarov VA, Sonneveld EJ, Peschar R, Schenk H. Intermolecular -CH3...O2N-- contacts in two polymorphic modifications of (1E)-N'-[(E)-2-cyano-1-(dimethylamino)-2-nitrovinyl]-N,N-dimethylethanimidamide. Acta Crystallogr B. 2003; 59:492-497. doi: 10.1107/s0108768103010231.
7. Chernyshev VV, Stephens PW, Yatsenko AV, Ryabova OB, Makarov VA. Structural characterization of prazosin hydrochloride and prazosin free base. J Pharm Sci. 2004; 93: 3090-3095. doi: 10.1002/jps.20204.
8. Ahtee M, Nurmela M, Suortti P, Järvinen M. Correction for preferred orientation in Rietveld refinement. J. Appl. Cryst. 1989, 22; 261-268. doi: 10.1107/S0021889889000725.
9. Järvinen M. Application of symmetrized harmonics expansion to correction of the preferred orientation effect. J. Appl. Cryst. 1993; 26: 525-531. doi: 10.1107/S0021889893001219.
10. Walther E, Xu Z, Richter M, Kirchmair J, Grienke U, Rollinger JM, Krumbholz A, Saluz HP, Pfister W, Sauerbrei A, Schmidtke M. Dual acting neuraminidase inhibitors open new opportunities to disrupt the lethal synergism between *Streptococcus pneumoniae* and influenza virus. Front Microbiol. 2016; 7: 357. doi: 10.3389/fmicb.2016.0035.
11. Schmidtke M, Schnittler U, Jahn B, Dahse H, Stelzner A. A rapid assay for evaluation of antiviral activity against Coxsackie. virus B3, influenza virus A, and herpes simplex virus type 1. J Virol Methods. 2001; 9(1-2): 133-143. doi: 10.1016/s0166-0934(01)00305-6.
12. Richter M, Schumann L, Walther E, Hoffmann A, Braun H, Grienke U, Rollinger JM, von Grafenstein S, Liedl KR, Kirchmair J, Wutzler P, Sauerbrei A, Schmidtke M. Complementary assays helping to overcome challenges for identifying neuraminidase inhibitors. Future Virol. 2015; 10(2): 77-88. doi: 10.2217/fvl.14.97.

13. Walther E, Richter M, Xu Z, Kramer C, von Grafenstein S, Kirchmair J, Grienke U, Rollinger JM, Liedl KR, Slevogt H, Sauerbrei A, Saluz HP, Pfister W, Schmidtke M. Antipneumococcal activity of neuraminidase inhibiting artocarpin. Int J Med Microbiol. 2015; 305(3): 289-297. DOI: 10.1016/j.ijmm.2014.12.004.

14. van der Vries E, Collins PJ, Vachieri SG, Xiong X, Liu J, Walker PA, Haire LF, Hay AJ, Schutten M, Osterhaus AD, Martin SR, Boucher CA, Skehel JJ, Gamblin SJ. H1N1 2009 pandemic influenza virus: resistance of the I223R neuraminidase mutant explained by kinetic and structural analysis. PLoS Pathog. 2012; 8(9): e1002914. doi: 10.1371/journal.ppat.1002914.

15. Xu G, Li X, Andrew PW, Taylor GL. Structure of the catalytic domain of *Streptococcus pneumoniae* sialidase NanA. Acta Crystallogr Sect F Struct Biol Cryst Commun. 2008; 64(Pt 9): 772-775. doi: 10.1107/S1744309108024044.

16. Gut H, King SJ, Walsh MA. Structural and functional studies of *Streptococcus pneumoniae* neuraminidase B: An intramolecular trans-sialidase. FEBS Lett. 2008; 582(23-24): 3348-3352. doi: 10.1016/j.febslet.2008.08.026.

17. Morris GM, Huey R, Lindstrom W, Sanner MF, Belew RK, Goodsell DS, Olson AJ. AutoDock4 and AutoDockTools4: Automated docking with selective receptor flexibility. J Comput Chem. 2009; 30(16): 2785-2791. doi: 10.1002/jcc.21256.

18. Phillips JC, Hardy DJ, Maia JDC, Stone JE, Ribeiro JV, Bernardi RC, Buch R, Fiorin G, Hénin J, Jiang W, McGreevy R, Melo MCR, Radak BK, Skeel RD, Singharoy A, Wang Y, Roux B, Aksimentiev A, Luthey-Schulten Z, Kalé LV, Schulten K, Chipot C, Tajkhorshid E. Scalable molecular dynamics on CPU and GPU architectures with NAMD. J Chem Phys. 2020; 153(4): 044130. doi: 10.1063/5.0014475.

19. Denning EJ, Priyakumar UD, Nilsson L, Mackerell AD Jr. Impact of 2'-hydroxyl sampling on the conformational properties of RNA: update of the CHARMM all-atom additive force field for RNA. J Comput Chem. 2011; 32(9): 1929-1943. doi: 10.1002/jcc.21777.

20. Best RB, Zhu X, Shim J, Lopes PE, Mittal J, Feig M, Mackerell AD Jr. Optimization of the additive CHARMM all-atom protein force field targeting improved sampling of the backbone φ, ψ and side-chain χ(1) and χ(2) dihedral angles. J Chem Theory Comput. 2012; 8(9): 3257-3273. doi: 10.1021/ct300400x.

21. Vanommeslaeghe K, Hatcher E, Acharya C, Kundu S, Zhong S, Shim J, Darian E, Guvench O, Lopes P, Vorobyov I, Mackerell AD Jr. CHARMM general force field: A force field for drug-like molecules compatible with the CHARMM all-atom additive biological force fields. J Comput Chem. 2010; 31(4): 671-690. doi: 10.1002/jcc.21367.

22. Jorgensen WL, Chandrasekhar J, Madura JD, Impey RW, Klein ML. Comparison of simple potential functions for simulating liquid water. J Chem Phys. 1983; 79: 926-935. doi: 10.1063/1.445869.

23. Stierand K, Maass PC, Rarey M. Molecular complexes at a glance: automated generation of two-dimensional complex diagrams. Bioinformatics. 2006 Jul; 22(14): 1710-1716. doi: 10.1093/bioinformatics/btl150.

24. Fricker PC, Gastreich M, Rarey M. Automated drawing of structural molecular formulas under constraints. J Chem Inf Comput Sci. 2004; 44(3): 1065-1078. doi: 10.1021/ci049958u.

25. Humphrey W, Dalke A, Schulten K. VMD: visual molecular dynamics. J Mol Graph. 1996; 14(1): 33-8, 27-8. doi: 10.1016/0263-7855(96)00018-5.
